# Metrics Selection and Risk Adjustment Methods to Benchmark Inpatient Antibiotic Use

**DOI:** 10.1001/jamanetworkopen.2025.14989

**Published:** 2025-06-11

**Authors:** Michihiko Goto, Hyunkeun Cho, James A. Merchant, Eli N. Perencevich, Matthew B. Goetz, Alexandre R. Marra, Bruce Alexander, Tyler C. Hanks, Brice F. Beck, Christopher Richards, David M. Hernandez, Daniel J. Livorsi

**Affiliations:** 1 Center for Access and Delivery Research and Evaluation, Iowa City Veterans Affairs (VA) Health Care System, Iowa City; 2 Department of Internal Medicine, University of Iowa Carver College of Medicine, Iowa City; 3 Herbert Wertheim School of Public Health, University of California San Diego, La Jolla; 4 Department of Biostatistics, University of Iowa College of Public Health, Iowa City; 5 VA Greater Los Angeles Healthcare System, Los Angeles, California; 6 David Geffen School of Medicine, University of California, Los Angeles; 7 Hospital Israelita Albert Einstein, São Paulo, São Paulo, Brazil

## Abstract

**Importance:**

The Centers for Disease Control and Prevention offers a standardized antimicrobial administration ratio (SAAR) as an evaluation metric for inpatient antibiotic use through rankings and peer comparisons (ie, benchmarking). However, the SAAR model only accounts for facility- and unit-level factors without considering the hierarchical nature of the health care data, and it does not directly reflect patient-level factors or stewardship efforts to avoid overly broad-spectrum therapy.

**Objective:**

To examine the use of antimicrobial use risk adjustment methods and choice of basic metrics (eg, days of therapy [DOT] and days of antimicrobial spectrum coverage [DASC], which do not and do consider antimicrobial spectrum, respectively) in hospital benchmarking.

**Design, Setting, and Participants:**

This retrospective cohort study was conducted using data from 117 acute care hospitals within the Veterans Health Administration (VHA) system. All patients admitted between January 1, 2021, and December 31, 2023, were included.

**Main Outcomes and Measures:**

Monthly antibiotic use was measured with 2 basic metrics and risk adjustment models created using baseline data for 2021 to 2022. Hospitals were benchmarked for 2023 use via 3 methods: (1) unadjusted comparison, (2) risk adjustment with hospital- and unit-level factors with single-level negative binomial regression models (method 1, similar in approach to the SAAR), and (3) risk adjustment with hospital-, unit-, and patient-level factors with hierarchical zero-inflated negative binomial regression models (method 2).

**Results:**

This study included 736 810 patients (median age, 70 [IQR, 61-76] years; 94.7% male). There was wide variability in unadjusted antibiotic use among hospitals (median, 477 [IQR, 420-523] DOT per 1000 days present [DP]; and median, 3115 [IQR, 2739-3602] DASC per 1000 DP). Risk adjustments with methods 1 and 2 resulted in moderate ranking changes, but there were only weak correlations between benchmarking results by the 2 methods (τ_B_ = 0.43 for DOT and 0.44 for DASC). The choice of basic metrics with or without consideration of antimicrobial spectrums (DOT vs DASC) had a modest correlation after risk adjustment (τ_B_ = 0.84).

**Conclusions and Relevance:**

In this cohort study of the nationwide VHA system, there were substantial differences in risk-adjusted benchmarking results between models with only hospital- and unit-level factors and models with hospital-, unit-, and patient-level factors. Future studies should evaluate whether these models with higher content validity also have better construct validity and can inform hospitals and stewardship programs about their objective performance compared with other programs.

## Key Points

**Question:**

When benchmarking inpatient antimicrobial use across different hospitals, how important are patient-level factors for risk adjustments?

**Findings:**

In this cohort study involving 117 hospitals and 736 810 patients, the risk adjustment model that considered hospital-, unit-, and patient-level factors resulted in substantially different benchmarking results from the model based only on hospital- and unit-level factors.

**Meaning:**

These findings suggest that consideration of patient-level factors in risk adjustment models substantially changes benchmarking results compared with a model only with hospital- and unit-level factors.

## Teaser

This cohort study evaluates metrics selection and risk adjustment methods to benchmark inpatient antibiotic use among Veterans Health Administration acute care hospitals.

## Introduction

Benchmarking, often defined as a standardized method for collecting and reporting critical operational data in a way that enables relevant comparisons among the performances of different organizations, is a vital tool for quality improvement programs.^1^ However, benchmarking can be informative only when a metric is measured accurately and relevant to the goals of improving patient care. Therefore, evaluating the validity of metrics is crucial for any quality improvement program.

Without a gold standard, the validity of a metric can be evaluated by assessing 3 forms: face, content, and construct validities. Face validity refers to the extent to which a metric appears to measure what it is intended to at a superficial level. Content validity assesses whether a metric comprehensively covers all aspects of measured activities. Construct validity evaluates whether a metric genuinely measures the theoretical construct it is designed to, ensuring it aligns with related measures and differentiates from unrelated concepts. Together, these validity forms contribute to a metric’s rigor and appropriateness.^2^

For antibiotic stewardship, the most commonly used measure in inpatient settings is days of therapy (DOT) normalized by total care volumes (eg, days present [DP]).^3,4^ However, DOT can be justifiably affected by many factors, such as acuity and types of care provided, procedures performed, or proportions of patients with comorbidities (case-mix effects). Additionally, DOT does not directly consider another important aspect of stewardship activity: avoiding overly broad-spectrum therapy and promoting de-escalation. It can also improperly penalize appropriate combination therapy by counting combined agents separately.^5^ Days of antimicrobial spectrum coverage (DASC) was proposed as a composite metric to combine the length of therapy and spectrum of agents, reflect both use and spectrum quantitatively, and overcome these limitations of DOT.^5,6,7,8^ Although DASC improved face and content validities as an antibiotic use metric, the problem of case-mix effects remains.

Currently, the US Centers for Disease Control and Prevention (CDC) collects DOT data from participating hospitals as part of the antimicrobial use and resistance (AUR) module in the National Healthcare Safety Network (NHSN). It reports a DOT-based risk-adjusted metric—the standardized antimicrobial administration ratio (SAAR)—to provide feedback for antibiotic use compared with peer hospitals.^9^ However, DOT data for SAAR calculation are aggregated at the unit level, and factors included in the risk adjustment method are limited to hospital- and unit-level information; thus, the SAAR has been criticized for not including patient-level data and being potentially inaccurate and prone to ecological bias.^10^ It would be pivotal to consider patient-level factors and reflect all key stewardship activities, including efforts to avoid overuse of broad-spectrum therapy, if they substantially affect program evaluations and benchmarking.

In this study, we used data from the US Veterans Health Administration (VHA) system to examine the use of case-mix adjustment methods and the choice of basic metrics (DOT vs DASC) in hospital benchmarking. Specifically, we developed risk adjustment models using baseline period data (2021-2022), similar to how SAARs were developed using 2017 data, and then compared antibiotic consumption at VHA hospitals using 2023 data and 3 risk adjustment levels: (1) unadjusted comparison per 1000 DP, (2) risk adjustment based on single-level regression models with hospital- and unit-level factors (a similar modeling approach as the SAAR), and (3) risk adjustment based on multilevel regression models with hospital-, unit-, and patient-level factors.

## Methods

The University of Iowa Institutional Review Board approved this cohort study with a waiver of informed consent, due to its retrospective nature and infeasibility to obtain consent from all patients. The study followed the Strengthening the Reporting of Observational Studies in Epidemiology (STROBE) reporting guideline.

### Data Source and Cohort for Hospital Benchmarking of Antibiotic Use

This study included all patients who stayed in acute care wards at 117 VHA hospitals in the US between January 1, 2021, and December 31, 2023. For each calendar month during the study period, we obtained patient-level data from the VHA Corporate Data Warehouse (CDW), the nationwide data repository of VHA electronic medical records. Unit-level characteristics were also obtained from the CDW based on patients’ assigned locations. Hospital-level characteristics were obtained from variables included in the fiscal year 2023 VHA facility complexity model, the VHA’s comprehensive evaluation to define clinical complexity at each medical center based on data from fiscal years 2020 to 2022.^11^ Data for all antibiotic agents listed in the NHSN AUR option were collected.^12^

### Variables for Risk Adjustments

For facility-level variables, we collected data for facility complexity level, facility size, teaching status, intensive care unit (ICU) complexity level, surgical complexity level, and average lengths of stay in acute care for each calendar year. For unit-level variables, we collected data for ICU status (ICU vs non-ICU) and specialty (surgical vs nonsurgical specialties), applying the NHSN AUR module protocol.^12^ We did not use the SAAR identical variable sets because some were unavailable or not applicable for VHA hospitals, effectively leaving only 3 factors as candidate variables (location, ICU bed size, and average length of stay). Factors considered by the adult SAAR model and this study are compared in eTable 1 in Supplement 1.

For patient-level variables, we collected data for patient demographics (age and sex), month of hospital stay, comorbidities, and procedures performed within 48 hours before or during the hospital stay. Comorbidities were obtained as *International Classification of Diseases, Tenth Revision* (*ICD-10*) codes and were classified into 86 categories based on Hierarchical Condition Categories, version 24.^13^ Procedures were obtained from inpatient and outpatient procedure records recorded as *ICD-10* procedure codes or *Current Procedural Terminology* codes. Those codes were classified into 224 categories based on Clinical Classifications Software for Services and Procedures (Agency for Healthcare Research and Quality).^14^ To ensure data availability, potential reproducibility, and generalizability outside the VHA system, we limited patient-level variables to what are commonly available in billing claims in the private sector. Comorbidities and procedures were considered candidate variables if at least 0.5% of included patients had them and had significant correlations with DOT or DASC per 1000 DP in univariate analysis.

### Statistical Analysis

We developed risk adjustment models using baseline period data (2021-2022) and then compared antibiotic use at VHA hospitals using 2023 data and 3 different levels of risk adjustment. First, we aggregated data for DOT, DASC, and DP at each facility and calculated facility-level DOT per 1000 DP and DASC per 1000 DP for the crude unadjusted comparisons.

Second, data were aggregated for each inpatient location to fit a single-level model with the facility- and unit-level factors, using monthly antibiotic use at each inpatient location as a unit of measurement, with separate models for DOT and DASC (method 1). We used negative binomial regression models, and variables were selected by backward elimination to minimize the Akaike information criterion (AIC). DP was included as an offset variable. We fitted these models using the baseline data (2021-2022) and then applied them to the 2023 data to estimate the expected DOT and DASC at each inpatient location in 2023. Observed and expected values for DOT and DASC were aggregated for each facility, and we calculated observed-to-expected (O:E) ratios for all facilities.

Third, we fitted models using each patient as a unit of measurement, again separated for DOT and DASC (method 2). Because of the excessive number of patients who did not receive antibiotics during hospital stays, we used zero-inflated negative binomial regression models with hospital-specific random intercepts both in count and zero-inflation components. This approach allowed us to model 2 components simultaneously with different sets of variables: (1) whether a patient received any antibiotics (zero-inflation component) and (2) how many days of antibiotic therapy a patient received or how broad spectrum the therapy was, if any (count component).^15^ We first selected patient-level candidate variables for both components using the least absolute shrinkage and selection operator. DP was included as an offset variable in count components and as a regular variable in zero-inflation components. To mitigate overfitting and reduce computational burden for a large dataset, we divided our data into 10 random subsets, conducted the model selection process for each subset, and included only variables that were selected across all subsets. Then, facility- and unit-level variables were selected in each subset by backward elimination to minimize AIC while incorporating patient-level variables selected in the previous step; only variables selected in all 10 random subsets were included in the final model. We then fitted models using the 2021-2022 data and then applied fixed effects to the 2023 data with new estimation of random intercepts to calculate 2 estimates for each hospital stay. The first was an estimate with new random intercepts as best linear unbiased predictions (BLUPs), which can be interpreted as facility-, unit-, and patient-specific estimates in 2023. The second was an estimate with random intercept values set to zero, which can be interpreted as the amount of antibiotics one hypothetical standardized hospital would have used for each patient (fixed-effect estimate). Patient-level BLUPs and fixed-effect estimates were aggregated for each facility, and we calculated the ratio between them. Unlike traditional O:E methods using fixed-effect regression models, this approach accounts for the clustering nature of data and has greater specificity (ie, less likely to falsely label hospitals as outliers due to random variation), especially for small hospitals.^16,17,18,19^ A similar method is used by the Centers for Medicare & Medicaid Services (CMS) to calculate the risk-standardized mortality ratio after hospital discharges and is known as the predicted-to-expected (P:E) ratio method.^20,21^

Finally, we compared hospital benchmarking results by calculating the Kendall τ_B_ correlation coefficient for each pair. We interpreted τ_B_ coefficients of 0.7 or greater as strong, between 0.5 and less than 0.7 as moderate, and less than 0.5 as weak correlations and as modest, moderate, and substantial ranking changes, respectively.^22^

Two-sided *P* < .05 was considered significant. All statistical calculations were performed with R, version 4.3.1 (R Project for Statistical Computing), with the pscl, mpath, and glmmTMB packages.^23,24,25^ Details of modeling steps with R codes are available in the eAppendix in Supplement 1.

## Results

This study included 117 VHA hospitals that provided acute inpatient care during the study period (2021-2023) to 736 810 patients at 699 inpatient locations, with a systemwide total of 9 454 177 DP. Patients had a median age of 70 (IQR, 61-76) years; 697 513 (94.7%) were male and 39 297 (5.3%) were female. Hospital-level factors and patient-level characteristics stratified by baseline (2021-2022) and evaluation (2023) periods are summarized in Table 1 and eTable 2 in Supplement 1. No data on patient demographics, comorbidities, and procedures were missing.

**Table 1.** Characteristics of Facilities and Selected Characteristics of Patients^a^

Characteristic	Study period	
	Baseline (2021-2022)	Evaluation (2023)
**Facilities (N = 117)**		
No. of acute care beds, median (IQR)	78 (36.5-109.5)	78 (36.5-109.5)
VA complexity level		
1a (Highest)	38 (32.5)	38 (32.5)
1b	28 (23.9)	28 (23.9)
1c	25 (21.4)	25 (21.4)
2	12 (10.3)	12 (10.3)
3 (Low)	14 (12.0)	14 (12.0)
ICU complexity level		
5 (Most complex)	53 (45.3)	53 (45.3)
4	22 (18.8)	22 (18.8)
3	27 (23.1)	27 (23.1)
2 (Least complex ICU)	3 (2.6)	3 (2.6)
1 (No ICU)	12 (10.3)	12 (10.3)
Surgical complexity score		
4 (Complex inpatient)	69 (59.0)	69 (59.0)
3 (Intermediate inpatient)	27 (23.1)	27 (23.1)
2 (Standard inpatient)	10 (8.5)	10 (8.5)
1 (Advanced ambulatory)	3 (2.6)	3 (2.6)
0.5 (Basic ambulatory)	3 (2.6)	3 (2.6)
0 (No surgery)	5 (4.3)	5 (4.3)
No. of postgraduate trainees per year, median (IQR)	76 (16-168)	76 (16-168)
Average length of stay, median (IQR), d	5.2 (4.4-6.1)	5.0 (4.3-6.1)
No. of wards, median (IQR)	6 (4-8)	6 (4-8)
Total DP, median (IQR)	52 732 (21 689-77 437)	24 865 (11 009-37 189)
DOT		
Total	23 627 (11 528-34 872)	11 912 (5114-17 027)
Per 1000 DP	469.4 (412.2-518.8)	476.8 (419.6-523.2)
DASC, median (IQR)		
Total	146 784 (81 299-236 135)	79 585 (34 320-111 573)
Per 1000 DP	3074 (2682-3544)	3115 (2739-3602)
**Patients (n = 548 195 and 312 477)^b^**		
Age, median (IQR), y	70 (61-76)	71 (62-77)
Sex		
Male	508 514 (92.8)	288 788 (92.4)
Female	39 681 (7.2)	23 689 (7.6)

Abbreviations: DASC, days of antimicrobial spectrum coverage; DOT, days of therapy; DP, days present; ICU, intensive care unit; VA, US Department of Veterans Affairs.

^a^ Unless indicated otherwise, values are presented as the No. (%) of facilities or patients.

^b^ Some patients were included in both cohorts (ie, patients who were hospitalized in 2021-2022 also could be admitted in 2023); a total of 736 810 unique patients comprised the study population. Complete descriptions of patient-level characteristics, including all candidate variables for risk adjustment, are available in eTable 2 in Supplement 1.

**Figure 1.**
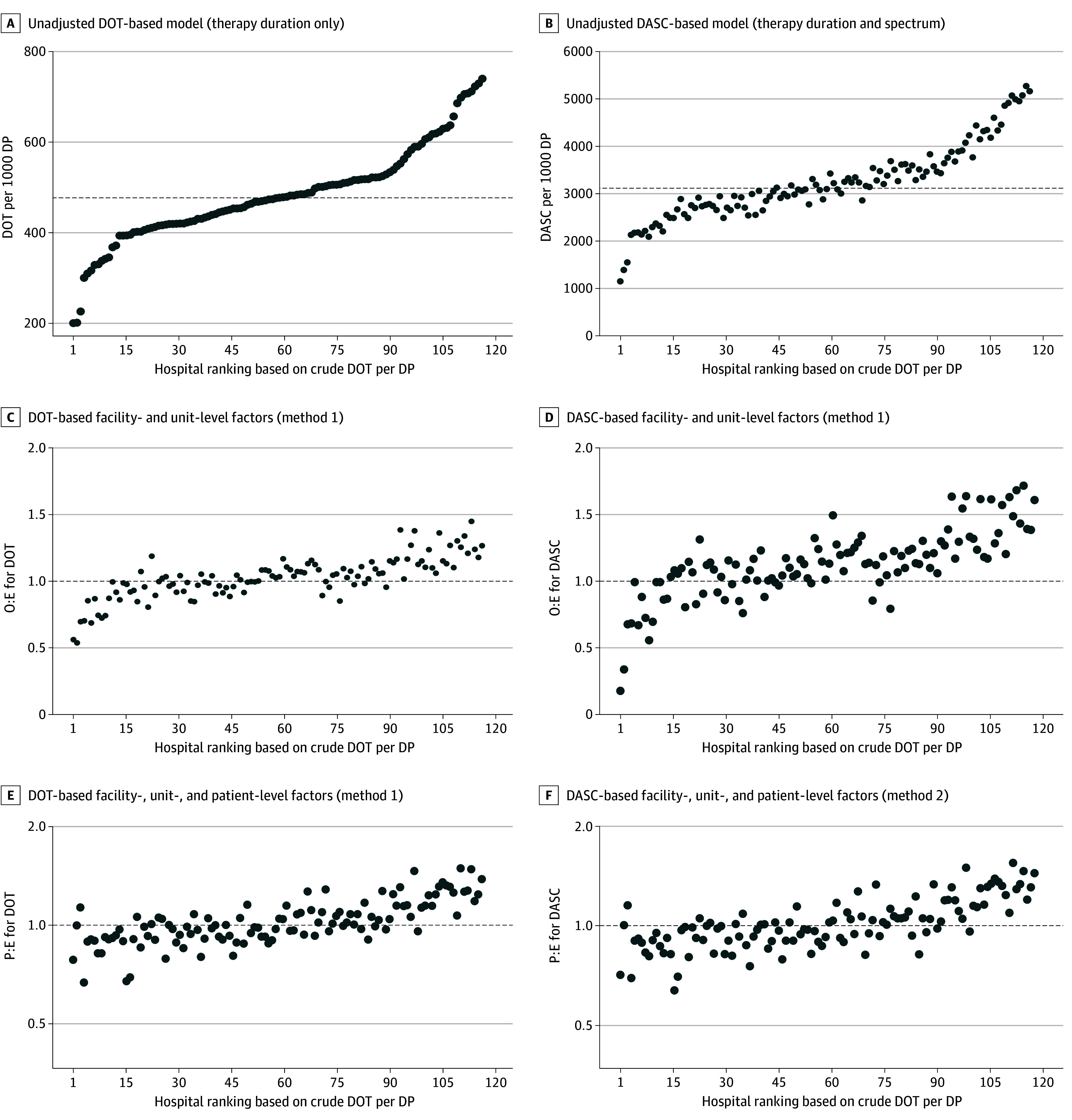
Changes in Hospital Benchmarking Results Based on Selection of Basic Metrics and Risk Adjustment Methods In A and B, dashed lines indicate the median values of days of therapy (DOT) per 1000 days present (DP) or days of antimicrobial spectrum coverage (DASC) per 1000 DP. In C to F, dashed lines indicate an observed-to-expected (O:E) ratio or predicted-to-expected (P:E) ratio equal to 1. Each dot indicates 1 study hospital.

**Figure 2.**
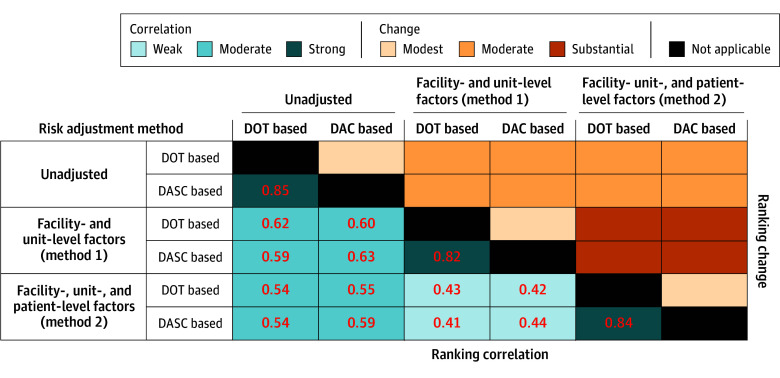
Ranking Correlations by Selection of Basic Metrics and Risk Adjustment Methods Kendall τ_B_ values are presented for correlations (strong, ≥0.7; moderate, 0.5 to <0.7; and weak, <0.5). DASC indicates days of antimicrobial spectrum coverage; DOT, days of therapy.

In crude unadjusted benchmarking for 2023 data, hospital-level DOT per 1000 DP ranged from 200 to 740 (median, 477 [IQR, 420-523]) (Figure 1A), and DASC per 1000 DP ranged from 1150 to 5274 (median, 3115 [IQR, 2739-3602]) (Figure 1B). Considering the antibiotic spectrum in the unadjusted benchmarking by using DASC resulted in a modest change in rankings compared with DOT (τ_B_ = 0.85) (Figure 2).

For method 1 (a similar approach to the SAAR), we selected facility complexity level, ICU complexity level, teaching status, and average length of stay as the facility-level variables and unit type (ICU vs non-ICU) and specialty (surgical vs nonsurgical) as the unit-level variables for both the DOT-based and DASC-based models (Table 2). For the DOT-based model, surgical complexity level was also selected. Risk adjustment using this method resulted in moderate changes in benchmarking rankings, as shown in Figure 1C and D (DOT-based model vs DASC-based model: τ_B_ = 0.61 vs 0.63) (Figure 2). Similar to the unadjusted benchmarking, considering the antibiotic spectrum in the benchmarking with method 1 by using the DASC-based model resulted in a modest change in rankings compared with the DOT-based model (τ_B_ = 0.82) (Figure 2).

**Table 2.** Risk Adjustment Models Using Facility- and Unit-Level Factors (Method 1)

Variable	Rate ratio (95% CI)	*P* value
**DOT-based model**		
Facility level		
VA complexity		
1a (Highest)	1 [Reference]	<.001
1b	0.98 (0.96-1.01)	
1c	0.96 (0.92-1.00)	
2	0.80 (0.74-0.86)	
3 (Low)	0.66 (0.56-0.77)	
ICU complexity		
5 (Most complex)	1 [Reference]	<.001
4	0.91 (0.88-0.94)	
3	1.05 (0.99-1.11)	
2 (Least complex)	1.10 (0.96-1.25)	
1 (No ICU)	0.94 (0.75-1.18)	
Surgical complexity score		
4 (Complex inpatient)	1 [Reference]	<.001
3 (Intermediate inpatient)	0.89 (0.85-0.93)	
2 (Standard inpatient)	0.93 (0.84-1.03)	
1 (Advanced ambulatory)	1.10 (0.84-1.43)	
0.5 (Basic ambulatory)	1.01 (0.79-1.29)	
0 (No surgery)	0.95 (0.76-1.19)	
No. of postgraduate trainees per year, quintile		
1 (Largest)	1 [Reference]	<.001
2	1.13 (1.10-1.16)	
3	1.20 (1.15-1.25)	
4	1.34 (1.26-1.42)	
5 (Smallest)	1.36 (1.23-1.51)	
Unit level		
Average length of stay, quintile		
1 (Shortest)	1 [Reference]	<.001
2	0.87 (0.84-0.90)	
3	0.85 (0.82-0.88)	
4	0.95 (0.92-0.98)	
5 (Longest)	0.86 (0.83-0.89)	
ICU floor	1.57 (1.52-1.62)	<.001
Surgical floor	0.82 (0.80-0.85)	<.001
**DASC-based model**		
Facility level		
VA complexity		
1a (Highest)	1 [Reference]	<.001
1b	0.98 (0.94-1.01)	
1c	0.95 (0.90-1.01)	
2	0.78 (0.72-0.86)	
3 (low)	0.62 (0.51-0.77)	
ICU complexity level		
5 (most complex)	1 [Reference]	<.001
4	0.87 (0.83-0.91)	
3	0.97 (0.91-1.04)	
2 (least complex)	1.13 (0.97-1.31)	
1 (no ICU)	0.99 (0.81-1.21)	
No. of postgraduate trainees per year, quintile		
1 (largest)	1 [Reference]	<.001
2	1.14 (1.10-1.18)	
3	1.20 (1.13-1.27)	
4	1.38 (1.27-1.50)	
5 (smallest)	1.43 (1.26-1.62)	
Unit level		
Average length of stay, quintile		
1 (shortest)	1 [Reference]	<.001
2	0.87 (0.83-0.91)	
3	0.85 (0.82-0.89)	
4	0.94 (0.89-0.98)	
5 (longest)	0.85 (0.81-0.89)	
ICU floor	1.66 (1.59-1.74)	<.001
Surgical floor	0.74 (0.71-0.77)	<.001

Abbreviations: DASC, days of antimicrobial spectrum coverage; DOT, days of therapy; ICU, intensive care unit; VA, US Department of Veterans Affairs.

For method 2, no facility- or unit-level variables were selected in the variable selection process after adjusting for patient-level variables. The final models included patient demographics, ICU admission, surgical specialty admission, month of hospital admission, comorbidities (DOT-based model vs DASC-based model: 32 and 41 conditions vs 35 and 45 conditions in the count and zero-inflation components, respectively), and procedures performed (DOT-based model vs DASC-based model: 87 and 100 procedures vs 95 and 109 procedures in the count and zero-inflation components, respectively). Details of risk adjustment models are available in eTables 3 and 4 in Supplement 1, and distributions of hospital-specific random intercepts are shown in eFigure 1 in Supplement 1. Method 2 resulted in moderate changes in benchmarking rankings compared with unadjusted rankings and in substantial changes compared with rankings based on method 1 (Figure 1E and F), with weak correlations (τ_B_ = 0.43 for DOT and 0.44 for DASC) (Figure 2). Considering the antibiotic spectrum in method 2 by using the DASC-based model again resulted in a moderate change in rankings (τ_B_ = 0.84) (Figure 2).

**Figure 3.**
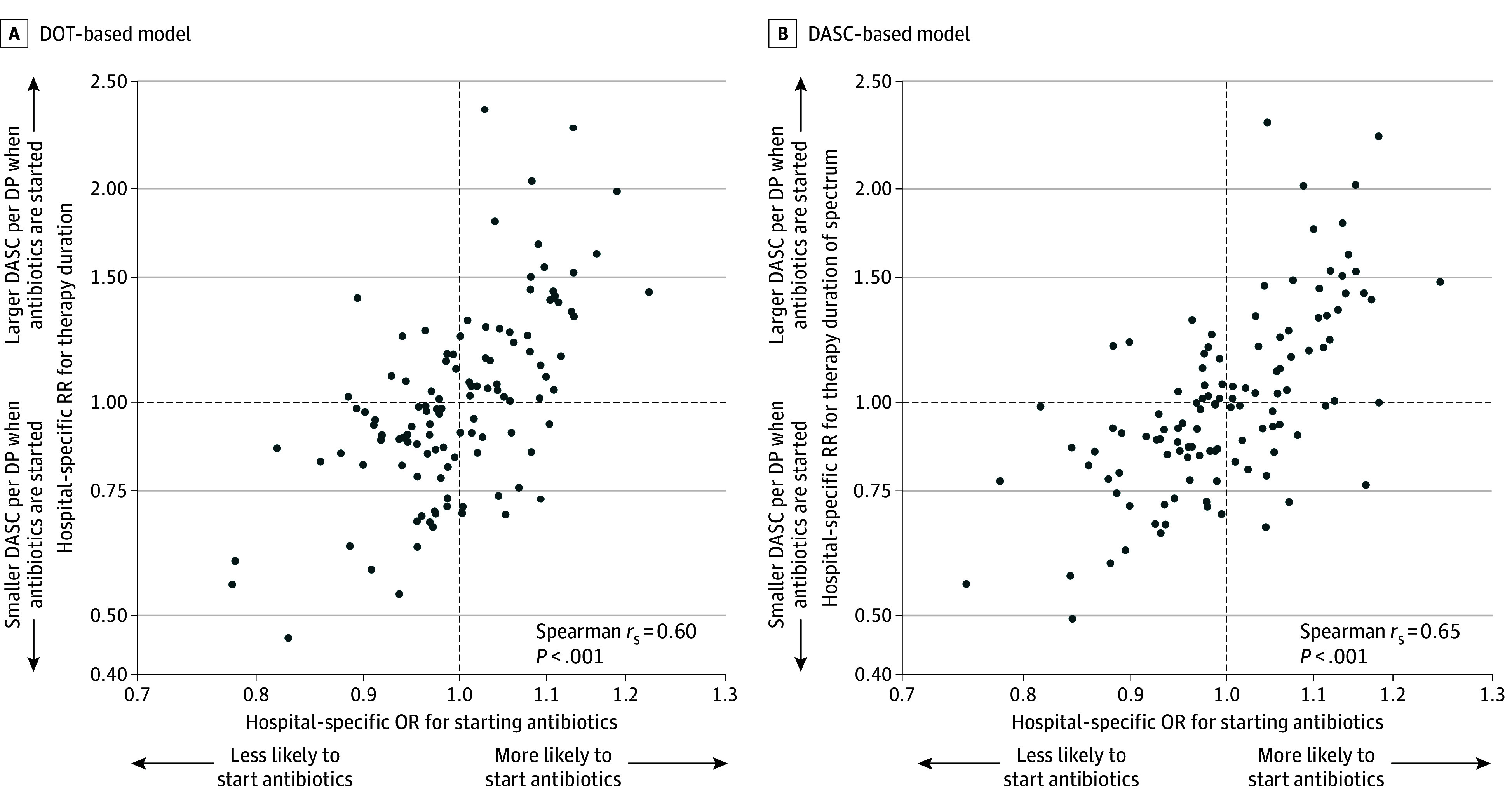
Correlations for Hospital-Specific Random-Effect Estimates Between Count- and Zero-Inflation Components for DOT- and DASC-Based Models Each dot indicates 1 study hospital. DASC indicates days of antimicrobial spectrum coverage; DOT, days of therapy; DP, days present; OR, odds ratio; RR, rate ratio.

When hospital-specific random intercepts for count and zero-inflation components from method 2 were plotted (Figure 3A and B), there was a moderate correlation between them. This finding suggests that hospitals that tend to start antibiotics more frequently for patients with similar risk sets are also likely to use antibiotics longer or use antibiotics with broader spectrums for them.

To assess risk adjustment with method 2, we compared changes in hospital-level random intercept variances between unadjusted and adjusted models using DOT-based and DASC-based methods (eFigures 2 and 3 in Supplement 1). With DOT, adjustment substantially reduced variance in the count component (66.8%) but had a minimal reduction on the zero-inflation component (2.1%). In contrast, the DASC-based model showed a moderate reduction in count variance (49.6%) but a 50.0% increase in zero-inflation variance.

## Discussion

In this study, we benchmarked antibiotic use across 117 VHA hospitals using 2 basic metrics (DOT and DASC) with 2 risk adjustment methods, one with facility- and unit-level factors (method 1, an approach similar to the SAAR but with more detailed risk adjustments) and another that considered all facility-, unit-, and patient-level factors based on widely available administrative code data (method 2). Both methods moderately changed the benchmarking rankings of hospitals, but there were only weak correlations between rankings based on the 2 methods. These results suggest that consideration of patient-level factors (which arguably has more content validity) provides substantially different benchmarking results from models that only consider facility- and unit-level factors, raising concern that the current SAAR methodology may not be providing accurate and valid feedback to participating hospitals. Although the current SAAR methodology was developed with data availability constraints when it was rolled out to all NHSN-participating hospitals nationwide, our findings also suggest that it is likely feasible to apply a method with more content validity using billing claims already utilized by the CMS if billing claims can be made accessible to the CDC.

In this study, considering antibiotic spectrum in the basic metrics (using DASC instead of DOT) resulted in a relatively modest change in rankings compared with risk adjustment methods. However, the remarkable difference in variance reduction patterns between DOT- and DASC-based models suggests that DOT may obscure hospital-level differences in initiation practices, whereas incorporating spectrum through DASC reveals greater unexplained variability in broad-spectrum prescribing. In addition, a 2023 study suggested that DASC has better construct validity by capturing previously missed improvements in hospital antibiotic use practice^8^ and correlating better with stewardship program activities or antibiotic resistance prevalence compared with DOT.^7^ With appropriate risk adjustments, using metrics incorporating antibiotic spectrum (eg, DASC) can further improve validities to assess the performance of stewardship programs.^6^

In method 2, neither our DOT-based nor DASC-based models included facility- or unit-level variables, because they became insignificant and did not improve model fits after adjusting for patient-level variables; hence, they were eliminated in the variable selection processes. This finding suggests that the variability in antibiotic use can be explained better by patient-level factors than hospital- or unit-level factors and, like prior work, supports the need to consider patient-level factors in risk adjustment.^26,27,28^

Other features of our approach in method 2 are the application of random-effect models in estimating antibiotic use and calculation of the P:E rather than the conventional O:E. The random-effect model and the P:E method have been used widely in benchmarking binary outcomes with hierarchical logistic regression models (eg, mortality, readmission).^13,20,29,30,31,32,33^ In addition to appropriate consideration of the hierarchical nature of the data and high specificity for the identification of the aforementioned outliers, the application of this approach to zero-inflated regression models allowed us to extract 2 conceptually different but both clinically meaningful variabilities: the frequency of starting antibiotic therapy (which reflects stewardship efforts in avoiding unnecessary empirical therapy) and the duration or spectrum of antibiotic therapy if any used (which reflects stewardship efforts in avoiding excessively long therapy or unnecessarily broad-spectrum therapy). Therefore, although the P:E ratio can provide overall performance feedback to hospitals, it can also assess their performance in 2 different components separately.

### Limitations

This study has several limitations. First, most patients that the VHA serves are male and older, and it is possible that variables selected for risk adjustments would differ when similar methods are applied outside the VHA. Second, we limited patient-level variables for risk adjustments to demographics and widely available administrative data. Although our decision could improve applicability to and feasibility for non-VHA populations, it is also possible that more detailed clinical data (eg, microbiology results or severity of illness) can affect antibiotic use. Also, we selected variables agnostic to clinical reasoning, and a detailed curation of variables using clinical expertise may improve face validity and content validity. Third, our study did not evaluate the association between risk-adjusted metrics and activities or outcomes of stewardship programs (ie, construct validity). Future studies are needed to assess whether a model with better content validity correlates with stewardship activities or clinical outcomes. Finally, we did not directly compare the SAAR and method 2, because the current SAAR protocol provides only 3 candidate variables for VHA hospitals and we could not perform meaningful risk adjustment. That said, our method 1 used the identical modeling approach with more detailed variables, and this study elucidates the limitation of any antibiotic use metrics that only consider hospital- and unit-level factors.

## Conclusions

In this cohort study, we observed a substantial difference in risk-adjusted benchmarking results between a model with hospital- and unit-level factors (a similar approach to the SAAR) and another model that considered hospital-, unit-, and patient-level factors. This finding raises concern about the validity of the current SAAR methodology. In addition, we observed that it was feasible to build a risk adjustment model with higher content validity that incorporates evaluations of antibiotic use, avoidance of overly broad-spectrum therapy, and patient-level case-mix effects. Future studies should evaluate whether these models with higher content validity also have better construct validity and can inform hospitals and stewardship programs about their objective performance compared with other programs.

## Notes

Supplement 1. **eTable 1.** Comparison of Hospital- and Unit-Level Factors Considered by Adult SAAR and the Current Study **eTable 2.** Complete List of Patient-Level Variables **eTable 3.** Zero-Inflated Negative Binomial Regression Model to Predict Days of Therapy (DOT) for Risk Adjustment With Patient-Level Factors (Method 2) **eTable 4.** Zero-Inflated Negative Binomial Regression Model to Predict Days of Antimicrobial Spectrum Coverage (DASC) for Risk Adjustment With Patient-Level Factors (Method 2) **eFigure 1.** Distribution of Hospital-Specific Random Intercepts With Adjustments **eFigure 2.** Distribution of Hospital-Specific Random Intercepts Before and After Adjustments for DOT-Based Method **eFigure 3.** Distribution of Hospital-Specific Random Intercepts Before and After Adjustments for DASC-Based Method **eAppendix.** Description of Dataset, Statistical Methodologies, and R Codes

Supplement 2.
Data Sharing Statement
