# Supplementary material for: Metrics Selection and Risk Adjustment Methods to Benchmark Inpatient Antibiotic Use
Source: JAMA Netw Open. 2025 Jun 11;8(6):e2514989. doi: 10.1001/jamanetworkopen.2025.14989 (PMC12159770; doi:10.1001/jamanetworkopen.2025.14989)
Supplement: Supplement 1. — eTable 1. Comparison of Hospital- and Unit-Level Factors Considered by Adult SAAR and the Current Study eTable 2. Complete List of Patient-Level Variables eTable 3. Zero-Inflated Negative Binomial Regression Model to Predict Days of Therapy (DOT) for Risk Adjustment With Patient-Level Factors (Method 2) eTable 4. Zero-Inflated Negative Binomial Regression Model to Predict Days of Antimicrobial Spectrum Coverage (DASC) for Risk Adjustment With Patient-Level Factors (Method 2) eFigure 1. Distribution of Hospital-Specific Random Intercepts With Adjustments eFigure 2. Distribution of Hospital-Specific Random Intercepts Before and After Adjustments for DOT-Based Method eFigure 3. Distribution of Hospital-Specific Random Intercepts Before and After Adjustments for DASC-Based Method eAppendix. Description of Dataset, Statistical Methodologies, and R Codes [file jamanetwopen-e2514989-s001.pdf]

Supplemental Online Content

Goto M, Cho H, Merchant JA, et al. Metrics selection and risk adjustment methods to benchmark inpatient antibiotic use. *JAMA Netw Open*. 2025;8(6):e2514989. doi:10.1001/jamanetworkopen.2025.14989

eTable 1. Comparison of Hospital- and Unit-Level Factors Considered by Adult SAAR and the Current Study

eTable 2. Complete List of Patient-Level Variables

eTable 3. Zero-Inflated Negative Binomial Regression Model to Predict Days of Therapy (DOT) for Risk Adjustment With Patient-Level Factors (Method 2)

eTable 4. Zero-Inflated Negative Binomial Regression Model to Predict Days of Antimicrobial Spectrum Coverage (DASC) for Risk Adjustment With Patient-Level Factors (Method 2)

eFigure 1. Distribution of Hospital-Specific Random Intercepts With Adjustments

eFigure 2. Distribution of Hospital-Specific Random Intercepts Before and After Adjustments for DOT-Based Method

eFigure 3. Distribution of Hospital-Specific Random Intercepts Before and After Adjustments for DASC-Based Method

eAppendix. Description of Dataset, Statistical Methodologies, and R Codes

This supplemental material has been provided by the authors to give readers additional information about their work.

© 2025 Goto M et al. *JAMA Network Open*.

Supplementary Table 1. Comparison of Hospital- and Unit-Level Factors Considered by Adult SAAR and the Current Study

Factors		Adult SAAR Model	The Current Study	Comments
Location	Medical ICU	**Yes**	**Yes**	
	Medical/Surgical or Surgical ICU	**Yes**	**Yes**	
	General Hematology/Oncology Ward	**Yes**	No	Only two VA hospitals had dedicated hematology/oncology inpatient wards
	Step-Down Unit	**Yes**	No	No ward was formally designated as step-down unit in VA system
	Medical Ward	**Yes**	No	Surgical ward was considered as the reference
	Surgical Ward	No	**Yes**	Medical ward was considered as the reference
Hospital Type	Veteran's Affairs Hospital	**Yes**	N/A	
	Critical Access Hospital	**Yes**	N/A	
	Women's hospital	**Yes**	N/A	
	Hospital Complexity	No	**Yes**	5-level classifications based on hospital size, care complexities, and patient populations
ICU Size/Complexity	ICU Bed Size	**Yes**	No	Dichotomous categorization by >=8 or <8 ICU beds
	ICU Complexity	No	**Yes**	5-level classification based on bed size, capability, and case-mix
Surgical Care	Surgical Complexity	No	**Yes**	5-level classification based on the types of surgical services provided.
Acuity	Average Length of Stay	**Yes**	**Yes**	For SAAR, dichotomous categorization by >=3.6 or <3.6; for the current study, five-level categorization by quintile.
Teaching Status	Teaching Facility	**Yes**	**Yes**	For SAAR, dichotomous categorization by presence or absence of teaching program; for the current study, five-level categorization by quintile of the numbers of trainees at each hospital, as all VA acute care hospitals have academic affiliations at various degrees.

Abbreviations: ICU: Intensive Care Unit; SAAR: Standardized Antimicrobial Administration Ratio; VA: Department of Veterans Affairs

© 2025 Goto M et al. JAMA Network Open.

Supplementary Table 2. Complete List of Patient-Level Variables

Variable	Description	2021-2022 (Baseline)	2023 (Evaluation)
Patient Demographics			
**n**	Number of Unique Patients During Study Period	548,195	312,477
**Age***	Median (Interquartile Range)	70 (61-76)	71 (62-77)
**Male Gender**		508,514 (92.8%)	288,788 (92.4%)
Hierarchical Condition Categories (HCC) v24 for Chronic Medical Conditions			
**HCC Category 1**	HIV/AIDS	6,130 (1.1%)	3,645 (1.2%)
**HCC Category 2**	Septicemia, Sepsis, Systemic Inflammatory Response Syndrome/Shock	77,911 (14.2%)	44,421 (14.2%)
**HCC Category 6**	Opportunistic Infections	6,792 (1.2%)	3,955 (1.3%)
**HCC Category 8**	Metastatic Cancer and Acute Leukemia	35,318 (6.4%)	19,875 (6.4%)
**HCC Category 9**	Lung and Other Severe Cancers	42,231 (7.7%)	24,154 (7.7%)
**HCC Category 10**	Lymphoma and Other Cancers	22,756 (4.2%)	13,470 (4.3%)
**HCC Category 11**	Colorectal, Bladder, and Other Cancers	41,749 (7.6%)	24,329 (7.8%)
**HCC Category 12**	Breast, Prostate, and Other Cancers and Tumors	68,175 (12.4%)	41,333 (13.2%)
**HCC Category 17**	Diabetes with Acute Complications	10,295 (1.9%)	5,606 (1.8%)
**HCC Category 18**	Diabetes with Chronic Complications	188,422 (34.4%)	110,839 (35.5%)
**HCC Category 19**	Diabetes without Complication	215,844 (39.4%)	125,851 (40.3%)
**HCC Category 21**	Protein-Calorie Malnutrition	58,511 (10.7%)	33,579 (10.7%)
**HCC Category 22**	Morbid Obesity	51,144 (9.3%)	28,347 (9.1%)
**HCC Category 23**	Other Significant Endocrine and Metabolic Disorders	49,339 (9.0%)	30,396 (9.7%)
**HCC Category 27**	End-Stage Liver Disease	16,241 (3.0%)	9,256 (3.0%)
**HCC Category 28**	Cirrhosis of Liver	33,881 (6.2%)	19,609 (6.3%)
**HCC Category 29**	Chronic Hepatitis	27,629 (5.0%)	14,883 (4.8%)
**HCC Category 33**	Intestinal Obstruction/Perforation	31,661 (5.8%)	18,530 (5.9%)
**HCC Category 34**	Chronic Pancreatitis	8,534 (1.6%)	5,083 (1.6%)
**HCC Category 35**	Inflammatory Bowel Disease	12,509 (2.3%)	7,165 (2.3%)
**HCC Category 39**	Bone/Joint/Muscle Infections/Necrosis	33,415 (6.1%)	20,311 (6.5%)
**HCC Category 40**	Rheumatoid Arthritis and Inflammatory Connective Tissue Disease	29,124 (5.3%)	17,922 (5.7%)
**HCC Category 46**	Severe Hematological Disorders	6,798 (1.2%)	3,642 (1.2%)
**HCC Category 47**	Disorders of Immunity	24,947 (4.6%)	15,150 (4.8%)
**HCC Category 48**	Coagulation Defects and Other Specified Hematological Disorders	65,035 (11.9%)	35,851 (11.5%)
**HCC Category 51**	Dementia With Complications	5,708 (1.0%)	13,796 (4.4%)

© 2025 Goto M et al. JAMA Network Open.

**HCC Category 52**	Dementia Without Complication	57,314 (10.5%)	32,448 (10.4%)
**HCC Category 54**	Drug/Alcohol Psychosis	11,936 (2.2%)	6,618 (2.1%)
**HCC Category 55**	Drug/Alcohol Dependence	106,781 (19.5%)	62,041 (19.9%)
**HCC Category 56**	Major Depressive, Bipolar, and Paranoid Disorders	32,107 (5.9%)	18,537 (5.9%)
**HCC Category 57**	Schizophrenia	18,468 (3.4%)	10,494 (3.4%)
**HCC Category 58**	Reactive and Unspecified Psychosis	9,456 (1.7%)	5,265 (1.7%)
**HCC Category 59**	Personality Disorders	164,436 (30.0%)	96,456 (30.9%)
**HCC Category 60**	Eating Disorders	14,406 (2.6%)	8,441 (2.7%)
**HCC Category 70**	Quadriplegia	5,836 (1.1%)	3,398 (1.1%)
**HCC Category 71**	Paraplegia	5,130 (0.9%)	3,109 (1.0%)
**HCC Category 72**	Spinal Cord Disorders/Injuries	12,195 (2.2%)	7,676 (2.5%)
**HCC Category 73**	Amyotrophic Lateral Sclerosis and Other Motor Neuron Disease	1,313 (0.2%)	752 (0.2%)
**HCC Category 74**	Cerebral Palsy	104 (0.0%)	59 (0.0%)
**HCC Category 75**	Myasthenia Gravis/Myoneural Disorders, Inflammatory and Toxic Neuropathy	9,385 (1.7%)	5,541 (1.8%)
**HCC Category 76**	Muscular Dystrophy	300 (0.1%)	197 (0.1%)
**HCC Category 77**	Multiple Sclerosis	3,595 (0.7%)	2,069 (0.7%)
**HCC Category 78**	Parkinson's and Huntington's Diseases	14,654 (2.7%)	9,323 (3.0%)
**HCC Category 79**	Seizure Disorders and Convulsions	30,441 (5.6%)	18,094 (5.8%)
**HCC Category 80**	Coma, Brain Compression/Anoxic Damage	3,196 (0.6%)	1,676 (0.5%)
**HCC Category 82**	Respirator Dependence/Tracheostomy Status	6,991 (1.3%)	3,347 (1.1%)
**HCC Category 83**	Respiratory Arrest	454 (0.1%)	209 (0.1%)
**HCC Category 84**	Cardio-Respiratory Failure and Shock	116,741 (21.3%)	60,333 (19.3%)
**HCC Category 85**	Congestive Heart Failure	162,363 (29.6%)	97,200 (31.1%)
**HCC Category 86**	Acute Myocardial Infarction	49,772 (9.1%)	28,469 (9.1%)
**HCC Category 87**	Unstable Angina and Other Acute Ischemic Heart Disease	33,016 (6.0%)	16,989 (5.4%)
**HCC Category 88**	Angina Pectoris	57,377 (10.5%)	32,784 (10.5%)
**HCC Category 96**	Specified Heart Arrhythmias	146,931 (26.8%)	90,748 (29.0%)
**HCC Category 99**	Cerebral Hemorrhage	6,653 (1.2%)	3,824 (1.2%)
**HCC Category 100**	Ischemic or Unspecified Stroke	50,112 (9.1%)	29,679 (9.5%)
**HCC Category 103**	Hemiplegia/Hemiparesis	22,756 (4.2%)	13,274 (4.2%)
**HCC Category 104**	Monoplegia, Other Paralytic Syndromes	4,400 (0.8%)	2,483 (0.8%)
**HCC Category 106**	Atherosclerosis of the Extremities with Ulceration or Gangrene	16,604 (3.0%)	9,342 (3.0%)
**HCC Category 107**	Vascular Disease with Complications	43,720 (8.0%)	24,799 (7.9%)
**HCC Category 108**	Vascular Disease	125,187 (22.8%)	81,947 (26.2%)

© 2025 Goto M et al. JAMA Network Open.

**HCC Category 110**	Cystic Fibrosis	60 (0.0%)	40 (0.0%)
**HCC Category 111**	Chronic Obstructive Pulmonary Disease	165,796 (30.2%)	95,260 (30.5%)
**HCC Category 112**	Fibrosis of Lung and Other Chronic Lung Disorders	26,641 (4.9%)	16,633 (5.3%)
**HCC Category 114**	Aspiration and Specified Bacterial Pneumonias	28,211 (5.1%)	15,906 (5.1%)
**HCC Category 115**	Pneumococcal Pneumonia, Empyema, Lung Abscess	15,290 (2.8%)	9,118 (2.9%)
**HCC Category 122**	Proliferative Diabetic Retinopathy and Vitreous Hemorrhage	7,575 (1.4%)	4,581 (1.5%)
**HCC Category 124**	Exudative Macular Degeneration	6,370 (1.2%)	4,038 (1.3%)
**HCC Category 134**	Dialysis Status	16,121 (2.9%)	9,504 (3.0%)
**HCC Category 135**	Acute Renal Failure	156,653 (28.6%)	87,762 (28.1%)
**HCC Category 136**	Chronic Kidney Disease, Stage 5	23,332 (4.3%)	13,718 (4.4%)
**HCC Category 137**	Chronic Kidney Disease, Severe (Stage 4)	27,378 (5.0%)	16,864 (5.4%)
**HCC Category 138**	Chronic Kidney Disease, Moderate (Stage 3)	94,342 (17.2%)	59,710 (19.1%)
**HCC Category 157**	Pressure Ulcer of Skin with Necrosis Through to Muscle, Tendon, or Bone	4,862 (0.9%)	2,841 (0.9%)
**HCC Category 158**	Pressure Ulcer of Skin with Full Thickness Skin Loss	15,001 (2.7%)	8,365 (2.7%)
**HCC Category 159**	Major Skin Burn or Condition	16,625 (3.0%)	9,147 (2.9%)
**HCC Category 161**	Chronic Ulcer of Skin, Except Pressure	43,295 (7.9%)	25,611 (8.2%)
**HCC Category 162**	Severe Skin Burn or Condition	245 (0.0%)	120 (0.0%)
**HCC Category 166**	Severe Head Injury	255 (0.0%)	108 (0.0%)
**HCC Category 167**	Major Head Injury	8,763 (1.6%)	5,113 (1.6%)
**HCC Category 169**	Vertebral Fractures without Spinal Cord Injury	9,864 (1.8%)	6,049 (1.9%)
**HCC Category 170**	Hip Fracture/Dislocation	8,191 (1.5%)	4,537 (1.5%)
**HCC Category 173**	Traumatic Amputations and Complications	3,289 (0.6%)	1,807 (0.6%)
**HCC Category 176**	Complications of Specified Implanted Device or Graft	34,304 (6.3%)	20,105 (6.4%)
**HCC Category 186**	Major Organ Transplant or Replacement Status	4,076 (0.7%)	2,541 (0.8%)
**HCC Category 188**	Artificial Openings for Feeding or Elimination	20,728 (3.8%)	11,921 (3.8%)
**HCC Category 189**	Amputation Status, Lower Limb/Amputation Complications	20,168 (3.7%)	12,493 (4.0%)
Clinical Classifications Software (CCS) for Procedures Performed			
**CCS Category 1**	Incision and excision of CNS	758 (0.1%)	368 (0.1%)
**CCS Category 2**	Insertion, replacement, or removal of extracranial ventricular shunt	201 (0.0%)	88 (0.0%)
**CCS Category 3**	Laminectomy, excision intervertebral disc	6,284 (1.1%)	3,101 (1.0%)
**CCS Category 4**	Diagnostic spinal tap	3,625 (0.7%)	1,745 (0.6%)
**CCS Category 5**	Insertion of catheter or spinal stimulator and injection into spinal canal	6,943 (1.3%)	3,187 (1.0%)
**CCS Category 6**	Decompression peripheral nerve	319 (0.1%)	177 (0.1%)
**CCS Category 7**	Other diagnostic nervous system procedures	3,377 (0.6%)	1,708 (0.5%)

© 2025 Goto M et al. JAMA Network Open.

**CCS Category 8**	Other non-OR or closed therapeutic nervous system procedures	18,766 (3.4%)	10,342 (3.3%)
**CCS Category 9**	Other OR therapeutic nervous system procedures	2,004 (0.4%)	1,153 (0.4%)
**CCS Category 10**	Thyroidectomy, partial or complete	1,855 (0.3%)	949 (0.3%)
**CCS Category 11**	Diagnostic endocrine procedures	688 (0.1%)	427 (0.1%)
**CCS Category 12**	Other therapeutic endocrine procedures	1,393 (0.3%)	797 (0.3%)
**CCS Category 14**	Glaucoma procedures	233 (0.0%)	114 (0.0%)
**CCS Category 15**	Lens and cataract procedures	1,113 (0.2%)	663 (0.2%)
**CCS Category 16**	Repair of retinal tear, detachment	184 (0.0%)	88 (0.0%)
**CCS Category 18**	Diagnostic procedures on eye	1,114 (0.2%)	313 (0.1%)
**CCS Category 19**	Other therapeutic procedures on eyelids, conjunctiva, cornea	585 (0.1%)	303 (0.1%)
**CCS Category 20**	Other intraocular therapeutic procedures	711 (0.1%)	365 (0.1%)
**CCS Category 25**	Diagnostic procedures on ear	395 (0.1%)	169 (0.1%)
**CCS Category 26**	Other therapeutic ear procedures	1,841 (0.3%)	773 (0.2%)
**CCS Category 27**	Control of epistaxis	452 (0.1%)	197 (0.1%)
**CCS Category 28**	Plastic procedures on nose	838 (0.2%)	405 (0.1%)
**CCS Category 29**	Oral and Dental Services	1,170 (0.2%)	539 (0.2%)
**CCS Category 30**	Tonsillectomy and/or adenoidectomy	317 (0.1%)	180 (0.1%)
**CCS Category 31**	Diagnostic procedures on nose, mouth and pharynx	67,993 (12.4%)	37,099 (11.9%)
**CCS Category 32**	Other non-OR therapeutic procedures on nose, mouth and pharynx	30,448 (5.6%)	16,049 (5.1%)
**CCS Category 33**	Other OR therapeutic procedures on nose, mouth and pharynx	3,045 (0.6%)	1,473 (0.5%)
**CCS Category 34**	Tracheostomy, temporary and permanent	1,784 (0.3%)	671 (0.2%)
**CCS Category 35**	Tracheoscopy and laryngoscopy with biopsy	4,334 (0.8%)	2,040 (0.7%)
**CCS Category 36**	Lobectomy or pneumonectomy	3,384 (0.6%)	1,610 (0.5%)
**CCS Category 37**	Diagnostic bronchoscopy and biopsy of bronchus	8,855 (1.6%)	4,178 (1.3%)
**CCS Category 38**	Other diagnostic procedures on lung and bronchus	23,389 (4.3%)	11,264 (3.6%)
**CCS Category 39**	Incision of pleura, thoracentesis, chest drainage	11,178 (2.0%)	5,321 (1.7%)
**CCS Category 40**	Other diagnostic procedures of respiratory tract and mediastinum	1,021 (0.2%)	386 (0.1%)
**CCS Category 41**	Other non-OR therapeutic procedures on respiratory system	75,002 (13.7%)	37,106 (11.9%)
**CCS Category 42**	Other OR therapeutic procedures on respiratory system	3,107 (0.6%)	1,584 (0.5%)
**CCS Category 43**	Heart valve procedures	2,996 (0.5%)	1,498 (0.5%)
**CCS Category 44**	Coronary artery bypass graft (CABG)	3,958 (0.7%)	2,062 (0.7%)
**CCS Category 45**	Percutaneous transluminal coronary angioplasty (PTCA)	13,154 (2.4%)	6,039 (1.9%)
**CCS Category 46**	Coronary thrombolysis	143 (0.0%)	103 (0.0%)
**CCS Category 47**	Diagnostic cardiac catheterization, coronary arteriography	35,666 (6.5%)	17,681 (5.7%)

© 2025 Goto M et al. JAMA Network Open.

**CCS Category 48**	Insertion, revision, replacement, removal of cardiac pacemaker or cardioverter/defibrillator	10,276 (1.9%)	5,229 (1.7%)
**CCS Category 49**	Other OR heart procedures	4,497 (0.8%)	2,309 (0.7%)
**CCS Category 50**	Extracorporeal circulation auxiliary to open heart procedures	848 (0.2%)	427 (0.1%)
**CCS Category 51**	Endarterectomy, vessel of head and neck	2,778 (0.5%)	1,278 (0.4%)
**CCS Category 52**	Aortic resection, replacement or anastomosis	2,288 (0.4%)	1,253 (0.4%)
**CCS Category 54**	Other vascular catheterization, not heart	139,487 (25.4%)	73,807 (23.6%)
**CCS Category 55**	Peripheral vascular bypass	3,020 (0.6%)	1,399 (0.4%)
**CCS Category 56**	Other vascular bypass and shunt, not heart	396 (0.1%)	202 (0.1%)
**CCS Category 57**	Creation, revision and removal of arteriovenous fistula or vessel-to-vessel cannula for dialysis	3,321 (0.6%)	1,667 (0.5%)
**CCS Category 58**	Hemodialysis	15,835 (2.9%)	8,680 (2.8%)
**CCS Category 59**	Other OR procedures on vessels of head and neck	1,911 (0.3%)	970 (0.3%)
**CCS Category 60**	Embolectomy and endarterectomy of lower limbs	3,421 (0.6%)	1,648 (0.5%)
**CCS Category 61**	Other OR procedures on vessels other than head and neck	20,653 (3.8%)	10,110 (3.2%)
**CCS Category 62**	Other diagnostic cardiovascular procedures	29,436 (5.4%)	14,868 (4.8%)
**CCS Category 63**	Other non-OR therapeutic cardiovascular procedures	17,632 (3.2%)	8,930 (2.9%)
**CCS Category 64**	Bone marrow transplant	308 (0.1%)	130 (0.0%)
**CCS Category 65**	Bone marrow biopsy	2,278 (0.4%)	1,077 (0.3%)
**CCS Category 66**	Procedures on spleen	161 (0.0%)	68 (0.0%)
**CCS Category 67**	Other therapeutic procedures, hemic and lymphatic system	9,615 (1.8%)	4,545 (1.5%)
**CCS Category 68**	Injection or ligation of esophageal varices	771 (0.1%)	365 (0.1%)
**CCS Category 69**	Esophageal dilatation	1,557 (0.3%)	871 (0.3%)
**CCS Category 70**	Upper gastrointestinal endoscopy, biopsy	33,181 (6.1%)	16,769 (5.4%)
**CCS Category 71**	Gastrostomy, temporary and permanent	5,117 (0.9%)	2,416 (0.8%)
**CCS Category 72**	Colostomy, temporary and permanent	598 (0.1%)	293 (0.1%)
**CCS Category 73**	Ileostomy and other enterostomy	1,500 (0.3%)	662 (0.2%)
**CCS Category 74**	Gastrectomy, partial and total	678 (0.1%)	233 (0.1%)
**CCS Category 75**	Small bowel resection	1,486 (0.3%)	691 (0.2%)
**CCS Category 76**	Colonoscopy and biopsy	18,372 (3.4%)	9,556 (3.1%)
**CCS Category 77**	Proctoscopy and anorectal biopsy	4,407 (0.8%)	2,234 (0.7%)
**CCS Category 78**	Colorectal resection	5,787 (1.1%)	2,838 (0.9%)
**CCS Category 80**	Appendectomy	2,888 (0.5%)	1,459 (0.5%)
**CCS Category 81**	Hemorrhoid procedures	386 (0.1%)	188 (0.1%)
**CCS Category 82**	Endoscopic retrograde cannulation of pancreas (ERCP)	3,509 (0.6%)	1,685 (0.5%)
**CCS Category 83**	Biopsy of liver	2,537 (0.5%)	1,194 (0.4%)

© 2025 Goto M et al. JAMA Network Open.

**CCS Category 84**	Cholecystectomy and common duct exploration	6,201 (1.1%)	3,199 (1.0%)
**CCS Category 85**	Inguinal and femoral hernia repair	2,896 (0.5%)	1,601 (0.5%)
**CCS Category 86**	Other hernia repair	5,058 (0.9%)	618 (0.2%)
**CCS Category 87**	Laparoscopy	1,261 (0.2%)	683 (0.2%)
**CCS Category 88**	Abdominal paracentesis	7,457 (1.4%)	3,731 (1.2%)
**CCS Category 89**	Exploratory laparotomy	922 (0.2%)	489 (0.2%)
**CCS Category 90**	Excision, lysis peritoneal adhesions	493 (0.1%)	277 (0.1%)
**CCS Category 91**	Peritoneal dialysis	10,148 (1.9%)	5,881 (1.9%)
**CCS Category 92**	Other bowel diagnostic procedures	9,351 (1.7%)	4,301 (1.4%)
**CCS Category 93**	Other non-OR upper GI therapeutic procedures	3,855 (0.7%)	1,718 (0.5%)
**CCS Category 94**	Other OR upper GI therapeutic procedures	1,532 (0.3%)	735 (0.2%)
**CCS Category 95**	Other non-OR lower GI therapeutic procedures	3,599 (0.7%)	1,825 (0.6%)
**CCS Category 96**	Other OR lower GI therapeutic procedures	3,191 (0.6%)	1,536 (0.5%)
**CCS Category 97**	Other gastrointestinal diagnostic procedures	6,636 (1.2%)	3,369 (1.1%)
**CCS Category 98**	Other non-OR gastrointestinal therapeutic procedures	11,369 (2.1%)	5,766 (1.8%)
**CCS Category 99**	Other OR gastrointestinal therapeutic procedures	5,414 (1.0%)	2,690 (0.9%)
**CCS Category 100**	Endoscopy and endoscopic biopsy of the urinary tract	3,675 (0.7%)	1,874 (0.6%)
**CCS Category 101**	Transurethral excision, drainage, or removal urinary obstruction	4,559 (0.8%)	2,394 (0.8%)
**CCS Category 102**	Ureteral catheterization	6,369 (1.2%)	3,097 (1.0%)
**CCS Category 103**	Nephrotomy and nephrostomy	3,114 (0.6%)	1,658 (0.5%)
**CCS Category 104**	Nephrectomy, partial or complete	2,988 (0.5%)	1,641 (0.5%)
**CCS Category 105**	Kidney transplant	367 (0.1%)	312 (0.1%)
**CCS Category 106**	Genitourinary incontinence procedures	586 (0.1%)	235 (0.1%)
**CCS Category 107**	Extracorporeal lithotripsy, urinary	2,459 (0.4%)	1,131 (0.4%)
**CCS Category 108**	Indwelling catheter	13,720 (2.5%)	5,777 (1.8%)
**CCS Category 109**	Procedures on the urethra	1,226 (0.2%)	549 (0.2%)
**CCS Category 110**	Other diagnostic procedures of urinary tract	1,922 (0.4%)	918 (0.3%)
**CCS Category 111**	Other non-OR therapeutic procedures of urinary tract	5,956 (1.1%)	2,937 (0.9%)
**CCS Category 112**	Other OR therapeutic procedures of urinary tract	3,803 (0.7%)	1,822 (0.6%)
**CCS Category 113**	Transurethral resection of prostate (TURP)	5,325 (1.0%)	2,616 (0.8%)
**CCS Category 114**	Open prostatectomy	4,413 (0.8%)	1,717 (0.5%)
**CCS Category 116**	Diagnostic procedures, male genital	676 (0.1%)	344 (0.1%)
**CCS Category 117**	Other non-OR therapeutic procedures, male genital	559 (0.1%)	216 (0.1%)
**CCS Category 118**	Other OR therapeutic procedures, male genital	3,329 (0.6%)	1,581 (0.5%)

© 2025 Goto M et al. JAMA Network Open.

**CCS Category 124**	Hysterectomy, abdominal and vaginal	1,588 (0.3%)	775 (0.2%)
**CCS Category 130**	Other diagnostic procedures, female organs	239 (0.0%)	134 (0.0%)
**CCS Category 132**	Other OR therapeutic procedures, female organs	237 (0.0%)	123 (0.0%)
**CCS Category 142**	Partial excision bone	2,276 (0.4%)	1,117 (0.4%)
**CCS Category 143**	Bunionectomy or repair of toe deformities	148 (0.0%)	91 (0.0%)
**CCS Category 144**	Treatment, facial fracture or dislocation	347 (0.1%)	170 (0.1%)
**CCS Category 145**	Treatment, fracture or dislocation of radius and ulna	297 (0.1%)	150 (0.0%)
**CCS Category 146**	Treatment, fracture or dislocation of hip and femur	2,962 (0.5%)	1,372 (0.4%)
**CCS Category 147**	Treatment, fracture or dislocation of lower extremity (other than hip or femur)	1,367 (0.2%)	604 (0.2%)
**CCS Category 148**	Other fracture and dislocation procedure	945 (0.2%)	528 (0.2%)
**CCS Category 149**	Arthroscopy	270 (0.0%)	121 (0.0%)
**CCS Category 151**	Excision of semilunar cartilage of knee	143 (0.0%)	77 (0.0%)
**CCS Category 152**	Arthroplasty knee	14,057 (2.6%)	8,198 (2.6%)
**CCS Category 153**	Hip replacement, total and partial	8,013 (1.5%)	4,189 (1.3%)
**CCS Category 154**	Arthroplasty other than hip or knee	3,144 (0.6%)	1,888 (0.6%)
**CCS Category 155**	Arthrocentesis	3,369 (0.6%)	1,628 (0.5%)
**CCS Category 156**	Injections and aspirations of muscles, tendons, bursa, joints and soft tissue	2,023 (0.4%)	996 (0.3%)
**CCS Category 157**	Amputation of lower extremity	9,141 (1.7%)	4,728 (1.5%)
**CCS Category 158**	Spinal fusion	5,147 (0.9%)	2,712 (0.9%)
**CCS Category 159**	Other diagnostic procedures on musculoskeletal system	5,845 (1.1%)	2,919 (0.9%)
**CCS Category 160**	Other therapeutic procedures on muscles and tendons	3,994 (0.7%)	2,096 (0.7%)
**CCS Category 161**	Other OR therapeutic procedures on bone	5,847 (1.1%)	3,173 (1.0%)
**CCS Category 162**	Other OR therapeutic procedures on joints	2,173 (0.4%)	1,114 (0.4%)
**CCS Category 163**	Other non-OR therapeutic procedures on musculoskeletal system	1,497 (0.3%)	664 (0.2%)
**CCS Category 164**	Other OR therapeutic procedures on musculoskeletal system	897 (0.2%)	622 (0.2%)
**CCS Category 167**	Mastectomy	532 (0.1%)	243 (0.1%)
**CCS Category 168**	Incision and drainage, skin and subcutaneous tissue	6,661 (1.2%)	3,225 (1.0%)
**CCS Category 169**	Debridement of wound, infection or burn	9,358 (1.7%)	4,709 (1.5%)
**CCS Category 170**	Excision of skin lesion	5,937 (1.1%)	3,051 (1.0%)
**CCS Category 171**	Suture of skin and subcutaneous tissue	2,338 (0.4%)	1,104 (0.4%)
**CCS Category 172**	Skin graft	3,068 (0.6%)	1,489 (0.5%)
**CCS Category 173**	Other diagnostic procedures on skin and subcutaneous tissue	5,527 (1.0%)	2,491 (0.8%)
**CCS Category 174**	Other non-OR therapeutic procedures on skin and breast	16,416 (3.0%)	8,852 (2.8%)
**CCS Category 175**	Other OR therapeutic procedures on skin and breast	2,925 (0.5%)	1,686 (0.5%)

© 2025 Goto M et al. JAMA Network Open.

**CCS Category 176**	Other organ transplantation	108 (0.0%)	80 (0.0%)
**CCS Category 177**	Computerized axial tomography (CT) scan head	141,482 (25.8%)	78,093 (25.0%)
**CCS Category 178**	CT scan chest	139,720 (25.5%)	74,675 (23.9%)
**CCS Category 179**	CT scan abdomen	155,749 (28.4%)	88,374 (28.3%)
**CCS Category 180**	Other CT scan	70,765 (12.9%)	36,904 (11.8%)
**CCS Category 181**	Myelogram	517 (0.1%)	256 (0.1%)
**CCS Category 182**	Mammography	321 (0.1%)	147 (0.0%)
**CCS Category 183**	Routine chest X-ray	363,164 (66.2%)	194,749 (62.3%)
**CCS Category 184**	Intraoperative cholangiogram	546 (0.1%)	238 (0.1%)
**CCS Category 185**	Upper gastrointestinal X-ray	17,268 (3.1%)	9,246 (3.0%)
**CCS Category 186**	Lower gastrointestinal X-ray	347 (0.1%)	169 (0.1%)
**CCS Category 187**	Intravenous pyelogram	993 (0.2%)	503 (0.2%)
**CCS Category 189**	Contrast aortogram	8,891 (1.6%)	4,425 (1.4%)
**CCS Category 190**	Contrast arteriogram of femoral and lower extremity arteries	3,369 (0.6%)	1,763 (0.6%)
**CCS Category 191**	Arterio- or venogram (not heart and head)	32,437 (5.9%)	18,470 (5.9%)
**CCS Category 192**	Diagnostic ultrasound of head and neck	23,725 (4.3%)	11,431 (3.7%)
**CCS Category 193**	Diagnostic ultrasound of heart (echocardiogram)	183,545 (33.5%)	98,376 (31.5%)
**CCS Category 194**	Diagnostic ultrasound of gastrointestinal tract	3,063 (0.6%)	1,096 (0.4%)
**CCS Category 195**	Diagnostic ultrasound of urinary tract	8,693 (1.6%)	2,880 (0.9%)
**CCS Category 196**	Diagnostic ultrasound of abdomen or retroperitoneum	85,403 (15.6%)	43,604 (14.0%)
**CCS Category 197**	Other diagnostic ultrasound	120,400 (22.0%)	64,885 (20.8%)
**CCS Category 198**	Magnetic resonance imaging	82,539 (15.1%)	45,530 (14.6%)
**CCS Category 199**	Electroencephalogram (EEG)	12,209 (2.2%)	6,035 (1.9%)
**CCS Category 200**	Nonoperative urinary system measurements	115,819 (21.1%)	62,762 (20.1%)
**CCS Category 201**	Cardiac stress tests	25,408 (4.6%)	11,796 (3.8%)
**CCS Category 202**	Electrocardiogram	364,756 (66.5%)	212,430 (68.0%)
**CCS Category 203**	Electrographic cardiac monitoring	52,772 (9.6%)	24,209 (7.7%)
**CCS Category 204**	Swan-Ganz catheterization for monitoring	4,968 (0.9%)	2,386 (0.8%)
**CCS Category 205**	Arterial blood gases	95,100 (17.3%)	51,222 (16.4%)
**CCS Category 206**	Microscopic examination (bacterial smear, culture, toxicology)	355,956 (64.9%)	192,674 (61.7%)
**CCS Category 207**	Radioisotope bone scan	2,833 (0.5%)	1,152 (0.4%)
**CCS Category 208**	Radioisotope pulmonary scan	5,506 (1.0%)	2,322 (0.7%)
**CCS Category 209**	Radioisotope scan and function studies	41,662 (7.6%)	20,500 (6.6%)
**CCS Category 210**	Other radioisotope scan	13,164 (2.4%)	4,715 (1.5%)

© 2025 Goto M et al. JAMA Network Open.

**CCS Category 211**	Therapeutic radiology	3,474 (0.6%)	1,568 (0.5%)
**CCS Category 212**	Diagnostic physical, occupational, and speech therapy	265,407 (48.4%)	153,178 (49.0%)
**CCS Category 213**	Physical, occupational, and speech therapy exercises; manipulation; and other procedures	208,021 (37.9%)	117,633 (37.6%)
**CCS Category 214**	Traction, splints, and other wound care	20,055 (3.7%)	9,110 (2.9%)
**CCS Category 215**	Other physical, occupational, and speech therapy and rehabilitation	67,902 (12.4%)	34,665 (11.1%)
**CCS Category 216**	Respiratory intubation and mechanical ventilation	58,565 (10.7%)	27,983 (9.0%)
**CCS Category 217**	Other respiratory therapy	42,472 (7.7%)	24,099 (7.7%)
**CCS Category 218**	Psychological and psychiatric evaluation and therapy	184,118 (33.6%)	110,482 (35.4%)
**CCS Category 219**	Alcohol and drug management, treatment, and rehabilitation	32,582 (5.9%)	19,275 (6.2%)
**CCS Category 220**	Ophthalmologic and otologic diagnosis and treatment	20,628 (3.8%)	11,377 (3.6%)
**CCS Category 221**	Nasogastric tube	1,588 (0.3%)	730 (0.2%)
**CCS Category 222**	Blood and blood product transfusion	48,803 (8.9%)	23,847 (7.6%)
**CCS Category 223**	Enteral and parenteral nutrition	2,989 (0.5%)	1,615 (0.5%)
**CCS Category 224**	Cancer chemotherapy	6,513 (1.2%)	3,270 (1.0%)
**CCS Category 225**	Conversion of cardiac rhythm	10,818 (2.0%)	5,561 (1.8%)
**CCS Category 226**	Other diagnostic radiology and related techniques	205,031 (37.4%)	110,695 (35.4%)
**CCS Category 227**	Consultation, evaluation, and preventative care	532,285 (97.1%)	302,490 (96.8%)
**CCS Category 228**	Prophylactic vaccinations and inoculations	67,307 (12.3%)	21,482 (6.9%)
**CCS Category 229**	Nonoperative removal of foreign body	1,387 (0.3%)	748 (0.2%)
**CCS Category 231**	Other therapeutic procedures	103,890 (19.0%)	58,443 (18.7%)
**CCS Category 232**	Anesthesia	144,413 (26.3%)	73,941 (23.7%)
**CCS Category 233**	Laboratory - Chemistry and Hematology	481,420 (87.8%)	272,352 (87.2%)
**CCS Category 234**	Pathology	154,432 (28.2%)	79,677 (25.5%)
**CCS Category 235**	Other Laboratory	351,024 (64.0%)	180,544 (57.8%)
**CCS Category 236**	Nonhospital-based care (e.g., home health care, hospice)	334,787 (61.1%)	194,341 (62.2%)
**CCS Category 237**	Ancillary Services	403,733 (73.6%)	226,693 (72.5%)
**CCS Category 239**	Transportation - patient, provider, equipment	3,708 (0.7%)	1,343 (0.4%)
**CCS Category 240**	Medications (Injections, infusions and other forms)	126,553 (23.1%)	67,174 (21.5%)
**CCS Category 241**	Visual aids and other optical supplies	1,233 (0.2%)	672 (0.2%)
**CCS Category 242**	Hearing devices and audiology supplies	3,558 (0.6%)	2,278 (0.7%)
**CCS Category 243**	DME and supplies	148,485 (27.1%)	80,063 (25.6%)
**CCS Category 244**	Gastric bypass and volume reduction	1,515 (0.3%)	1,015 (0.3%)
**CCS Category 245**	Telehealth (includes telephone calls, online communication, remote monitoring and surveillance)	349,245 (63.7%)	178,751 (57.2%)

© 2025 Goto M et al. JAMA Network Open.

*If a patient had multiple hospital admissions during the study period, evaluated for the first admission

Abbreviations: AIDS: Acquired Immunodeficiency Syndrome; DME: Durable Medical Equipment; GI: Gastrointestinal; HIV: Human Immunodeficiency Virus; OR: Operating Room

© 2025 Goto M et al. JAMA Network Open.

Supplementary Table 3. Zero-Inflated Negative Binomial Regression Model to Predict Days of Therapy (DOT) for Risk-Adjustment with Patient-Level Factors (Method 2)

**Count Component**			
**Variables**	**Descriptions**	**Rate Ratios (95%CIs)**	**p-values**
**Age**	<45	(Reference)	<0.001
	45-54	0.994 (0.983-1.006)	
	55-64	0.966 (0.957-0.976)	
	65-74	0.943 (0.934-0.953)	
	85-84	0.932 (0.923-0.942)	
	>=85	0.912 (0.901-0.922)	
**Male Gender**		1.045 (1.037-1.053)	<0.001
**Admission to ICU**		1.156 (1.150-1.162)	<0.001
**Month of Admission**	January	(Reference)	<0.001
	February	1.002 (0.993-1.011)	
	March	1.003 (0.995-1.012)	
	April	0.999 (0.990-1.008)	
	May	1.011 (1.003-1.020)	
	June	1.015 (1.006-1.024)	
	July	1.014 (1.005-1.023)	
	August	1.006 (0.997-1.014)	
	September	1.009 (1.000-1.018)	
	October	1.014 (1.006-1.023)	
	November	1.016 (1.007-1.025)	
	December	1.007 (0.998-1.016)	
**HCC Category 2**	Septicemia, Sepsis, Systemic Inflammatory Response Syndrome/Shock	1.191 (1.186-1.196)	<0.001
**HCC Category 6**	Opportunistic Infections	1.049 (1.038-1.060)	<0.001
**HCC Category 12**	Breast, Prostate, and Other Cancers and Tumors	0.985 (0.980-0.990)	<0.001
**HCC Category 21**	Protein-Calorie Malnutrition	0.972 (0.967-0.976)	<0.001
**HCC Category 23**	Other Significant Endocrine and Metabolic Disorders	0.970 (0.964-0.976)	<0.001
**HCC Category 27**	End-Stage Liver Disease	0.929 (0.921-0.937)	<0.001
**HCC Category 34**	Chronic Pancreatitis	0.978 (0.966-0.991)	<0.001
**HCC Category 35**	Inflammatory Bowel Disease	1.068 (1.056-1.079)	<0.001
**HCC Category 39**	Bone/Joint/Muscle Infections/Necrosis	1.205 (1.198-1.213)	<0.001

© 2025 Goto M et al. JAMA Network Open.

**HCC Category 47**	Disorders of Immunity	1.045 (1.038-1.052)	<0.001
**HCC Category 51**	Dementia With Complications	0.887 (0.872-0.903)	<0.001
**HCC Category 52**	Dementia Without Complication	0.922 (0.917-0.928)	<0.001
**HCC Category 55**	Drug/Alcohol Dependence	0.973 (0.968-0.978)	<0.001
**HCC Category 79**	Seizure Disorders and Convulsions	0.959 (0.951-0.966)	<0.001
**HCC Category 80**	Coma, Brain Compression/Anoxic Damage	0.921 (0.904-0.937)	<0.001
**HCC Category 82**	Respirator Dependence/Tracheostomy Status	0.948 (0.938-0.958)	<0.001
**HCC Category 85**	Congestive Heart Failure	0.977 (0.973-0.981)	<0.001
**HCC Category 87**	Unstable Angina and Other Acute Ischemic Heart Disease	0.976 (0.968-0.984)	<0.001
**HCC Category 96**	Specified Heart Arrhythmias	0.993 (0.988-0.997)	<0.001
**HCC Category 99**	Cerebral Hemorrhage	0.982 (0.968-0.997)	0.016
**HCC Category 106**	Atherosclerosis of the Extremities with Ulceration or Gangrene	1.063 (1.054-1.072)	<0.001
**HCC Category 112**	Fibrosis of Lung and Other Chronic Lung Disorders	1.020 (1.013-1.028)	<0.001
**HCC Category 114**	Aspiration and Specified Bacterial Pneumonias	1.075 (1.069-1.081)	<0.001
**HCC Category 115**	Pneumococcal Pneumonia, Empyema, Lung Abscess	1.131 (1.122-1.139)	<0.001
**HCC Category 135**	Acute Renal Failure	0.969 (0.965-0.973)	<0.001
**HCC Category 136**	Chronic Kidney Disease, Stage 5	0.873 (0.865-0.881)	<0.001
**HCC Category 137**	Chronic Kidney Disease, Severe (Stage 4)	0.964 (0.957-0.972)	<0.001
**HCC Category 138**	Chronic Kidney Disease, Moderate (Stage 3)	0.986 (0.981-0.991)	<0.001
**HCC Category 161**	Chronic Ulcer of Skin, Except Pressure	1.064 (1.057-1.070)	<0.001
**HCC Category 170**	Hip Fracture/Dislocation	0.937 (0.924-0.951)	<0.001
**HCC Category 176**	Complications of Specified Implanted Device or Graft	1.030 (1.024-1.036)	<0.001
**HCC Category 189**	Amputation Status, Lower Limb/Amputation Complications	0.963 (0.956-0.971)	<0.001
**CCS Category 3**	Laminectomy, excision intervertebral disc	0.693 (0.674-0.713)	<0.001
**CCS Category 4**	Diagnostic spinal tap	1.101 (1.076-1.126)	<0.001
**CCS Category 5**	Insertion of catheter or spinal stimulator and injection into spinal canal	0.860 (0.843-0.877)	<0.001
**CCS Category 8**	Other non-OR or closed therapeutic nervous system procedures	0.940 (0.928-0.951)	<0.001
**CCS Category 12**	Other therapeutic endocrine procedures	0.708 (0.651-0.771)	<0.001
**CCS Category 26**	Other therapeutic ear procedures	1.041 (1.005-1.079)	0.026
**CCS Category 31**	Diagnostic procedures on nose, mouth and pharynx	1.055 (1.048-1.062)	<0.001
**CCS Category 32**	Other non-OR therapeutic procedures on nose, mouth and pharynx	0.958 (0.950-0.966)	<0.001
**CCS Category 36**	Lobectomy or pneumonectomy	0.593 (0.570-0.617)	<0.001
**CCS Category 37**	Diagnostic bronchoscopy and biopsy of bronchus	1.083 (1.068-1.098)	<0.001
**CCS Category 38**	Other diagnostic procedures on lung and bronchus	0.967 (0.957-0.978)	<0.001

© 2025 Goto M et al. JAMA Network Open.

**CCS Category 41**	Other non-OR therapeutic procedures on respiratory system	1.017 (1.011-1.023)	<0.001
**CCS Category 43**	Heart valve procedures	0.826 (0.801-0.851)	<0.001
**CCS Category 44**	Coronary artery bypass graft (CABG)	0.616 (0.601-0.632)	<0.001
**CCS Category 47**	Diagnostic cardiac catheterization, coronary arteriography	0.878 (0.865-0.891)	<0.001
**CCS Category 48**	Insertion, revision, replacement, removal of cardiac pacemaker or cardioverter/defibrillator	0.740 (0.726-0.755)	<0.001
**CCS Category 49**	Other OR heart procedures	0.907 (0.879-0.936)	<0.001
**CCS Category 51**	Endarterectomy, vessel of head and neck	0.674 (0.639-0.712)	<0.001
**CCS Category 52**	Aortic resection, replacement or anastomosis	0.733 (0.702-0.766)	<0.001
**CCS Category 54**	Other vascular catheterization, not heart	1.061 (1.056-1.066)	<0.001
**CCS Category 55**	Peripheral vascular bypass	0.888 (0.867-0.910)	<0.001
**CCS Category 57**	Creation, revision and removal of arteriovenous fistula or vessel-to-vessel cannula for dialysis	0.824 (0.794-0.855)	<0.001
**CCS Category 59**	Other OR procedures on vessels of head and neck	0.842 (0.802-0.885)	<0.001
**CCS Category 62**	Other diagnostic cardiovascular procedures	1.065 (1.056-1.075)	<0.001
**CCS Category 67**	Other therapeutic procedures, hemic and lymphatic system	0.895 (0.875-0.916)	<0.001
**CCS Category 70**	Upper gastrointestinal endoscopy, biopsy	0.895 (0.885-0.904)	<0.001
**CCS Category 71**	Gastrostomy, temporary and permanent	0.841 (0.825-0.857)	<0.001
**CCS Category 78**	Colorectal resection	0.873 (0.854-0.891)	<0.001
**CCS Category 85**	Inguinal and femoral hernia repair	0.856 (0.806-0.909)	<0.001
**CCS Category 86**	Other hernia repair	0.873 (0.842-0.905)	<0.001
**CCS Category 89**	Exploratory laparotomy	1.064 (1.022-1.107)	0.002
**CCS Category 91**	Peritoneal dialysis	0.934 (0.922-0.946)	<0.001
**CCS Category 96**	Other OR lower GI therapeutic procedures	1.087 (1.058-1.118)	<0.001
**CCS Category 98**	Other non-OR gastrointestinal therapeutic procedures	1.181 (1.167-1.196)	<0.001
**CCS Category 100**	Endoscopy and endoscopic biopsy of the urinary tract	0.908 (0.884-0.933)	<0.001
**CCS Category 101**	Transurethral excision, drainage, or removal urinary obstruction	0.823 (0.801-0.847)	<0.001
**CCS Category 102**	Ureteral catheterization	0.900 (0.883-0.918)	<0.001
**CCS Category 104**	Nephrectomy, partial or complete	0.681 (0.653-0.710)	<0.001
**CCS Category 108**	Indwelling catheter	0.942 (0.931-0.954)	<0.001
**CCS Category 110**	Other diagnostic procedures of urinary tract	0.794 (0.760-0.829)	<0.001
**CCS Category 114**	Open prostatectomy	0.679 (0.650-0.708)	<0.001
**CCS Category 118**	Other OR therapeutic procedures, male genital	1.197 (1.161-1.234)	<0.001
**CCS Category 146**	Treatment, fracture or dislocation of hip and femur	0.554 (0.536-0.572)	<0.001
**CCS Category 147**	Treatment, fracture or dislocation of lower extremity (other than hip or femur)	0.599 (0.569-0.630)	<0.001

© 2025 Goto M et al. JAMA Network Open.

**CCS Category 152**	Arthroplasty knee	0.854 (0.842-0.867)	<0.001
**CCS Category 153**	Hip replacement, total and partial	0.756 (0.742-0.770)	<0.001
**CCS Category 157**	Amputation of lower extremity	1.169 (1.155-1.183)	<0.001
**CCS Category 158**	Spinal fusion	0.637 (0.618-0.656)	<0.001
**CCS Category 162**	Other OR therapeutic procedures on joints	1.120 (1.092-1.148)	<0.001
**CCS Category 163**	Other non-OR therapeutic procedures on musculoskeletal system	1.097 (1.059-1.135)	<0.001
**CCS Category 164**	Other OR therapeutic procedures on musculoskeletal system	1.045 (1.002-1.090)	0.040
**CCS Category 168**	Incision and drainage, skin and subcutaneous tissue	1.245 (1.228-1.262)	<0.001
**CCS Category 169**	Debridement of wound, infection or burn	1.185 (1.172-1.198)	<0.001
**CCS Category 170**	Excision of skin lesion	1.041 (1.022-1.061)	<0.001
**CCS Category 177**	Computerized axial tomography (CT) scan head	0.959 (0.954-0.964)	<0.001
**CCS Category 178**	CT scan chest	1.081 (1.076-1.086)	<0.001
**CCS Category 179**	CT scan abdomen	1.085 (1.080-1.089)	<0.001
**CCS Category 180**	Other CT scan	1.066 (1.059-1.072)	<0.001
**CCS Category 183**	Routine chest X-ray	1.023 (1.019-1.028)	<0.001
**CCS Category 185**	Upper gastrointestinal X-ray	0.971 (0.960-0.983)	<0.001
**CCS Category 189**	Contrast aortogram	1.049 (1.033-1.065)	<0.001
**CCS Category 191**	Arterio- or venogram (not heart and head)	0.951 (0.940-0.962)	<0.001
**CCS Category 192**	Diagnostic ultrasound of head and neck	0.947 (0.935-0.960)	<0.001
**CCS Category 197**	Other diagnostic ultrasound	1.045 (1.041-1.050)	<0.001
**CCS Category 198**	Magnetic resonance imaging	1.037 (1.031-1.044)	<0.001
**CCS Category 201**	Cardiac stress tests	0.925 (0.908-0.943)	<0.001
**CCS Category 202**	Electrocardiogram	0.989 (0.985-0.994)	<0.001
**CCS Category 203**	Electrographic cardiac monitoring	0.987 (0.978-0.996)	0.006
**CCS Category 205**	Arterial blood gases	1.018 (1.013-1.024)	<0.001
**CCS Category 206**	Microscopic examination (bacterial smear, culture, toxicology)	1.112 (1.107-1.117)	<0.001
**CCS Category 207**	Radioisotope bone scan	1.042 (1.017-1.068)	<0.001
**CCS Category 211**	Therapeutic radiology	0.888 (0.865-0.912)	<0.001
**CCS Category 213**	Physical, occupational, and speech therapy exercises; manipulation; and other procedures	0.948 (0.944-0.951)	<0.001
**CCS Category 214**	Traction, splints, and other wound care	1.048 (1.040-1.057)	<0.001
**CCS Category 215**	Other physical, occupational, and speech therapy and rehabilitation	0.956 (0.950-0.962)	<0.001
**CCS Category 219**	Alcohol and drug management, treatment, and rehabilitation	0.951 (0.940-0.962)	<0.001
**CCS Category 225**	Conversion of cardiac rhythm	0.939 (0.917-0.960)	<0.001
**CCS Category 226**	Other diagnostic radiology and related techniques	1.031 (1.026-1.035)	<0.001

© 2025 Goto M et al. JAMA Network Open.

**CCS Category 228**	Prophylactic vaccinations and inoculations	0.962 (0.955-0.969)	<0.001
**CCS Category 232**	Anesthesia	0.964 (0.959-0.970)	<0.001
**CCS Category 233**	Laboratory - Chemistry and Hematology	0.978 (0.973-0.983)	<0.001
**CCS Category 234**	Pathology	0.978 (0.973-0.983)	<0.001
**CCS Category 235**	Other Laboratory	1.010 (1.006-1.015)	<0.001
**CCS Category 237**	Ancillary Services	1.054 (1.049-1.058)	<0.001
**CCS Category 243**	DME and supplies	0.990 (0.986-0.995)	<0.001
**CCS Category 244**	Gastric bypass and volume reduction	0.543 (0.472-0.625)	<0.001
**CCS Category 245**	Telehealth (includes telephone calls, online communication, remote monitoring and surveillance)	1.065 (1.061-1.070)	<0.001
**Zero-Inflation Component**			
**Variables**	**Descriptions**	**Odds Ratios* (95%CIs)**	**p-values**
**Age**	<45	(Reference)	<0.001
	45-54	0.951 (0.926-0.976)	
	55-64	0.916 (0.895-0.937)	
	65-74	0.860 (0.841-0.880)	
	85-84	0.837 (0.817-0.858)	
	>=85	0.769 (0.748-0.791)	
**Admission to ICU**		0.656 (0.646-0.666)	<0.001
**Admission to Surgical Specialties**		0.901 (0.889-0.912)	<0.001
**Month of Admission**	January	(Reference)	<0.001
	February	1.005 (0.983-1.029)	
	March	1.014 (0.992-1.037)	
	April	1.029 (1.006-1.053)	
	May	0.989 (0.967-1.011)	
	June	0.955 (0.934-0.977)	
	July	0.928 (0.907-0.949)	
	August	0.919 (0.899-0.940)	
	September	0.918 (0.898-0.939)	
	October	0.907 (0.887-0.928)	
	November	0.914 (0.893-0.935)	
	December	0.940 (0.919-0.961)	
**HCC Category 1**	HIV/AIDS	0.646 (0.620-0.674)	<0.001
**HCC Category 2**	Septicemia, Sepsis, Systemic Inflammatory Response Syndrome/Shock	0.285 (0.281-0.289)	<0.001

© 2025 Goto M et al. JAMA Network Open.

**HCC Category 6**	Opportunistic Infections	0.766 (0.736-0.797)	<0.001
**HCC Category 23**	Other Significant Endocrine and Metabolic Disorders	1.105 (1.087-1.123)	<0.001
**HCC Category 27**	End-Stage Liver Disease	0.708 (0.690-0.727)	<0.001
**HCC Category 34**	Chronic Pancreatitis	1.328 (1.286-1.371)	<0.001
**HCC Category 35**	Inflammatory Bowel Disease	0.861 (0.836-0.887)	<0.001
**HCC Category 39**	Bone/Joint/Muscle Infections/Necrosis	0.481 (0.471-0.492)	<0.001
**HCC Category 47**	Disorders of Immunity	0.784 (0.769-0.801)	<0.001
**HCC Category 52**	Dementia Without Complication	1.102 (1.085-1.119)	<0.001
**HCC Category 54**	Drug/Alcohol Psychosis	1.427 (1.384-1.472)	<0.001
**HCC Category 55**	Drug/Alcohol Dependence	1.146 (1.130-1.161)	<0.001
**HCC Category 56**	Major Depressive, Bipolar, and Paranoid Disorders	1.045 (1.024-1.067)	<0.001
**HCC Category 57**	Schizophrenia	1.121 (1.095-1.149)	<0.001
**HCC Category 59**	Personality Disorders	1.082 (1.070-1.093)	<0.001
**HCC Category 60**	Eating Disorders	1.155 (1.123-1.188)	<0.001
**HCC Category 71**	Paraplegia	0.762 (0.727-0.798)	<0.001
**HCC Category 75**	Myasthenia Gravis/Myoneural Disorders, Inflammatory and Toxic Neuropathy	1.176 (1.138-1.216)	<0.001
**HCC Category 79**	Seizure Disorders and Convulsions	1.132 (1.111-1.155)	<0.001
**HCC Category 82**	Respirator Dependence/Tracheostomy Status	1.258 (1.211-1.308)	<0.001
**HCC Category 84**	Cardio-Respiratory Failure and Shock	0.777 (0.767-0.787)	<0.001
**HCC Category 85**	Congestive Heart Failure	1.210 (1.196-1.224)	<0.001
**HCC Category 86**	Acute Myocardial Infarction	1.134 (1.116-1.153)	<0.001
**HCC Category 87**	Unstable Angina and Other Acute Ischemic Heart Disease	1.320 (1.292-1.348)	<0.001
**HCC Category 88**	Angina Pectoris	1.248 (1.228-1.269)	<0.001
**HCC Category 96**	Specified Heart Arrhythmias	1.102 (1.089-1.114)	<0.001
**HCC Category 100**	Ischemic or Unspecified Stroke	1.177 (1.159-1.197)	<0.001
**HCC Category 106**	Atherosclerosis of the Extremities with Ulceration or Gangrene	0.873 (0.848-0.899)	<0.001
**HCC Category 107**	Vascular Disease with Complications	1.168 (1.149-1.186)	<0.001
**HCC Category 111**	Chronic Obstructive Pulmonary Disease	0.800 (0.792-0.809)	<0.001
**HCC Category 112**	Fibrosis of Lung and Other Chronic Lung Disorders	0.877 (0.859-0.895)	<0.001
**HCC Category 114**	Aspiration and Specified Bacterial Pneumonias	0.509 (0.498-0.520)	<0.001
**HCC Category 115**	Pneumococcal Pneumonia, Empyema, Lung Abscess	0.505 (0.490-0.520)	<0.001
**HCC Category 136**	Chronic Kidney Disease, Stage 5	1.247 (1.219-1.276)	<0.001
**HCC Category 137**	Chronic Kidney Disease, Severe (Stage 4)	1.157 (1.133-1.181)	<0.001
**HCC Category 138**	Chronic Kidney Disease, Moderate (Stage 3)	1.084 (1.071-1.098)	<0.001

© 2025 Goto M et al. JAMA Network Open.

**HCC Category 161**	Chronic Ulcer of Skin, Except Pressure	0.698 (0.685-0.711)	<0.001
**HCC Category 169**	Vertebral Fractures without Spinal Cord Injury	1.203 (1.165-1.243)	<0.001
**HCC Category 170**	Hip Fracture/Dislocation	1.392 (1.337-1.450)	<0.001
**HCC Category 176**	Complications of Specified Implanted Device or Graft	0.585 (0.574-0.596)	<0.001
**HCC Category 186**	Major Organ Transplant or Replacement Status	0.554 (0.528-0.582)	<0.001
**CCS Category 3**	Laminectomy, excision intervertebral disc	0.177 (0.159-0.196)	<0.001
**CCS Category 4**	Diagnostic spinal tap	0.651 (0.605-0.700)	<0.001
**CCS Category 9**	Other OR therapeutic nervous system procedures	0.475 (0.419-0.539)	<0.001
**CCS Category 15**	Lens and cataract procedures	3.795 (3.096-4.650)	<0.001
**CCS Category 26**	Other therapeutic ear procedures	0.630 (0.564-0.704)	<0.001
**CCS Category 28**	Plastic procedures on nose	0.252 (0.195-0.325)	<0.001
**CCS Category 29**	Oral and Dental Services	0.118 (0.098-0.141)	<0.001
**CCS Category 31**	Diagnostic procedures on nose, mouth and pharynx	0.647 (0.635-0.659)	<0.001
**CCS Category 33**	Other OR therapeutic procedures on nose, mouth and pharynx	0.320 (0.287-0.356)	<0.001
**CCS Category 37**	Diagnostic bronchoscopy and biopsy of bronchus	0.784 (0.743-0.827)	<0.001
**CCS Category 39**	Incision of pleura, thoracentesis, chest drainage	1.212 (1.163-1.262)	<0.001
**CCS Category 41**	Other non-OR therapeutic procedures on respiratory system	0.688 (0.675-0.700)	<0.001
**CCS Category 43**	Heart valve procedures	0.323 (0.288-0.363)	<0.001
**CCS Category 44**	Coronary artery bypass graft (CABG)	0.137 (0.124-0.152)	<0.001
**CCS Category 45**	Percutaneous transluminal coronary angioplasty (PTCA)	1.627 (1.521-1.740)	<0.001
**CCS Category 47**	Diagnostic cardiac catheterization, coronary arteriography	2.300 (2.218-2.384)	<0.001
**CCS Category 48**	Insertion, revision, replacement, removal of cardiac pacemaker or cardioverter/defibrillator	0.082 (0.076-0.089)	<0.001
**CCS Category 54**	Other vascular catheterization, not heart	0.848 (0.836-0.860)	<0.001
**CCS Category 57**	Creation, revision and removal of arteriovenous fistula or vessel-to-vessel cannula for dialysis	2.596 (2.377-2.836)	<0.001
**CCS Category 62**	Other diagnostic cardiovascular procedures	0.793 (0.768-0.818)	<0.001
**CCS Category 68**	Injection or ligation of esophageal varices	0.084 (0.064-0.111)	<0.001
**CCS Category 70**	Upper gastrointestinal endoscopy, biopsy	1.397 (1.358-1.437)	<0.001
**CCS Category 76**	Colonoscopy and biopsy	1.538 (1.473-1.605)	<0.001
**CCS Category 80**	Appendectomy	0.068 (0.055-0.083)	<0.001
**CCS Category 82**	Endoscopic retrograde cannulation of pancreas (ERCP)	0.452 (0.414-0.494)	<0.001
**CCS Category 84**	Cholecystectomy and common duct exploration	0.270 (0.252-0.289)	<0.001
**CCS Category 85**	Inguinal and femoral hernia repair	2.915 (2.586-3.286)	<0.001
**CCS Category 86**	Other hernia repair	1.815 (1.682-1.958)	<0.001

© 2025 Goto M et al. JAMA Network Open.

**CCS Category 88**	Abdominal paracentesis	0.534 (0.508-0.561)	<0.001
**CCS Category 92**	Other bowel diagnostic procedures	1.292 (1.220-1.368)	<0.001
**CCS Category 96**	Other OR lower GI therapeutic procedures	0.649 (0.598-0.704)	<0.001
**CCS Category 98**	Other non-OR gastrointestinal therapeutic procedures	0.564 (0.538-0.591)	<0.001
**CCS Category 100**	Endoscopy and endoscopic biopsy of the urinary tract	0.565 (0.513-0.623)	<0.001
**CCS Category 101**	Transurethral excision, drainage, or removal urinary obstruction	0.521 (0.479-0.568)	<0.001
**CCS Category 102**	Ureteral catheterization	0.472 (0.439-0.507)	<0.001
**CCS Category 103**	Nephrotomy and nephrostomy	0.580 (0.526-0.639)	<0.001
**CCS Category 104**	Nephrectomy, partial or complete	0.340 (0.306-0.379)	<0.001
**CCS Category 107**	Extracorporeal lithotripsy, urinary	0.404 (0.360-0.452)	<0.001
**CCS Category 108**	Indwelling catheter	0.586 (0.560-0.613)	<0.001
**CCS Category 109**	Procedures on the urethra	0.236 (0.191-0.290)	<0.001
**CCS Category 111**	Other non-OR therapeutic procedures of urinary tract	0.674 (0.627-0.724)	<0.001
**CCS Category 112**	Other OR therapeutic procedures of urinary tract	0.473 (0.429-0.522)	<0.001
**CCS Category 114**	Open prostatectomy	0.160 (0.140-0.183)	<0.001
**CCS Category 116**	Diagnostic procedures, male genital	0.289 (0.226-0.370)	<0.001
**CCS Category 118**	Other OR therapeutic procedures, male genital	0.094 (0.082-0.107)	<0.001
**CCS Category 142**	Partial excision bone	0.233 (0.189-0.287)	<0.001
**CCS Category 146**	Treatment, fracture or dislocation of hip and femur	0.061 (0.050-0.075)	<0.001
**CCS Category 147**	Treatment, fracture or dislocation of lower extremity (other than hip or femur)	0.130 (0.105-0.162)	<0.001
**CCS Category 152**	Arthroplasty knee	0.002 (0.001-0.003)	<0.001
**CCS Category 153**	Hip replacement, total and partial	0.004 (0.003-0.006)	<0.001
**CCS Category 154**	Arthroplasty other than hip or knee	0.015 (0.011-0.021)	<0.001
**CCS Category 157**	Amputation of lower extremity	0.268 (0.250-0.287)	<0.001
**CCS Category 158**	Spinal fusion	0.184 (0.158-0.215)	<0.001
**CCS Category 159**	Other diagnostic procedures on musculoskeletal system	0.700 (0.653-0.750)	<0.001
**CCS Category 160**	Other therapeutic procedures on muscles and tendons	0.252 (0.223-0.285)	<0.001
**CCS Category 161**	Other OR therapeutic procedures on bone	0.585 (0.518-0.662)	<0.001
**CCS Category 162**	Other OR therapeutic procedures on joints	0.118 (0.093-0.151)	<0.001
**CCS Category 163**	Other non-OR therapeutic procedures on musculoskeletal system	0.384 (0.331-0.446)	<0.001
**CCS Category 164**	Other OR therapeutic procedures on musculoskeletal system	0.205 (0.153-0.274)	<0.001
**CCS Category 168**	Incision and drainage, skin and subcutaneous tissue	0.049 (0.042-0.056)	<0.001
**CCS Category 169**	Debridement of wound, infection or burn	0.285 (0.266-0.306)	<0.001
**CCS Category 170**	Excision of skin lesion	0.789 (0.738-0.844)	<0.001

© 2025 Goto M et al. JAMA Network Open.

**CCS Category 172**	Skin graft	0.423 (0.379-0.471)	<0.001
**CCS Category 173**	Other diagnostic procedures on skin and subcutaneous tissue	0.752 (0.704-0.804)	<0.001
**CCS Category 175**	Other OR therapeutic procedures on skin and breast	0.237 (0.210-0.266)	<0.001
**CCS Category 177**	Computerized axial tomography (CT) scan head	1.261 (1.244-1.280)	<0.001
**CCS Category 178**	CT scan chest	0.798 (0.788-0.809)	<0.001
**CCS Category 179**	CT scan abdomen	0.579 (0.572-0.587)	<0.001
**CCS Category 180**	Other CT scan	0.894 (0.877-0.911)	<0.001
**CCS Category 183**	Routine chest X-ray	0.784 (0.775-0.793)	<0.001
**CCS Category 191**	Arterio- or venogram (not heart and head)	1.594 (1.547-1.642)	<0.001
**CCS Category 192**	Diagnostic ultrasound of head and neck	1.500 (1.450-1.552)	<0.001
**CCS Category 196**	Diagnostic ultrasound of abdomen or retroperitoneum	0.741 (0.729-0.753)	<0.001
**CCS Category 197**	Other diagnostic ultrasound	0.690 (0.680-0.700)	<0.001
**CCS Category 198**	Magnetic resonance imaging	1.116 (1.096-1.136)	<0.001
**CCS Category 200**	Nonoperative urinary system measurements	0.881 (0.868-0.895)	<0.001
**CCS Category 201**	Cardiac stress tests	2.152 (2.072-2.236)	<0.001
**CCS Category 202**	Electrocardiogram	1.242 (1.228-1.256)	<0.001
**CCS Category 203**	Electrographic cardiac monitoring	1.263 (1.235-1.292)	<0.001
**CCS Category 204**	Swan-Ganz catheterization for monitoring	0.752 (0.690-0.819)	<0.001
**CCS Category 205**	Arterial blood gases	0.771 (0.759-0.783)	<0.001
**CCS Category 206**	Microscopic examination (bacterial smear, culture, toxicology)	0.503 (0.497-0.509)	<0.001
**CCS Category 211**	Therapeutic radiology	1.531 (1.431-1.639)	<0.001
**CCS Category 212**	Diagnostic physical, occupational, and speech therapy	0.879 (0.870-0.889)	<0.001
**CCS Category 214**	Traction, splints, and other wound care	0.631 (0.611-0.652)	<0.001
**CCS Category 215**	Other physical, occupational, and speech therapy and rehabilitation	1.104 (1.083-1.124)	<0.001
**CCS Category 216**	Respiratory intubation and mechanical ventilation	0.893 (0.875-0.910)	<0.001
**CCS Category 217**	Other respiratory therapy	0.570 (0.557-0.583)	<0.001
**CCS Category 218**	Psychological and psychiatric evaluation and therapy	1.137 (1.123-1.152)	<0.001
**CCS Category 219**	Alcohol and drug management, treatment, and rehabilitation	1.676 (1.633-1.720)	<0.001
**CCS Category 222**	Blood and blood product transfusion	0.946 (0.927-0.966)	<0.001
**CCS Category 225**	Conversion of cardiac rhythm	2.224 (2.106-2.349)	<0.001
**CCS Category 226**	Other diagnostic radiology and related techniques	0.778 (0.769-0.787)	<0.001
**CCS Category 227**	Consultation, evaluation, and preventative care	0.886 (0.868-0.905)	<0.001
**CCS Category 228**	Prophylactic vaccinations and inoculations	1.052 (1.031-1.072)	<0.001
**CCS Category 233**	Laboratory - Chemistry and Hematology	1.544 (1.523-1.566)	<0.001

© 2025 Goto M et al. JAMA Network Open.

**CCS Category 236**	Nonhospital-based care (e.g., home health care, hospice)	0.963 (0.953-0.973)	<0.001
**CCS Category 237**	Ancillary Services	0.849 (0.839-0.858)	<0.001
**CCS Category 244**	Gastric bypass and volume reduction	2.202 (1.831-2.649)	<0.001
**CCS Category 245**	Telehealth (includes telephone calls, online communication, remote monitoring and surveillance)	0.813 (0.805-0.822)	<0.001
**Log(Days Present)**		0.905 (0.898-0.913)	<0.001

*Odds ratio for having no antibiotic usage

Abbreviations: AIDS: Acquired Immunodeficiency Syndrome; CCS: Clinical Classification Software; CI: Confidence Interval; CT: Computed Tomography; DME: Durable Medical Equipment; GI: Gastrointestinal; HCC: Hierarchical Condition Categories; HIV: Human Immunodeficiency Virus; ICU: Intensive Care Unit; OR: Operating Room; 95%CI: 95% confidence interval

© 2025 Goto M et al. JAMA Network Open.

Supplementary Table 4. Zero-Inflated Negative Binomial Regression Model to Predict Days of Antimicrobial Spectrum Coverage (DASC) for Risk-Adjustment with Patient-Level Factors (Method 2)

**Count Component**			
**Variables**	**Descriptions**	**Rate Ratios (95%CIs)**	**p-values**
**Age**	<45	(Reference)	<0.001
	45-54	0.994 (0.984-1.004)	
	55-64	0.968 (0.960-0.976)	
	65-74	0.949 (0.941-0.957)	
	85-84	0.942 (0.933-0.950)	
	>=85	0.921 (0.912-0.931)	
**Male Gender**		1.048 (1.040-1.055)	<0.001
**Admission to ICU**		1.147 (1.142-1.153)	<0.001
**Admission to Surgical Specialties**		0.990 (0.986-0.995)	<0.001
**Month of Admission**	January	(Reference)	<0.001
	February	1.009 (1.001-1.017)	
	March	1.011 (1.003-1.019)	
	April	1.010 (1.002-1.018)	
	May	1.012 (1.004-1.020)	
	June	1.019 (1.012-1.027)	
	July	1.018 (1.010-1.026)	
	August	1.018 (1.010-1.025)	
	September	1.016 (1.008-1.024)	
	October	1.020 (1.012-1.028)	
	November	1.025 (1.017-1.033)	
	December	1.014 (1.007-1.022)	
**HCC Category 1**	HIV/AIDS	1.032 (1.018-1.046)	<0.001
**HCC Category 2**	Septicemia, Sepsis, Systemic Inflammatory Response Syndrome/Shock	1.199 (1.194-1.203)	<0.001
**HCC Category 6**	Opportunistic Infections	1.050 (1.039-1.060)	<0.001
**HCC Category 8**	Metastatic Cancer and Acute Leukemia	1.021 (1.014-1.028)	<0.001
**HCC Category 9**	Lung and Other Severe Cancers	1.028 (1.022-1.034)	<0.001
**HCC Category 10**	Lymphoma and Other Cancers	1.023 (1.015-1.031)	<0.001
**HCC Category 11**	Colorectal, Bladder, and Other Cancers	0.985 (0.979-0.991)	<0.001
**HCC Category 23**	Other Significant Endocrine and Metabolic Disorders	0.973 (0.968-0.978)	<0.001

© 2025 Goto M et al. JAMA Network Open.

**HCC Category 27**	End-Stage Liver Disease	0.949 (0.940-0.958)	<0.001
**HCC Category 28**	Cirrhosis of Liver	0.967 (0.960-0.974)	<0.001
**HCC Category 33**	Intestinal Obstruction/Perforation	1.040 (1.034-1.046)	<0.001
**HCC Category 35**	Inflammatory Bowel Disease	1.025 (1.015-1.035)	<0.001
**HCC Category 39**	Bone/Joint/Muscle Infections/Necrosis	1.177 (1.171-1.184)	<0.001
**HCC Category 47**	Disorders of Immunity	1.065 (1.058-1.072)	<0.001
**HCC Category 51**	Dementia With Complications	0.918 (0.903-0.933)	<0.001
**HCC Category 52**	Dementia Without Complication	0.934 (0.929-0.939)	<0.001
**HCC Category 55**	Drug/Alcohol Dependence	0.974 (0.969-0.978)	<0.001
**HCC Category 57**	Schizophrenia	0.974 (0.966-0.983)	<0.001
**HCC Category 79**	Seizure Disorders and Convulsions	0.966 (0.959-0.973)	<0.001
**HCC Category 80**	Coma, Brain Compression/Anoxic Damage	0.934 (0.918-0.951)	<0.001
**HCC Category 84**	Cardio-Respiratory Failure and Shock	1.019 (1.015-1.024)	<0.001
**HCC Category 85**	Congestive Heart Failure	0.970 (0.966-0.973)	<0.001
**HCC Category 106**	Atherosclerosis of the Extremities with Ulceration or Gangrene	1.080 (1.071-1.088)	<0.001
**HCC Category 107**	Vascular Disease with Complications	0.988 (0.983-0.994)	<0.001
**HCC Category 112**	Fibrosis of Lung and Other Chronic Lung Disorders	1.031 (1.025-1.038)	<0.001
**HCC Category 114**	Aspiration and Specified Bacterial Pneumonias	1.128 (1.122-1.134)	<0.001
**HCC Category 115**	Pneumococcal Pneumonia, Empyema, Lung Abscess	1.123 (1.115-1.131)	<0.001
**HCC Category 135**	Acute Renal Failure	0.976 (0.972-0.980)	<0.001
**HCC Category 136**	Chronic Kidney Disease, Stage 5	0.888 (0.881-0.895)	<0.001
**HCC Category 138**	Chronic Kidney Disease, Moderate (Stage 3)	0.980 (0.976-0.984)	<0.001
**HCC Category 159**	Major Skin Burn or Condition	0.974 (0.968-0.981)	<0.001
**HCC Category 161**	Chronic Ulcer of Skin, Except Pressure	1.071 (1.064-1.077)	<0.001
**HCC Category 170**	Hip Fracture/Dislocation	0.951 (0.938-0.963)	<0.001
**HCC Category 176**	Complications of Specified Implanted Device or Graft	1.063 (1.058-1.069)	<0.001
**HCC Category 186**	Major Organ Transplant or Replacement Status	1.092 (1.077-1.108)	<0.001
**CCS Category 1**	Incision and excision of CNS	0.575 (0.547-0.604)	<0.001
**CCS Category 3**	Laminectomy, excision intervertebral disc	0.554 (0.542-0.566)	<0.001
**CCS Category 5**	Insertion of catheter or spinal stimulator and injection into spinal canal	0.862 (0.847-0.877)	<0.001
**CCS Category 8**	Other non-OR or closed therapeutic nervous system procedures	0.922 (0.911-0.932)	<0.001
**CCS Category 10**	Thyroidectomy, partial or complete	0.643 (0.602-0.686)	<0.001
**CCS Category 12**	Other therapeutic endocrine procedures	0.660 (0.617-0.707)	<0.001
**CCS Category 28**	Plastic procedures on nose	0.670 (0.631-0.711)	<0.001

© 2025 Goto M et al. JAMA Network Open.

**CCS Category 29**	Oral and Dental Services	1.083 (1.043-1.126)	<0.001
**CCS Category 31**	Diagnostic procedures on nose, mouth and pharynx	1.067 (1.060-1.074)	<0.001
**CCS Category 32**	Other non-OR therapeutic procedures on nose, mouth and pharynx	0.960 (0.952-0.968)	<0.001
**CCS Category 36**	Lobectomy or pneumonectomy	0.485 (0.468-0.501)	<0.001
**CCS Category 37**	Diagnostic bronchoscopy and biopsy of bronchus	1.110 (1.095-1.126)	<0.001
**CCS Category 38**	Other diagnostic procedures on lung and bronchus	0.977 (0.968-0.987)	<0.001
**CCS Category 39**	Incision of pleura, thoracentesis, chest drainage	1.034 (1.021-1.047)	<0.001
**CCS Category 41**	Other non-OR therapeutic procedures on respiratory system	1.015 (1.009-1.020)	<0.001
**CCS Category 43**	Heart valve procedures	0.750 (0.731-0.770)	<0.001
**CCS Category 44**	Coronary artery bypass graft (CABG)	0.480 (0.469-0.491)	<0.001
**CCS Category 47**	Diagnostic cardiac catheterization, coronary arteriography	0.899 (0.887-0.912)	<0.001
**CCS Category 48**	Insertion, revision, replacement, removal of cardiac pacemaker or cardioverter/defibrillator	0.602 (0.592-0.611)	<0.001
**CCS Category 49**	Other OR heart procedures	0.903 (0.878-0.930)	<0.001
**CCS Category 51**	Endarterectomy, vessel of head and neck	0.459 (0.440-0.479)	<0.001
**CCS Category 52**	Aortic resection, replacement or anastomosis	0.601 (0.579-0.624)	<0.001
**CCS Category 54**	Other vascular catheterization, not heart	1.040 (1.035-1.045)	<0.001
**CCS Category 55**	Peripheral vascular bypass	0.813 (0.795-0.832)	<0.001
**CCS Category 57**	Creation, revision and removal of arteriovenous fistula or vessel-to-vessel cannula for dialysis	0.831 (0.803-0.860)	<0.001
**CCS Category 59**	Other OR procedures on vessels of head and neck	0.796 (0.762-0.832)	<0.001
**CCS Category 61**	Other OR procedures on vessels other than head and neck	0.940 (0.929-0.952)	<0.001
**CCS Category 62**	Other diagnostic cardiovascular procedures	1.056 (1.046-1.065)	<0.001
**CCS Category 67**	Other therapeutic procedures, hemic and lymphatic system	0.872 (0.854-0.891)	<0.001
**CCS Category 68**	Injection or ligation of esophageal varices	0.876 (0.840-0.913)	<0.001
**CCS Category 70**	Upper gastrointestinal endoscopy, biopsy	0.894 (0.885-0.903)	<0.001
**CCS Category 71**	Gastrostomy, temporary and permanent	0.852 (0.835-0.869)	<0.001
**CCS Category 78**	Colorectal resection	0.855 (0.838-0.873)	<0.001
**CCS Category 80**	Appendectomy	1.194 (1.164-1.224)	<0.001
**CCS Category 82**	Endoscopic retrograde cannulation of pancreas (ERCP)	1.135 (1.110-1.160)	<0.001
**CCS Category 84**	Cholecystectomy and common duct exploration	1.179 (1.158-1.200)	<0.001
**CCS Category 85**	Inguinal and femoral hernia repair	0.817 (0.777-0.858)	<0.001
**CCS Category 86**	Other hernia repair	0.823 (0.799-0.849)	<0.001
**CCS Category 88**	Abdominal paracentesis	1.017 (1.004-1.031)	0.013
**CCS Category 91**	Peritoneal dialysis	0.937 (0.926-0.948)	<0.001

© 2025 Goto M et al. JAMA Network Open.

**CCS Category 92**	Other bowel diagnostic procedures	0.934 (0.915-0.954)	<0.001
**CCS Category 96**	Other OR lower GI therapeutic procedures	1.123 (1.095-1.151)	<0.001
**CCS Category 98**	Other non-OR gastrointestinal therapeutic procedures	1.167 (1.152-1.183)	<0.001
**CCS Category 99**	Other OR gastrointestinal therapeutic procedures	1.092 (1.071-1.112)	<0.001
**CCS Category 101**	Transurethral excision, drainage, or removal urinary obstruction	0.889 (0.870-0.910)	<0.001
**CCS Category 102**	Ureteral catheterization	0.955 (0.938-0.971)	<0.001
**CCS Category 104**	Nephrectomy, partial or complete	0.572 (0.554-0.590)	<0.001
**CCS Category 108**	Indwelling catheter	0.955 (0.945-0.966)	<0.001
**CCS Category 110**	Other diagnostic procedures of urinary tract	0.882 (0.845-0.920)	<0.001
**CCS Category 114**	Open prostatectomy	0.562 (0.546-0.579)	<0.001
**CCS Category 118**	Other OR therapeutic procedures, male genital	1.264 (1.233-1.295)	<0.001
**CCS Category 124**	Hysterectomy, abdominal and vaginal	0.527 (0.478-0.582)	<0.001
**CCS Category 142**	Partial excision bone	1.085 (1.059-1.112)	<0.001
**CCS Category 146**	Treatment, fracture or dislocation of hip and femur	0.493 (0.480-0.507)	<0.001
**CCS Category 147**	Treatment, fracture or dislocation of lower extremity (other than hip or femur)	0.501 (0.482-0.522)	<0.001
**CCS Category 148**	Other fracture and dislocation procedure	0.693 (0.656-0.733)	<0.001
**CCS Category 152**	Arthroplasty knee	0.494 (0.488-0.501)	<0.001
**CCS Category 153**	Hip replacement, total and partial	0.467 (0.459-0.474)	<0.001
**CCS Category 154**	Arthroplasty other than hip or knee	0.565 (0.551-0.580)	<0.001
**CCS Category 157**	Amputation of lower extremity	1.200 (1.185-1.214)	<0.001
**CCS Category 158**	Spinal fusion	0.550 (0.538-0.563)	<0.001
**CCS Category 162**	Other OR therapeutic procedures on joints	1.091 (1.064-1.119)	<0.001
**CCS Category 168**	Incision and drainage, skin and subcutaneous tissue	1.237 (1.221-1.254)	<0.001
**CCS Category 169**	Debridement of wound, infection or burn	1.205 (1.192-1.219)	<0.001
**CCS Category 177**	Computerized axial tomography (CT) scan head	0.975 (0.970-0.979)	<0.001
**CCS Category 178**	CT scan chest	1.072 (1.067-1.077)	<0.001
**CCS Category 179**	CT scan abdomen	1.098 (1.094-1.102)	<0.001
**CCS Category 180**	Other CT scan	1.060 (1.054-1.067)	<0.001
**CCS Category 183**	Routine chest X-ray	1.022 (1.018-1.026)	<0.001
**CCS Category 185**	Upper gastrointestinal X-ray	0.966 (0.955-0.977)	<0.001
**CCS Category 189**	Contrast aortogram	1.072 (1.055-1.088)	<0.001
**CCS Category 191**	Arterio- or venogram (not heart and head)	0.970 (0.959-0.981)	<0.001
**CCS Category 192**	Diagnostic ultrasound of head and neck	0.951 (0.939-0.963)	<0.001
**CCS Category 197**	Other diagnostic ultrasound	1.026 (1.022-1.031)	<0.001

© 2025 Goto M et al. JAMA Network Open.

**CCS Category 202**	Electrocardiogram	0.986 (0.982-0.990)	<0.001
**CCS Category 205**	Arterial blood gases	1.007 (1.002-1.012)	0.009
**CCS Category 206**	Microscopic examination (bacterial smear, culture, toxicology)	1.098 (1.093-1.102)	<0.001
**CCS Category 207**	Radioisotope bone scan	1.042 (1.016-1.069)	0.001
**CCS Category 211**	Therapeutic radiology	0.905 (0.882-0.928)	<0.001
**CCS Category 212**	Diagnostic physical, occupational, and speech therapy	0.963 (0.959-0.968)	<0.001
**CCS Category 213**	Physical, occupational, and speech therapy exercises; manipulation; and other procedures	0.928 (0.924-0.933)	<0.001
**CCS Category 214**	Traction, splints, and other wound care	1.052 (1.043-1.061)	<0.001
**CCS Category 215**	Other physical, occupational, and speech therapy and rehabilitation	0.961 (0.956-0.967)	<0.001
**CCS Category 216**	Respiratory intubation and mechanical ventilation	1.024 (1.018-1.030)	<0.001
**CCS Category 218**	Psychological and psychiatric evaluation and therapy	0.958 (0.954-0.963)	<0.001
**CCS Category 219**	Alcohol and drug management, treatment, and rehabilitation	0.942 (0.932-0.951)	<0.001
**CCS Category 220**	Ophthalmologic and otologic diagnosis and treatment	0.975 (0.963-0.987)	<0.001
**CCS Category 228**	Prophylactic vaccinations and inoculations	0.955 (0.948-0.961)	<0.001
**CCS Category 232**	Anesthesia	0.949 (0.944-0.954)	<0.001
**CCS Category 233**	Laboratory - Chemistry and Hematology	0.980 (0.975-0.984)	<0.001
**CCS Category 234**	Pathology	0.966 (0.961-0.971)	<0.001
**CCS Category 236**	Nonhospital-based care (e.g., home health care, hospice)	0.969 (0.965-0.972)	<0.001
**CCS Category 243**	DME and supplies	0.980 (0.976-0.985)	<0.001
**CCS Category 244**	Gastric bypass and volume reduction	0.480 (0.439-0.526)	<0.001
**CCS Category 245**	Telehealth (includes telephone calls, online communication, remote monitoring and surveillance)	1.036 (1.032-1.040)	<0.001

Zero-Inflation Component

**Variables**	**Descriptions**	**Odds Ratios* (95%CIs)**	**p-values**
**Age**	<45	(Reference)	<0.001
	45-54	0.982 (0.959-1.005)	
	55-64	0.970 (0.951-0.990)	
	65-74	0.938 (0.920-0.956)	
	75-84	0.926 (0.906-0.945)	
	>=85	0.876 (0.855-0.898)	
**Admission to ICU**	ICU	0.626 (0.619-0.634)	<0.001
**Admission to Surgical Specialties**	SURGERY	0.940 (0.930-0.951)	<0.001
**Month of Admission**	January	(Reference)	<0.001

© 2025 Goto M et al. JAMA Network Open.

	February	0.995 (0.976-1.016)	
	March	0.999 (0.980-1.019)	
	April	1.017 (0.997-1.037)	
	May	0.975 (0.957-0.995)	
	June	0.947 (0.928-0.966)	
	July	0.933 (0.915-0.952)	
	August	0.929 (0.911-0.948)	
	September	0.929 (0.911-0.948)	
	October	0.912 (0.894-0.930)	
	November	0.915 (0.897-0.934)	
	December	0.942 (0.923-0.960)	
**HCC Category 1**	HIV/AIDS	0.696 (0.672-0.722)	<0.001
**HCC Category 2**	Septicemia, Sepsis, Systemic Inflammatory Response Syndrome/Shock	0.327 (0.323-0.331)	<0.001
**HCC Category 6**	Opportunistic Infections	0.812 (0.786-0.839)	<0.001
**HCC Category 8**	Metastatic Cancer and Acute Leukemia	1.082 (1.065-1.099)	<0.001
**HCC Category 23**	Other Significant Endocrine and Metabolic Disorders	1.127 (1.111-1.142)	<0.001
**HCC Category 27**	End-Stage Liver Disease	0.789 (0.772-0.807)	<0.001
**HCC Category 34**	Chronic Pancreatitis	1.287 (1.251-1.324)	<0.001
**HCC Category 35**	Inflammatory Bowel Disease	0.887 (0.864-0.910)	<0.001
**HCC Category 39**	Bone/Joint/Muscle Infections/Necrosis	0.523 (0.514-0.532)	<0.001
**HCC Category 47**	Disorders of Immunity	0.801 (0.787-0.815)	<0.001
**HCC Category 51**	Dementia With Complications	1.250 (1.200-1.303)	<0.001
**HCC Category 52**	Dementia Without Complication	1.150 (1.135-1.166)	<0.001
**HCC Category 54**	Drug/Alcohol Psychosis	1.404 (1.364-1.444)	<0.001
**HCC Category 55**	Drug/Alcohol Dependence	1.144 (1.130-1.157)	<0.001
**HCC Category 56**	Major Depressive, Bipolar, and Paranoid Disorders	1.039 (1.020-1.058)	<0.001
**HCC Category 57**	Schizophrenia	1.108 (1.084-1.133)	<0.001
**HCC Category 58**	Reactive and Unspecified Psychosis	1.123 (1.087-1.161)	<0.001
**HCC Category 59**	Personality Disorders	1.075 (1.065-1.086)	<0.001
**HCC Category 60**	Eating Disorders	1.153 (1.124-1.183)	<0.001
**HCC Category 71**	Paraplegia	0.825 (0.794-0.857)	<0.001
**HCC Category 73**	Amyotrophic Lateral Sclerosis and Other Motor Neuron Disease	1.560 (1.443-1.685)	<0.001
**HCC Category 75**	Myasthenia Gravis/Myoneural Disorders, Inflammatory and Toxic Neuropathy	1.151 (1.118-1.185)	<0.001
**HCC Category 79**	Seizure Disorders and Convulsions	1.137 (1.117-1.156)	<0.001

© 2025 Goto M et al. JAMA Network Open.

**HCC Category 80**	Coma, Brain Compression/Anoxic Damage	1.193 (1.135-1.254)	<0.001
**HCC Category 82**	Respirator Dependence/Tracheostomy Status	1.243 (1.203-1.284)	<0.001
**HCC Category 84**	Cardio-Respiratory Failure and Shock	0.820 (0.811-0.829)	<0.001
**HCC Category 85**	Congestive Heart Failure	1.196 (1.184-1.208)	<0.001
**HCC Category 86**	Acute Myocardial Infarction	1.117 (1.101-1.133)	<0.001
**HCC Category 87**	Unstable Angina and Other Acute Ischemic Heart Disease	1.274 (1.251-1.298)	<0.001
**HCC Category 88**	Angina Pectoris	1.209 (1.191-1.227)	<0.001
**HCC Category 96**	Specified Heart Arrhythmias	1.099 (1.088-1.109)	<0.001
**HCC Category 100**	Ischemic or Unspecified Stroke	1.165 (1.148-1.181)	<0.001
**HCC Category 106**	Atherosclerosis of the Extremities with Ulceration or Gangrene	0.880 (0.859-0.902)	<0.001
**HCC Category 107**	Vascular Disease with Complications	1.151 (1.135-1.168)	<0.001
**HCC Category 111**	Chronic Obstructive Pulmonary Disease	0.833 (0.825-0.841)	<0.001
**HCC Category 112**	Fibrosis of Lung and Other Chronic Lung Disorders	0.883 (0.867-0.899)	<0.001
**HCC Category 114**	Aspiration and Specified Bacterial Pneumonias	0.574 (0.564-0.584)	<0.001
**HCC Category 115**	Pneumococcal Pneumonia, Empyema, Lung Abscess	0.560 (0.547-0.573)	<0.001
**HCC Category 136**	Chronic Kidney Disease, Stage 5	1.364 (1.335-1.393)	<0.001
**HCC Category 138**	Chronic Kidney Disease, Moderate (Stage 3)	1.106 (1.094-1.117)	<0.001
**HCC Category 161**	Chronic Ulcer of Skin, Except Pressure	0.727 (0.715-0.739)	<0.001
**HCC Category 169**	Vertebral Fractures without Spinal Cord Injury	1.165 (1.132-1.198)	<0.001
**HCC Category 170**	Hip Fracture/Dislocation	1.402 (1.354-1.452)	<0.001
**HCC Category 176**	Complications of Specified Implanted Device or Graft	0.643 (0.633-0.653)	<0.001
**HCC Category 186**	Major Organ Transplant or Replacement Status	0.622 (0.597-0.649)	<0.001
**CCS Category 1**	Incision and excision of CNS	0.445 (0.395-0.500)	<0.001
**CCS Category 3**	Laminectomy, excision intervertebral disc	0.385 (0.365-0.406)	<0.001
**CCS Category 4**	Diagnostic spinal tap	0.669 (0.625-0.717)	<0.001
**CCS Category 15**	Lens and cataract procedures	3.087 (2.551-3.734)	<0.001
**CCS Category 26**	Other therapeutic ear procedures	0.643 (0.583-0.709)	<0.001
**CCS Category 28**	Plastic procedures on nose	0.342 (0.293-0.398)	<0.001
**CCS Category 29**	Oral and Dental Services	0.188 (0.164-0.217)	<0.001
**CCS Category 31**	Diagnostic procedures on nose, mouth and pharynx	0.660 (0.649-0.671)	<0.001
**CCS Category 33**	Other OR therapeutic procedures on nose, mouth and pharynx	0.416 (0.384-0.450)	<0.001
**CCS Category 37**	Diagnostic bronchoscopy and biopsy of bronchus	0.812 (0.775-0.850)	<0.001
**CCS Category 39**	Incision of pleura, thoracentesis, chest drainage	1.186 (1.143-1.231)	<0.001
**CCS Category 41**	Other non-OR therapeutic procedures on respiratory system	0.719 (0.708-0.730)	<0.001

© 2025 Goto M et al. JAMA Network Open.

**CCS Category 43**	Heart valve procedures	0.572 (0.533-0.614)	<0.001
**CCS Category 44**	Coronary artery bypass graft (CABG)	0.271 (0.253-0.289)	<0.001
**CCS Category 45**	Percutaneous transluminal coronary angioplasty (PTCA)	1.630 (1.530-1.735)	<0.001
**CCS Category 47**	Diagnostic cardiac catheterization, coronary arteriography	2.295 (2.221-2.372)	<0.001
**CCS Category 48**	Insertion, revision, replacement, removal of cardiac pacemaker or cardioverter/defibrillator	0.173 (0.166-0.180)	<0.001
**CCS Category 54**	Other vascular catheterization, not heart	0.854 (0.844-0.865)	<0.001
**CCS Category 57**	Creation, revision and removal of arteriovenous fistula or vessel-to-vessel cannula for dialysis	2.449 (2.266-2.646)	<0.001
**CCS Category 62**	Other diagnostic cardiovascular procedures	0.791 (0.770-0.813)	<0.001
**CCS Category 65**	Bone marrow biopsy	1.416 (1.298-1.545)	<0.001
**CCS Category 67**	Other therapeutic procedures, hemic and lymphatic system	1.204 (1.151-1.259)	<0.001
**CCS Category 68**	Injection or ligation of esophageal varices	0.133 (0.113-0.157)	<0.001
**CCS Category 70**	Upper gastrointestinal endoscopy, biopsy	1.422 (1.387-1.459)	<0.001
**CCS Category 71**	Gastrostomy, temporary and permanent	1.387 (1.307-1.473)	<0.001
**CCS Category 76**	Colonoscopy and biopsy	1.644 (1.585-1.705)	<0.001
**CCS Category 80**	Appendectomy	0.197 (0.183-0.213)	<0.001
**CCS Category 82**	Endoscopic retrograde cannulation of pancreas (ERCP)	0.516 (0.479-0.555)	<0.001
**CCS Category 84**	Cholecystectomy and common duct exploration	0.357 (0.340-0.375)	<0.001
**CCS Category 85**	Inguinal and femoral hernia repair	2.626 (2.372-2.908)	<0.001
**CCS Category 86**	Other hernia repair	1.791 (1.678-1.912)	<0.001
**CCS Category 88**	Abdominal paracentesis	0.597 (0.572-0.623)	<0.001
**CCS Category 91**	Peritoneal dialysis	1.046 (1.011-1.081)	0.010
**CCS Category 96**	Other OR lower GI therapeutic procedures	0.689 (0.643-0.739)	<0.001
**CCS Category 97**	Other gastrointestinal diagnostic procedures	0.887 (0.842-0.935)	<0.001
**CCS Category 98**	Other non-OR gastrointestinal therapeutic procedures	0.581 (0.557-0.606)	<0.001
**CCS Category 99**	Other OR gastrointestinal therapeutic procedures	0.833 (0.786-0.882)	<0.001
**CCS Category 100**	Endoscopy and endoscopic biopsy of the urinary tract	0.661 (0.616-0.710)	<0.001
**CCS Category 101**	Transurethral excision, drainage, or removal urinary obstruction	0.684 (0.644-0.726)	<0.001
**CCS Category 102**	Ureteral catheterization	0.628 (0.597-0.661)	<0.001
**CCS Category 103**	Nephrotomy and nephrostomy	0.672 (0.624-0.723)	<0.001
**CCS Category 104**	Nephrectomy, partial or complete	0.539 (0.503-0.579)	<0.001
**CCS Category 107**	Extracorporeal lithotripsy, urinary	0.539 (0.498-0.584)	<0.001
**CCS Category 108**	Indwelling catheter	0.680 (0.656-0.704)	<0.001
**CCS Category 109**	Procedures on the urethra	0.361 (0.318-0.410)	<0.001

© 2025 Goto M et al. JAMA Network Open.

**CCS Category 110**	Other diagnostic procedures of urinary tract	1.837 (1.654-2.041)	<0.001
**CCS Category 111**	Other non-OR therapeutic procedures of urinary tract	0.696 (0.657-0.738)	<0.001
**CCS Category 112**	Other OR therapeutic procedures of urinary tract	0.589 (0.550-0.632)	<0.001
**CCS Category 114**	Open prostatectomy	0.308 (0.288-0.329)	<0.001
**CCS Category 116**	Diagnostic procedures, male genital	0.381 (0.320-0.453)	<0.001
**CCS Category 118**	Other OR therapeutic procedures, male genital	0.156 (0.145-0.168)	<0.001
**CCS Category 124**	Hysterectomy, abdominal and vaginal	2.451 (2.078-2.891)	<0.001
**CCS Category 142**	Partial excision bone	0.577 (0.520-0.641)	<0.001
**CCS Category 146**	Treatment, fracture or dislocation of hip and femur	0.246 (0.227-0.266)	<0.001
**CCS Category 147**	Treatment, fracture or dislocation of lower extremity (other than hip or femur)	0.284 (0.254-0.318)	<0.001
**CCS Category 152**	Arthroplasty knee	0.029 (0.027-0.031)	<0.001
**CCS Category 153**	Hip replacement, total and partial	0.043 (0.040-0.047)	<0.001
**CCS Category 154**	Arthroplasty other than hip or knee	0.069 (0.062-0.077)	<0.001
**CCS Category 157**	Amputation of lower extremity	0.404 (0.385-0.424)	<0.001
**CCS Category 158**	Spinal fusion	0.449 (0.415-0.487)	<0.001
**CCS Category 159**	Other diagnostic procedures on musculoskeletal system	0.725 (0.682-0.770)	<0.001
**CCS Category 160**	Other therapeutic procedures on muscles and tendons	0.472 (0.436-0.510)	<0.001
**CCS Category 161**	Other OR therapeutic procedures on bone	0.812 (0.751-0.878)	<0.001
**CCS Category 162**	Other OR therapeutic procedures on joints	0.291 (0.260-0.325)	<0.001
**CCS Category 163**	Other non-OR therapeutic procedures on musculoskeletal system	0.449 (0.398-0.507)	<0.001
**CCS Category 164**	Other OR therapeutic procedures on musculoskeletal system	0.273 (0.225-0.330)	<0.001
**CCS Category 168**	Incision and drainage, skin and subcutaneous tissue	0.123 (0.115-0.132)	<0.001
**CCS Category 169**	Debridement of wound, infection or burn	0.389 (0.370-0.409)	<0.001
**CCS Category 170**	Excision of skin lesion	0.787 (0.743-0.833)	<0.001
**CCS Category 172**	Skin graft	0.590 (0.546-0.637)	<0.001
**CCS Category 173**	Other diagnostic procedures on skin and subcutaneous tissue	0.777 (0.732-0.824)	<0.001
**CCS Category 175**	Other OR therapeutic procedures on skin and breast	0.322 (0.296-0.350)	<0.001
**CCS Category 177**	Computerized axial tomography (CT) scan head	1.270 (1.254-1.287)	<0.001
**CCS Category 178**	CT scan chest	0.787 (0.778-0.797)	<0.001
**CCS Category 179**	CT scan abdomen	0.607 (0.600-0.613)	<0.001
**CCS Category 180**	Other CT scan	0.884 (0.869-0.899)	<0.001
**CCS Category 183**	Routine chest X-ray	0.797 (0.789-0.805)	<0.001
**CCS Category 191**	Arterio- or venogram (not heart and head)	1.533 (1.493-1.574)	<0.001
**CCS Category 192**	Diagnostic ultrasound of head and neck	1.453 (1.409-1.499)	<0.001

© 2025 Goto M et al. JAMA Network Open.

**CCS Category 193**	Diagnostic ultrasound of heart (echocardiogram)	1.051 (1.039-1.064)	<0.001
**CCS Category 195**	Diagnostic ultrasound of urinary tract	0.818 (0.781-0.856)	<0.001
**CCS Category 196**	Diagnostic ultrasound of abdomen or retroperitoneum	0.780 (0.768-0.791)	<0.001
**CCS Category 197**	Other diagnostic ultrasound	0.715 (0.706-0.724)	<0.001
**CCS Category 198**	Magnetic resonance imaging	1.093 (1.075-1.111)	<0.001
**CCS Category 200**	Nonoperative urinary system measurements	0.900 (0.888-0.912)	<0.001
**CCS Category 201**	Cardiac stress tests	2.098 (2.025-2.174)	<0.001
**CCS Category 202**	Electrocardiogram	1.198 (1.187-1.210)	<0.001
**CCS Category 203**	Electrographic cardiac monitoring	1.217 (1.193-1.242)	<0.001
**CCS Category 204**	Swan-Ganz catheterization for monitoring	0.749 (0.701-0.801)	<0.001
**CCS Category 205**	Arterial blood gases	0.803 (0.792-0.814)	<0.001
**CCS Category 206**	Microscopic examination (bacterial smear, culture, toxicology)	0.524 (0.519-0.529)	<0.001
**CCS Category 211**	Therapeutic radiology	1.497 (1.405-1.595)	<0.001
**CCS Category 212**	Diagnostic physical, occupational, and speech therapy	0.861 (0.852-0.869)	<0.001
**CCS Category 214**	Traction, splints, and other wound care	0.681 (0.662-0.700)	<0.001
**CCS Category 215**	Other physical, occupational, and speech therapy and rehabilitation	1.127 (1.108-1.146)	<0.001
**CCS Category 216**	Respiratory intubation and mechanical ventilation	0.906 (0.891-0.921)	<0.001
**CCS Category 217**	Other respiratory therapy	0.622 (0.610-0.634)	<0.001
**CCS Category 218**	Psychological and psychiatric evaluation and therapy	1.136 (1.123-1.149)	<0.001
**CCS Category 219**	Alcohol and drug management, treatment, and rehabilitation	1.677 (1.638-1.718)	<0.001
**CCS Category 220**	Ophthalmologic and otologic diagnosis and treatment	1.148 (1.113-1.184)	<0.001
**CCS Category 225**	Conversion of cardiac rhythm	2.114 (2.011-2.222)	<0.001
**CCS Category 226**	Other diagnostic radiology and related techniques	0.786 (0.778-0.794)	<0.001
**CCS Category 227**	Consultation, evaluation, and preventative care	0.879 (0.863-0.896)	<0.001
**CCS Category 228**	Prophylactic vaccinations and inoculations	1.071 (1.053-1.090)	<0.001
**CCS Category 233**	Laboratory - Chemistry and Hematology	1.395 (1.379-1.412)	<0.001
**CCS Category 236**	Nonhospital-based care (e.g., home health care, hospice)	0.946 (0.937-0.955)	<0.001
**CCS Category 237**	Ancillary Services	0.827 (0.819-0.835)	<0.001
**CCS Category 244**	Gastric bypass and volume reduction	2.851 (2.447-3.322)	<0.001
**CCS Category 245**	Telehealth (includes telephone calls, online communication, remote monitoring and surveillance)	0.805 (0.797-0.812)	<0.001
**Log(Days Present)**		0.622 (0.618-0.626)	<0.001

*Odds ratio for having no antibiotic usage

© 2025 Goto M et al. JAMA Network Open.

Abbreviations: AIDS: Acquired Immunodeficiency Syndrome; CCS: Clinical Classification Software; CT: Computed Tomography; DME: Durable Medical Equipment; GI: Gastrointestinal; HCC: Hierarchical Condition Categories; HIV: Human Immunodeficiency Virus; ICU: Intensive Care Unit; OR: Operating Room; 95%CI: 95% confidence interval

© 2025 Goto M et al. JAMA Network Open.

Supplementary Figure 1. Distribution of Hospital-Specific Random Intercepts with Adjustments

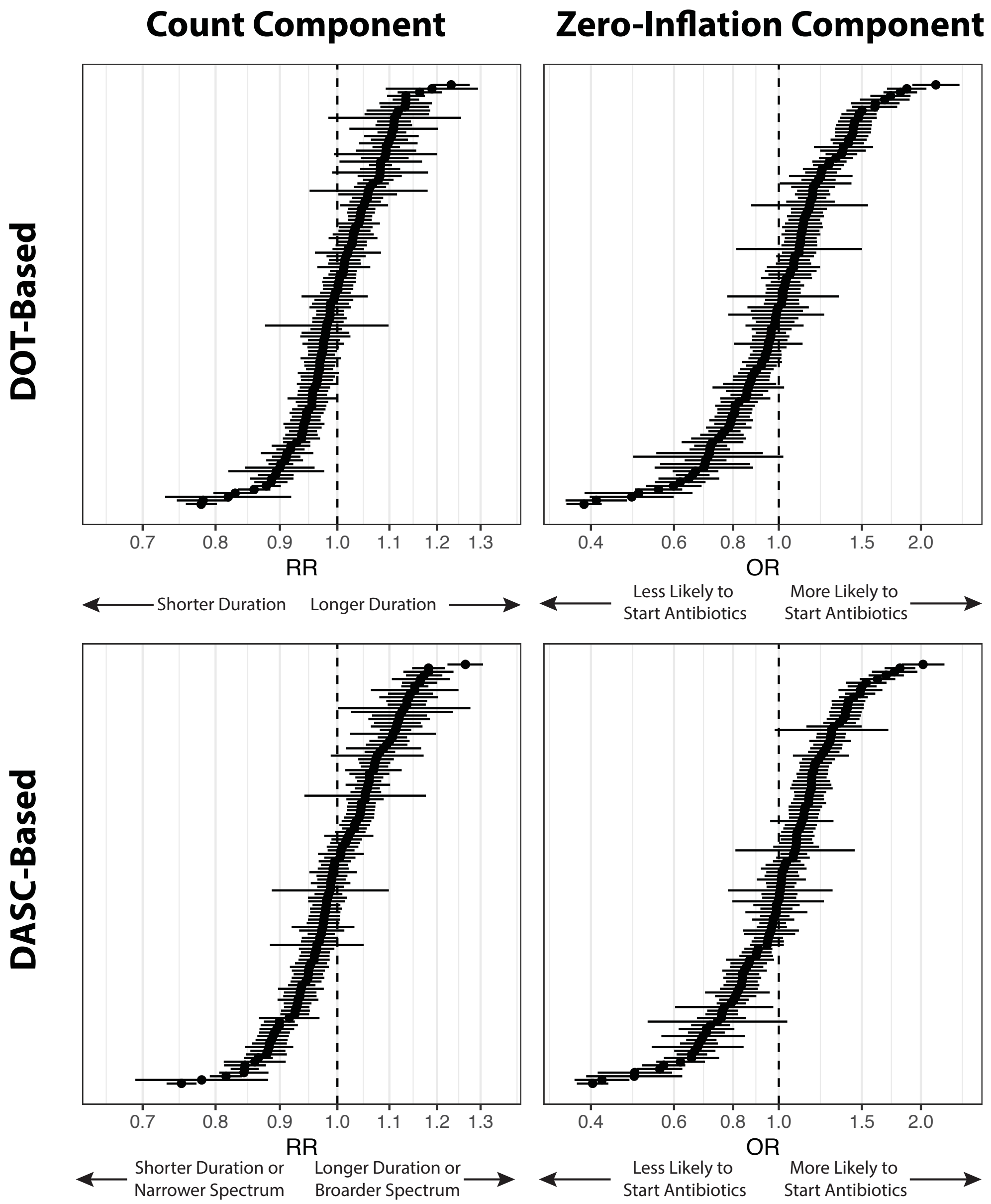

Abbreviations: DASC: Days of Antimicrobial Spectrum Coverage; DOT: Days of Therapy; OR: Odds Ratio; RR: Rate Ratio

© 2025 Goto M et al. JAMA Network Open.

<u>Supplementary Figure 2</u>. Distribution of Hospital-Specific Random Intercepts before and after Adjustments for DOT-based Method

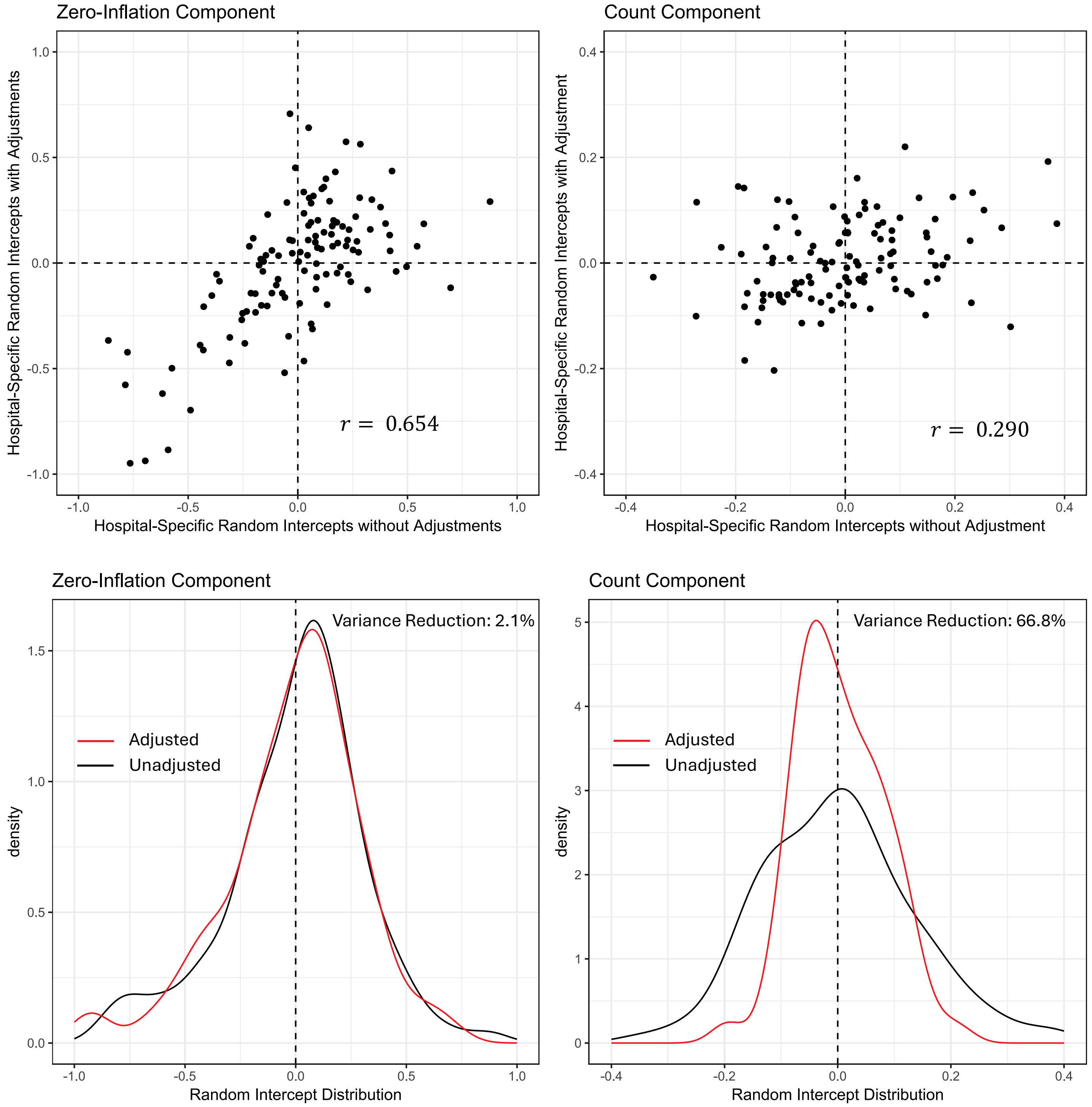

© 2025 Goto M et al. JAMA Network Open.

<u>Supplementary Figure 3</u>. Distribution of Hospital-Specific Random Intercepts before and after Adjustments for DASC-based Method

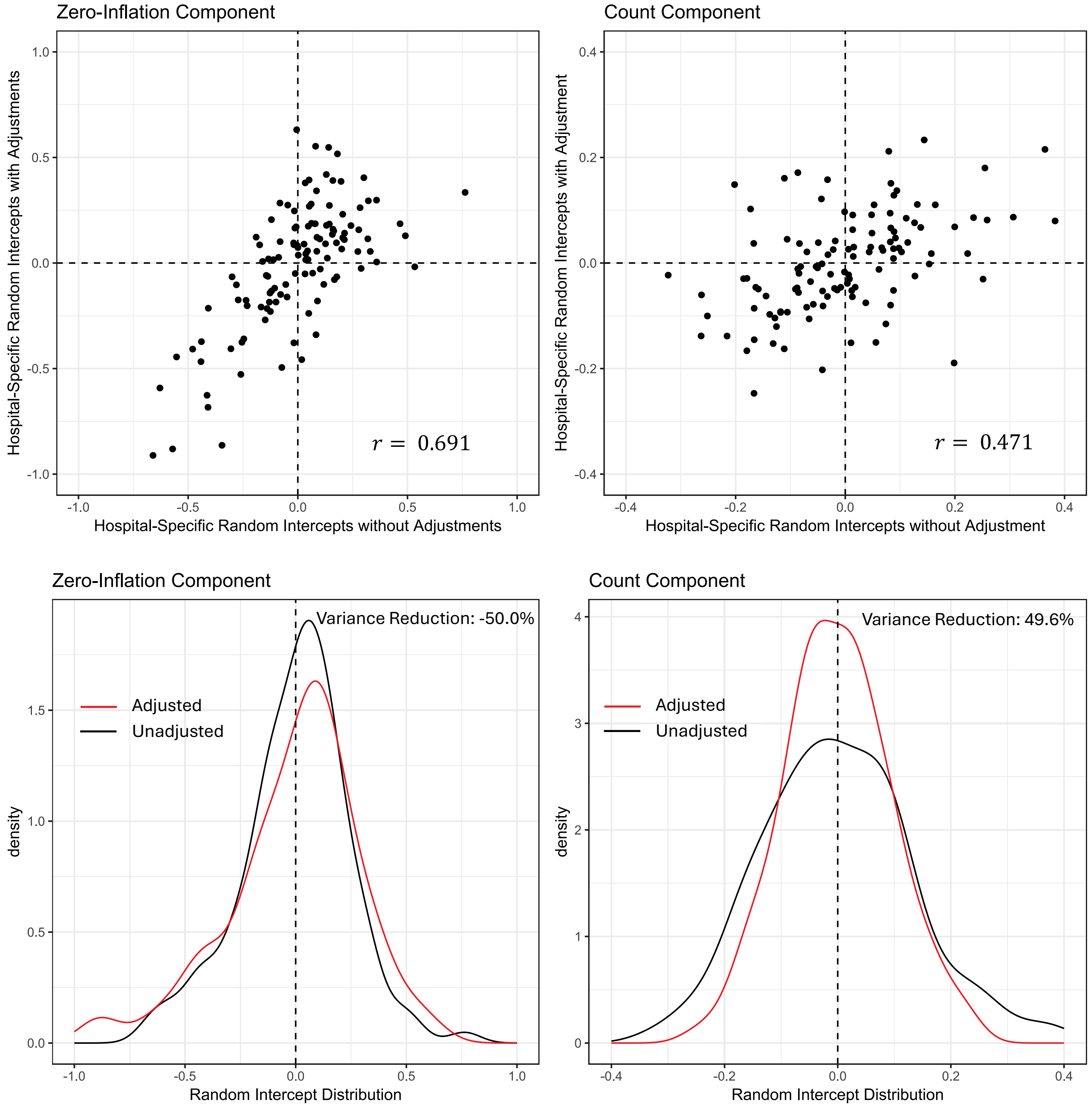

© 2025 Goto M et al. JAMA Network Open.

Technical Supplement

Impacts of Metrics Selection and Risk-Adjustment Methods on Benchmarking Inpatient Antibiotics Usage

JAMA Network Open 2025

Michihiko Goto MD MSCI, Hyunkeun Cho PhD, James A Merchant BA, Daniel J Livorsi MD MS

April 4, 2025

Contents

1 List of Abbreviations: **1**

2 Description of Dataset **2**

2.1 Dataset Aggregated at Inpatient Locations . . . 2

2.2 Dataset at Individual Patient-Level . . . 3

3 Unadjusted Benchmarking **4**

4 Risk-Adjutment by Facility- and Unit-Level Factors (Method 1) **5**

5 Risk-Adjustment by Facility-/Unit-/Patient-Level Factors (Method 2) **6**

5.1 Dataset Preparation and Screening of Predictors . . . 6

5.2 Variable Selection Process Overview . . . 8

5.3 Selecting Appropriate Lambda Values for Count and Zero-Inflation Components . . . 9

5.4 Selection of Patient-Level Predictors by LASSO . . . 10

5.5 Selection of Hospital- and Unit-Level Predictors by Backward Elimination . . . 12

5.6 Model Fit Using 2021-2022 Data . . . 16

5.7 Applying Model to 2023 Data and Calculation of P/E Ratio for Each Hospital . . . 18

1 List of Abbreviations:

- AIC: Akaike's Information Criterion
- AUR: Antimicrobial Use and Resistance
- CCS: Clinical Classification Software
- CPT: Current Procedural Terminology
- DASC: Days of Antimicrobial Spectrum Coverage
- DOT: Days of Therapy
- HCC: Hierarchical Conditions Classifications
- ICD: International Classification of Diseases
- ICU: Intensive Care Unit
- LASSO: Least Absolute Shrinkage and Selection Operator
- NHSN: National Healthcare Safety Network
- VA: US Department of Veterans Affairs

© 2025 Goto M et al. JAMA Network Open.

2 Description of Dataset

2.1 Dataset Aggregated at Inpatient Locations

This dataset contains data aggregated at inpatient locations (units) within each hospitals. Dataset structures are shown in Table 1.

Variable	Level	Data Type	Description
HospitalID	Facility	factor	Hospital Identifier
WardID	Unit	factor	Unit Identifier
Month	N/A	factor	Month of inpaient stay
ICU	Unit	logical	1 = Intensitve Care Unit (ICU); 0 = Non-ICU
SURGERY	Unit	logical	1: Surgical Units; 0: Non-Surgical Units
DaysPresent	Unit	integer	Days Present calculated per NHSN AUR protocol
DASC	Unit	integer	Days of Antimicrobial Spectrum Coverage
NHSN_ALL_ATB	Unit	integer	Days of Therapy for all antibacterials per NHSN AUR protocol
FY23ComplexityLevel	Facility	factor	VA Facility Complexity Level based on fiscal years 2021-2022 data; 5 levels (1a, 1b, 1c, 2, 3) with 1a being most complex and 3 being least complex
ICULevelScore	Facility	factor	VA-designated ICU complexity level; 5 levels (1-5) with 5 being most complex and 1 being least complex
SurgicalComplexityScore	Facility	factor	VA-designated surgical complexity level; 6 levels (0, 0.5, 1, 2, 3, 4) with 4 being most complex and 0.5 being least complex. 0 refers to the absence of the surgical service.
ResidentSlotsQuintile	Facility	factor	Quintile of educational program size (measured by the number of trainees rotating for each year); 1 being smallest and 5 being largest programs
OperatingBedQuintile	Facility	factor	Quintile of number of operating acute care beds; 1 being smallest and 5 being largest hospitals
LOSQuintile	Facility	factor	Quintile of average length of stay for each calendar year; 1 being shortest and 5 being longest groups

Table 1: Dataset Structure for the Analysis with Aggregated Data at Inpatient Locations

Because "FY23ComplexityLevel", "ICULevelScore", "SurgicalComplexityScore", and "ResidentSlotsQuintile" were based on VHA facility complexity survey for 2021-2022, they remained the same for all years. "OperatingBedQuintile" and "LOSQuintile" were assessed for each calendar year. "ICU" and "SURGERY" variables were assessed per NHSN AUR protocol.

© 2025 Goto M et al. JAMA Network Open.

2.2 Dataset at Individual Patient-Level

This dataset contains data aggregated at inpatient locations (units) within each hospitals. Dataset structures are shown in Table 1.

Variable	Level	Data Type	Description
HospitalID	Facility	factor	Hospital Identifier
WardID	Unit	factor	Unit Identifier
PatientID	Patient	factor	Patient Identifier
Month	N/A	factor	Month of inpaient stay
DaysPresent	Patient	integer	Days Present calculated per NHSN AUR protocol
DASC	Patient	integer	Days of Antimicrobial Spectrum Coverage
NHSN_ALL_ATB	Patient	integer	Days of Therapy for all antibacterials per NHSN AUR protocol
FY23ComplexityLevel	Facility	factor	Refer to Table 1
ICULevelScore	Facility	factor	Refer to Table 1
SurgicalComplexityScore	Facility	factor	Refer to Table 1
ResidentSlotsQuintile	Facility	factor	Refer to Table 1
OperatingBedQuintile	Facility	factor	Refer to Table 1
LOSQuintile	Facility	factor	Refer to Table 1
AGE_CAT	Patient	factor	Categorized age at the time of admission
Gender	Patient	logical	M: male gender; F: female gender
ICU	Patient	logical	Admission to Intensive Care Unit (ICU) service; 1 = ICU; 0 = non-ICU
SURGERY	Patient	logical	Admission to surgical services; 1: surgical service; 0: non-surgical service
HCC1-86	Patient	logical	A series of patient-level comorbidities classified by Hierarchical Conditions Classifications (HCC), based on ICD-10 diagnostic codes
CCS1-246	Patient	logical	A series of patient-level procedures performed during the inpatient stay classified by Clinical Classification Software (CCS), based on ICD-10 procedure codes and CPT codes

Table 2: Dataset Structure for the Analysis with Patient-Level Data

© 2025 Goto M et al. JAMA Network Open.

3 Unadjusted Benchmarking

For benchmarking hospitals by datasets aggregated at inpatient location-level, we used the dataset from 2021-2022 (`FacilityUnit_2021_2022`) to build a model, and applied to dataset from 2023 (`FacilityUnit_2023`). Unadjusted comprisons were performed only by `FacilityUnit_2023` that does not involve statistical models.

For unadjusted crude comparisons of hospitals in 2023 based on DOT and DASC, data was aggregated at hospital-level and DOT was normalized by the denominator (days present). To visualize the effect of using different metric (with consideration of spectrum in addition to the total consumption), we used hospital rankings based on DOT in the x-axis of the output plot (and all subsequent plots).

R code is shown below.

```
# Loading librries
library(dplyr)
library(ggplot2)

# Create a dataframe aggregated at facility level
FacilityDOT_DASC_2023 <- FacilityUnit_2023 %>%
  select(HospitalID, DaysPresent, NHSN_ALL_ATB, DASC) %>%
  group_by(HospitalID) %>%
  summarise(DaysPresent = sum(DaysPresent),
            NHSN_ALL_ATB = sum(NHSN_ALL_ATB),
            DASC = sum(DASC)) %>%
  ungroup() %>%
  mutate(NHSN_ALL_ATBperDP = (NHSN_ALL_ATB/DaysPresent)*1000, # DOT per 1,000 DPs
         DASCperDP = (DASC/DaysPresent)*1000) %>% # DASC per 1,000 DPs
  mutate(CrudeDOTRank = rank(NHSN_ALL_ATBperDP, ties.method = "random"),
         CrudeDASCRank = rank(DASCperDP, ties.method = "random"))

# Median value for DOTperDP
DOTperDP_median <- median(FacilityDOT_DASC_2023$NHSN_ALL_ATBperDP)

# Figure 1A
CrudeDOTPlot <- ggplot()+
  geom_point(data = FacilityDOT_DASC_2023,
             aes(x = CrudeDOTRank, y = NHSN_ALL_ATBperDP))+
  geom_hline(yintercept = DOTperDP_median, lty = 2)+
  scale_x_continuous(breaks = c(1,15,30,45,60,75,90,105),
                     name = "Hospital Ranking Based on Crude DOT per DP")+
  scale_y_continuous(name = "DOT per DP")+
  theme_bw()

# Median value for DASCperDP
DASCperDP_median <- median(FacilityDOT_DASC_2023$DASCperDP)

# Figure 1B
CrudeDASCPlot <- ggplot()+
  geom_point(data = FacilityDOT_DASC_2023,
             aes(x = CrudeDOTRank, y = DASCperDP))+
  geom_hline(yintercept = DASCperDP_median, lty = 2)+
  scale_x_continuous(breaks = c(1,15,30,45,60,75,90,105),
                     name = "Hospital Ranking Based on Crude DOT per DP")+
  scale_y_continuous(name = "DASC per DP")+
  theme_bw()
```

© 2025 Goto M et al. JAMA Network Open.

4 Risk-Adjutment by Facility- and Unit-Level Factors (Method 1)

For this model, we used the dataset aggregated at inpatient locations (`FacilityUnit_2021_2022` and `FacilityUnit_2023`; Section 1.1).

R codes for benchmarking based on DOT are shown below as an example. The model based on DASC was fitted with using similar codes, just replacing DOT as a target variable with DASC. We performed variable selection by backward elimination to minimize Akaike's information criterion (AIC), using `stepAIC` function of MASS package.

```
# Loading libraries
library(MASS)
library(car)
library(dplyr)

# Create offset variable
FacilityUnit_2021_2022$Log_DaysPresent <- log(FacilityUnit_2021_2022$DaysPresent)
FacilityUnit_2023$Log_DaysPresent <- log(FacilityUnit_2023$DaysPresent)

# Fit the full model using glm.nb function (MASS package)
# Replace "NHSN_ALL_ATB" with "DASC" to fit a model based on DASC
fullmodel_dot <- glm.nb(NHSN_ALL_ATB ~ FY23ComplexityLevel + ICULevelScore +
                            SurgicalComplexityScore + ResidentSlotsQuintile +
                            LOSQuintile + ICU + SURGERY +
                            Month + offset(Log_DaysPresent),
                        data = FacilityUnit_2021_2022)

# Perform backward elimination based on AIC using stepAIC function (MASS package)
final_model_dot_aic <- stepAIC(fullmodel_dot,
                               direction = "backward",
                               trace = TRUE)

# Display the summary of the final model (Table 2)
summary(final_model_dot_aic)
Anova(final_model_dot_aic, type = "III")

# Making Prediction for DOT
FacilityUnit_2023$NHSN_ALL_ATB_EXP <- predict(final_model_dot_aic,
                                              newdata = FacilityUnit_2023,
                                              type = "response")

# Calculation of DOT-based O/E ratio
HospitalID_DOT_OE_Summary <- FacilityUnit_2023 %>%
  group_by(HospitalID) %>%
  summarise(DaysPresent = sum(DaysPresent),
            NHSN_ALL_ATB = sum(NHSN_ALL_ATB),
            NHSN_ALL_ATB_EXP = sum(NHSN_ALL_ATB_EXP)) %>%
  ungroup() %>%
  mutate(OERatioDOT = NHSN_ALL_ATB/NHSN_ALL_ATB_EXP,
         DOTperDP = NHSN_ALL_ATB/(DaysPresent*1000)) %>%
  mutate(CrudeDOTRank = rank(DOTperDP, ties.method = "random"),
         OERankDOT = rank(OERatioDOT, ties.method = "random"))

# Create a plot displaying benchmarking result (Figure 1C for DOT; Figure 1D for DASC)
ggplot(data = HospitalID_DOT_OE_Summary)+
  geom_point(aes(x = CrudeDOTRank, y = OERatioDOT))+
  geom_hline(yintercept = 1, lty = 2)+
  scale_y_log10(limit = c(0.4, 2.4),
              name = "O/E Ratio for DOT")+
  scale_x_continuous(limits = c(1,116),
                     breaks = c(1,15,30,45,60,75,90,105),
                     name = "Hospital Ranking Based on Crude DOT per DP")+
  theme_bw()
```

5 Risk-Adjustment by Facility-/Unit-/Patient-Level Factors (Method 2)

For this model, we used the dataset at the individual patient-level (`Inpatient_2021_2022` and `Inpatient_2023`; Section 1.2). Again, R codes for benchmarking based on DOT were shown below as an example. The model based on DASC was fitted with using similar codes, just replacing DOT as a target variable with DASC.

5.1 Dataset Preparation and Screening of Predictors

First, we screened comorbidities (HCC categories) and procedures (CCS categories) by frequency, and removed ones with very infrequent presence of positive indicators (less than 0.5% of observations in 2021-2022 data).

```
# Loading librries
library(dplyr)

# Select columns that start with "HCC_" or "CCS_" and create a table with frequencies
selected_columns <- Inpatient_2021_2022 %>%
  select(starts_with("HCC_"), starts_with("CCS_"))

Variable_Count <- data.frame(
  Variable = character(),
  TrueCount = integer()
)

for (i in 1:dim(selected_columns)[2]){
  Variable = names(selected_columns)[i]
  TrueCount = sum(as.integer(selected_columns[,i]))
  loopCount <- cbind(Variable, TrueCount)
  Variable_Count <- rbind(Variable_Count, loopCount)
}
Variable_Count$TrueCount <- as.integer(Variable_Count$TrueCount)
Variable_Count$TotalCount <- nrow(selected_columns)
Variable_Count <- Variable_Count %>%
  mutate(TruePercentage = (TrueCount/TotalCount)*100)

# Identify variables with less than 0.5% prevalence
InfrequentVariable <- Variable_Count %>%
  filter(TruePercentage <0.5) %>%
  select(Variable) %>%
  pull()

# Remove infrequent variables
Inpatient_2021_2022 <- Inpatient_2021_2022 %>%
  select(-any_of(InfrequentVariable))

Inpatient_2023 <- Inpatient_2023 %>%
  select(-any_of(InfrequentVariable))
```

Next, we screen variables by univariate analysis to assess association between each candidate variable (HCC and CCS categories) and antimicrobial usage.

```
# Loading librries
library(dplyr)
library(parallel)
library(foreach)

# Select columns that start with "HCC_" or "CCS_" and create a table with frequencies
selected_columns <- Inpatient_2021_2022 %>%
  select(starts_with("HCC_"), starts_with("CCS_"))

DOTperDP <- Inpatient_2021_2022$NHSN_ALL_ATB/Inpatient_2021_2022$DaysPresent
```

© 2025 Goto M et al. JAMA Network Open.

```
# Initialize cluster for parallel processing
num_cores <- detectCores() - 2
cl <- makeCluster(num_cores)
registerDoParallel(cl)

# Perform parallel computation
DOTUnivariateResult <- foreach(i = 1:ncol(selected_columns), .combine = rbind, .packages = c('dplyr')) %
    dopar% {
  Variable <- selected_columns[, i]
  wilcox_test_result <- wilcox.test(DOTperDP ~ Variable)
  VariableName <- names(selected_columns)[i]
  p_value <- wilcox_test_result$p.value
  c(VariableName, p_value)
}

# Convert result to data frame and set column names
DOTUnivariateResult <- as.data.frame(DOTUnivariateResult, stringsAsFactors = FALSE)
colnames(DOTUnivariateResult) <- c("Variable", "p_value")

# Stop the cluster
stopCluster(cl)

DOTUnivariateResult$p_value <- as.numeric(DOTUnivariateResult$p_value)

DOTUnrelatedColumns <- DOTUnivariateResult %>%
  filter(p_value >= 0.05)
DOTUnrelatedColumns  # 15 variables were selected to be removed
DOTVariablesToBeRemoved <- DOTUnrelatedColumns$Variable

Inpatient_2021_2022 <- Inpatient_2021_2022 %>%
  select(-DOTVariablesToBeRemoved)
Inpatient_2023 <- Inpatient_2023 %>%
  select(-DOTVariablesToBeRemoved)
```

Lastly, we checked collinearities among HCC and CCS categories, and identify combinations with strong collinearities (defined by correlation coefficient >0.7).

```
# Loading libraries
library(dplyr)
library(caret)

# Check collinearity among HCC and CCS variables
selected_columns <- selected_columns %>%
  select(-any_of(InfrequentVariable))

# Check for multicollinearity and inspect r >0.7
cor_matrix <- cor(selected_columns, use = "pairwise.complete.obs")
highly_correlated <- findCorrelation(cor_matrix, cutoff = 0.7)
highly_correlated_vars <- rownames(cor_matrix)[highly_correlated]
print(highly_correlated_vars)
# Three sets of coliniarities were identified
# Set 1: "HCC_18" "HCC_19"
# Set 2: "HCC_134"   "HCC_136"   "CCS_58"
# Set 3: "CCS_15"  "CCS_241"

# Find which variables are highly correlated with HCC_18
highly_correlated_with_HCC_18 <- cor_matrix["HCC_18",] > 0.7
print(names(highly_correlated_with_HCC_18[highly_correlated_with_HCC_18]))
# "HCC_18" "HCC_19"

# Find which variables are highly correlated with HCC_134
highly_correlated_with_HCC_134 <- cor_matrix["HCC_134",] > 0.7
print(names(highly_correlated_with_HCC_134[highly_correlated_with_HCC_134]))
```

© 2025 Goto M et al. JAMA Network Open.

```
# "HCC_134"   "HCC_136"   "CCS_58"

# Find which variables are highly correlated with CCS_Cat15
highly_correlated_with_CCS_Cat15 <- cor_matrix["CCS_Cat15",] > 0.7
print(names(highly_correlated_with_CCS_15[highly_correlated_with_CCS_15]))
# "CCS_15"  "CCS_241"

# HCC_134 (dialysis status), HCC_136 (Chronic Kidney Disease, Stage 5), and CCS_58 (hemodialysis)
# are correlated as expected.
# Remove HCC_134 and CCS_58, as HCC_136 had stronger association with antimicrobial use.

# HCC_18 (diabetes with chronic complications) and HCC_19 (diabetes without complication) are
# correlated. Remove HCC_19, as HCC_18 had stronger association with antimicrobial use.

# CCS_15 (Lens and cataract procedures) and CCS_241 (Visual aids and other optical supplies) are
# correlated. Remove CCS_241, as CCS_15 had stronger association with antimicrobial use.

Inpatient_2021_2022 <- Inpatient_2021_2022 %>%
  select(-c(HCC_134, CCS_58, HCC_19, CCS_241))

Inpatient_2023 <- Inpatient_2023 %>%
  select(-c(HCC_134, CCS_58, HCC_19, CCS_241))
```

5.2 Variable Selection Process Overview

To select patient-level variables, we randomly split the dataset into ten random subsets and performed cross-validation in each subset. Our approach differs from traditional single-pass approaches. Our training dataset (data from 2021-2022) contains approximately 2 million observations and 300 patient-level candidate variables. In addition, all variables were considered for both the zero-inflation and the count components, effectively doubling the number of candidate variables. Applying LASSO with cross-validation directly to the full dataset is computationally intensive and presents practical limitations due to its very large memory space requirements (not just to us but to any researchers who may want to replicate our work). Therefore, we adapted a strategy similar in spirit to stability selection (Meinshausen N and Bühlmann P 2010, DOI: 10.1111/j.1467-9868.2010.00740.x): we performed LASSO (with internal cross-validation to determine the most appropriate lambda value) independently across 10 random subsets (each 200,000 observations), and selected only variables that were consistently chosen across all 10 subsets.

Our approach was motivated by three reasons:

1. Reducing computational burden: Each subset is small enough to allow efficient LASSO with cross-validation using standard computing infrastructure. We used a workstation equipped with 12 CPU cores and 64 GB of memory, which allowed us to run LASSO models in parallel. We attempted to perform LASSO and traditional backward elimination with the entire training dataset first but could not fit into our computational environment.

2. Improving variable stability and avoiding overfitting: Rather than relying on a single model fit, our approach emphasizes repeatedly selected variables across multiple random subsets. This guards against the inclusion of variables whose selection is driven by random noise in any one sample and mimics the benefits of stability selection. Notably, variables that appeared in fewer than all 10 subsets were often weakly predictive and exhibited low coefficients when re-evaluated on the full dataset.

3. Preserving the benefits of LASSO while enhancing generalizability: After selecting the stable set of variables (those selected in all 10 subsets), we refit a final model on the full dataset using only these variables. This refitting step allowed us to recover much of the statistical efficiency and predictive accuracy that could be lost by operating only on subsets while maintaining an interpretable and robust variable set.

We recognize that our strategy might reduce LASSO's full-model shrinkage behavior. However, because variables needed to pass a stricter inclusion threshold (selected in 10/10 subsets), this approach acts as a conservative filter, minimizing the risk of false positives in variable selection and increasing stability and applicability. We also recognize that our conservative approach could potentially exclude important variables that were not selected in every iteration. However, given each random subset has approximately 200,000 observations, we believe the likelihood was minimal.

After selecting patient-level variables with the approach above, we selected facility-level variables with a backward elimination strategy to minimize AIC while adjusting for patient-level variables. We decided to take this approach for two primary reasons:

© 2025 Goto M et al. JAMA Network Open.

1. Model complexity and parsimony: Even if a facility-level variable appeared consistently in the subset-based LASSO models, its contribution to overall model fit (once adjusted for selected patient-level variables) could still be minimal. We, therefore, used backward elimination based on AIC to ensure that all retained hospital-level variables provided meaningful improvements in model performance, thereby enhancing model interpretability and avoiding unnecessary complexity in the hierarchical structure.

2. Current lack of LASSO support for hierarchical zero-inflated negative binomial (ZINB) models in R: To our knowledge, there is currently no published package in R that performs LASSO variable selection for hierarchical ZINB models. As such, our initial LASSO-based screening was applied to non-hierarchical patient-level models. Once we moved to the full hierarchical framework, particularly in the context of hospital-level variables, we relied on model-based selection methods available for mixed-effect ZINB models. Backward AIC selection offered a practical and statistically justifiable tool for refining the hospital-level structure after patient-level predictors had been stabilized.

5.3 Selecting Appropriate Lambda Values for Count and Zero-Inflation Components

```
# Loading libraries
library(pscl)
library(mpath)
library(dplyr)
library(parallel)
library(doParallel)
library(foreach)

# Create offset variable
Inpatient_2021_2022$Log_DaysPresent <- log(Inpatient_2021_2022$DaysPresent)

# Preallocate memory for data frame and list for plot data
lambda.cv.df <- data.frame(i = integer(),
                           lambda.count.min = double(),
                           lambda.zero.min = double())

# Generate identifier for random subsets
set.seed(12345)
Inpatient_2021_2022 <- Inpatient_2021_2022 %>%
  group_by(HospitalID) %>%
  mutate(randomnumber = sample(0:9, size = n(), replace = TRUE))%>%
  ungroup()

# Create formula object
outcome_variable <- "NHSN_ALL_ATB"
pt.predictors <- Inpatient_2021_2022 %>%
  select(AGE_CAT, Gender, ICU, SURGERY, Month,
         starts_with("HCC_"), starts_with("CCS_Cat")) %>%
  colnames()
offset_variable <- "Log_DaysPresent"
fullmodel_formula <- as.formula(paste(outcome_variable,
                                      "~",
                                      paste(pt.predictors, collapse = "+"),
                                      "+ offset(", offset_variable, ")", # Log_DaysPresent as offset
                                      "| ",
                                      paste(pt.predictors, collapse = "+"),
                                      "+ ", offset_variable)) # Log_DaysPresent as a regular variable

# Fit the full model to estimate start values for LASSO (20% random subset)
set.seed(123456)
Inpatient_2021_2022_sample <- Inpatient_2021_2022 %>%
  sample_frac(0.2)
dot.fullmodel.sample <- zeroinfl(fullmodel_formula,
                        data = Inpatient_2021_2022_sample,
                        dist = "negbin",
```

© 2025 Goto M et al. JAMA Network Open.

```
                       method = "L-BFGS-B")
start_values <- coef(dot.fullmodel.sample)
rm(Inpatient_2021_2022_sample)
rm(dot.fullmodel.sample)

# Register the parallel backend
no_cores <- detectCores() - 2 # Reserving two cores for general operations
cl <- makeCluster(no_cores)
registerDoParallel(cl)

# Set the range of lambda values to be tried
lambda_values <- seq_log(1e-5, 1e-1, 5)

# Use foreach for the parallel loop
cv.results <- foreach(i = 0:9, .packages = c("mpath", "dplyr")) %dopar% {
  # Randomly select 10% of dataset grouped by HospitalID
  Inpatient_Sampled <- Inpatient_2021_2022 %>%
    group_by(HospitalID) %>%
    filter(randomnumber %% 10 == i) %>%
    ungroup() %>%
    select(-c("HospitalID", "randomnumber"))

  # 5-fold cross-validation to determine the best lambda values for each subset
  cv.model.lasso <- cv.zipath(fullmodel_formula,
                              data = Inpatient_Sampled,
                              family = "negbin",
                              nfolds = 5,
                              lambda.count = lambda_values,
                              lambda.zero = lambda_values,
                              start = start_values,
                              penalty = "enet")

  # Return the results for this iteration
  list(i, cv.model.lasso$lambda.optim$count, cv.model.lasso$lambda.optim$zero)
}

# Stop the cluster
stopCluster(cl)

# Process results
for (result in cv.results) {
  lambda.cv.df <- rbind(lambda.cv.df, data.frame(i = result[[1]], lambda.count.min = result[[2]], lambda.
    zero.min = result[[3]]))
}
```

For both DOT and DASC, the majority of subsets selected $1e^{-3}$ as the lambda value for both count and zero-inflated components. We used this lambda value for the next step.

5.4 Selection of Patient-Level Predictors by LASSO

By using lambda values determined in the previous step, we perform variable selection by LASSO for patient-level variables. To avoid overfitting and reduce computational burden, we again split the dataset to 10 random subsets and performed variable selections on each subset.

```
# Loading libraries
library(dplyr)
library(mpath)
library(parallel)
library(doParallel)
library(foreach)

# Set up a parallel backend with the number of cores
```

© 2025 Goto M et al. JAMA Network Open.

```
no_cores <- detectCores() - 2
cl <- makeCluster(no_cores)
registerDoParallel(cl)

# Load the necessary packages on each of the workers
clusterEvalQ(cl, {
  library(tidyverse)
  library(mpath)
  library(zic)
  library(pscl)
})

# Use foreach instead of the traditional for loop
results <- foreach(i = 0:9) %dopar% {

  # Randomly select 10% of dataset grouped by HospitalID
  Inpatient_Sampled <- Inpatient_2021_2022 %>%
    filter(randomnumber %% 10 == i) %>%
    ungroup() %>%
    select(-HospitalID)

  model.lasso <- zipath(fullmodel_formula, # Formula defied in the previous step
                        data = Inpatient_Sampled,
                        family = "negbin",
                        lambda.count = 1e-3,
                        lambda.zero = 1e-3,
                        start = start_values, # Start values from the previous step
                        penalty="enet")

  predictors.count <- as.data.frame(model.lasso$coefficients$count) %>%
    rownames_to_column()
  names(predictors.count) <- c("variable","coef")

  # Create a list of selected predictors for count component
  predictors.count <- predictors.count %>%
    filter(coef != 0) %>%
    mutate(variable = str_replace_all(variable, "TRUE", "")) %>%
    mutate(variable = str_replace_all(variable, "GenderM", "Gender")) %>%
    mutate(variable = ifelse(str_detect(variable, "AGE_CAT"), "AGE_CAT", variable)) %>%
    mutate(variable = ifelse(str_detect(variable, "Month"), "Month", variable)) %>%
    filter(variable != "(Intercept)") %>%
    distinct(variable) %>%
    mutate(sample = i)
  predictors.count <- predictors.count %>%
    select(sample, variable)

  predictors.zero <- as.data.frame(model.lasso$coefficients$zero) %>%
    rownames_to_column()
  names(predictors.zero) <- c("variable","coef")

  # Create a list of selected predictors for zero-inflation component
  predictors.zero <- predictors.zero %>%
    filter(coef != 0) %>%
    mutate(variable = str_replace_all(variable, "TRUE", "")) %>%
    mutate(variable = str_replace_all(variable, "GenderM", "Gender")) %>%
    mutate(variable = ifelse(str_detect(variable, "AGE_CAT"), "AGE_CAT", variable)) %>%
    mutate(variable = ifelse(str_detect(variable, "Month"), "Month", variable)) %>%
    filter(variable != "(Intercept)") %>%
    distinct(variable) %>%
    mutate(sample = i)
  predictors.zero <- predictors.zero %>%
    select(sample, variable)
```

© 2025 Goto M et al. JAMA Network Open.

```
  # seleted.variables.zero <- rbind(seleted.variables.zero, predictors.zero)

  list(predictors.count = predictors.count, predictors.zero = predictors.zero)
}

# Combine the results
seleted.variables.count <- do.call(rbind, lapply(results, `[[`, "predictors.count"))
seleted.variables.zero <- do.call(rbind, lapply(results, `[[`, "predictors.zero"))

# Stop the cluster
stopCluster(cl)

#Creating tables with variables with selection frequency
pt.predictors <- data.frame(variable = pt.predictors)

freq.variables.count <- seleted.variables.count %>%
  group_by(variable) %>%
  summarise(n = n()) %>%
  ungroup()
freq.variables.count<- pt.predictors %>%
  left_join(freq.variables.count, by = "variable") %>%
  replace_na(list(variable = 0))

predictors.zero <- rbind(pt.predictors, "Log_DaysPresent")
freq.variables.zero <- seleted.variables.zero %>%
  group_by(variable) %>%
  summarise(n = n()) %>%
  ungroup()
freq.variables.zero<- predictors.zero %>%
  left_join(freq.variables.zero, by = "variable") %>%
  replace_na(list(variable = 0))
```

5.5 Selection of Hospital- and Unit-Level Predictors by Backward Elimination

For this step, we fit a model with all facility-/unit-level predictors and selected patient-level predictors from the previous step first, then perform a backward elimination variable selection for facility-/unit-level predictors.

First, we create formula objects with all facility-/unit-level predictors and selected patient-level predictors.

```
# Loading libraries
library(dplyr)

# Patient-level predictors selected by all 10 subsets in the previous step
predictors.count <- freq.variables.count %>%
  filter(n ==10) %>%
  select(variable) %>%
  pull()
predictors.count

predictors.zero <- freq.variables.zero %>%
  filter(n ==10) %>%
  select(variable) %>%
  pull()
predictors.zero

# Define the outcome variable (Replace with DASC for DASC-based model)
outcome_variable <- "NHSN_ALL_ATB"

# Define the random intercepts for the models
random.intercepts <- "(1|HospitalID)"

# The list of facility-/unit-level predictors
```

© 2025 Goto M et al. JAMA Network Open.

```
facilityunit.predictors <- c("FY23ComplexityLevel", "ICULevelScore", "SurgicalComplexityScore",
                             "ResidentSlotsQuintile", "OperatingBedQuintile", "LOSQuintile")

offset_variable <- "Log_DaysPresent"

variables_list <- unique(c(outcome_variable, facilityunit.predictors,
                         sort(c(predictors.count, predictors.zero)), offset_variable, "HospitalID"))

Inpatient_2021_2022 <- Inpatient_2021_2022 %>%
  select(any_of(variables_list))
Inpatient_2023 <- Inpatient_2023 %>%
  select(any_of(variables_list))

# Define the formulas for the single-level and multi-level models
# to fit models using glmmTMB package
count.formula.single <- as.formula(paste(outcome_variable,
                                         "~ ",
                                         paste(facilityunit.predictors, collapse = " + "),
                                         " + ",
                                         paste(predictors.count, collapse = " + "),
                                         " + offset(Log_DaysPresent)"))

zero.formula.single <- as.formula(paste("~ ",
                                        paste(facilityunit.predictors, collapse = " + "),
                                        " + ",
                                        paste(predictors.zero, collapse = " + ")))

count.formula.mtl <- as.formula(paste(outcome_variable,
                                      "~ ",
                                      paste(facilityunit.predictors, collapse = " + "),
                                      " + ",
                                      paste(predictors.count, collapse = " + "),
                                      " + offset(Log_DaysPresent)",
                                      "+ ", random.intercepts))

zero.formula.mtl <- as.formula(paste("~ ",
                                     paste(facilityunit.predictors, collapse = " + "),
                                     " + ",
                                     paste(predictors.zero, collapse = " + "),
                                     "+ ", random.intercepts))
```

Then, we fit a model with formula objects defined in the previous step. To reduce a computational burden, we fit a single-level model without hospital-specific random intercepts first to obtain start values of fixed-effect coefficients, then fit a model with hospital-specific random intercepts.

```
# Loading libraries
library(dplyr)
library(glmmTMB)
library(parallel)

# Fit a single-level model without hospital-specific random intercept first

# Detect the number of cores in the system and reserve two for other processes
num_cores <- detectCores()-2

# Set up the control parameters for the glmmTMB optimizer (adjust as needed)
ctrl <- glmmTMBControl(
  optimizer = optim,
  optArgs = list(method = "L-BFGS-B", maxfun = 100000),
  eigval_check = FALSE,
  parallel = num_cores
)
```

```
# Fit the single-level model using the glmmTMB function
model.all.atb.single <- glmmTMB(count.formula.single,
                                ziformula = zero.formula.single,
                                family = nbinom2(link = "log"), # Negative binomial distribution
                                data = Inpatient_2021_2022, # Dataset
                                control = ctrl) # Control parameters

# Get the coefficients from the simpler model to use as starting values for the multi-level model
start_vals <- list(beta = fixef(model.all.atb.single)$cond,
                   betazi = fixef(model.all.atb.single)$zi,
                   theta = sigma(model.all.atb.single))
rm(model.all.atb.single)

# Fit a multi-level full model using the glmmTMB function
model.all.atb.mtl <- glmmTMB(count.formula.mtl,
                             ziformula = zero.formula.mtl,
                             family = nbinom2, # Negative binomial distribution
                             data = Inpatient_2021_2022, # Dataset
                             start = start_vals,
                             control = ctrl) # Control parameters
```

Using this model with all facility-/unit-level predictors and selected patient-level predictors as a baseline model, we perform backward elimination variable selections for facility-/unit-level predictors. Similarly to the selection process for patient-level predictors, we conduct this process in 10 random subsets and select predictors selected in all subsets.

```
# Loading libraries
library(dplyr)
library(glmmTMB)
library(parallel)
library(car)

# Preallocate memory for the output dataset
Backward.Summary <- data.frame(
  Sample = integer(),
  Iteration = integer(),
  Variable.Removed = character(),
  Variable.Component = character(),
  AIC = double()
)

# Fixed effect coefficients for start values
coef_start.cond <- fixef(model.all.atb.mtl)$cond
coef_start.zi <- fixef(model.all.atb.mtl)$zi

# Generate random numbers for subset selection
set.seed(1234)
Inpatient_2021_2022 <- Inpatient_2021_2022 %>%
  group_by(HospitalID) %>%
  mutate(randomnumber = sample(0:9, size = n(), replace = TRUE))%>%
  ungroup()

# Detect the number of cores in the system and reserve two for other processes
num_cores <- detectCores()-2

# Set up the control parameters for the glmmTMB optimizer (adjust as needed)
ctrl <- glmmTMBControl(
  optimizer = optim,
  optArgs = list(method = "L-BFGS-B", maxfun = 100000),
  eigval_check = FALSE,
  parallel = num_cores
)
```

© 2025 Goto M et al. JAMA Network Open.

```
# Use loop to conduct backward elimination of facility/unit level variables
for (j in 0:9) {  # 10 subsets

  start_vals <- list(beta = coef_start.cond,
                     betazi = coef_start.zi)
  Inpatient_2021_2022_Sample <- Inpatient_2021_2022 %>%
    filter(randomnumber == j)

  update.model.all.atb.mtl <- glmmTMB(count.formula.mtl,
                                      ziformula = zero.formula.mtl,
                                      family = nbinom2, # Negative binomial distribution
                                      data = Inpatient_2021_2022_Sample, # Dataset
                                      start = start_vals,
                                      control = ctrl) # Control parameters

  # There are 6 facility-/unit-level predictors each in count and zero-inflated components
  for (i in 1:12){
    # Estimate chisq values for all variables in the model
    type3_pvalues_dot.multi.cond <- Anova(update.model.all.atb.mtl, type = "III", component = "cond")
    names(type3_pvalues_dot.multi.cond) <- c("SumSq", "Df", "FValue", "p_value")
    type3_pvalues_dot.multi.zero <- Anova(update.model.all.atb.mtl, type = "III", component = "zi")
    names(type3_pvalues_dot.multi.zero) <- c("SumSq", "Df", "FValue", "p_value")

    # Identify the variable with largest p-values
    ToRemove.cond <- type3_pvalues_dot.multi.cond %>%
      mutate(Variable = row.names(.)) %>%
      filter(Variable %in% c("FY23ComplexityLevel", "ICULevelScore", "SurgicalComplexityScore",
                             "ResidentSlotsQuintile", "OperatingBedQuintile", "LOSQuintile")) %>%
      filter(p_value == max(p_value)) %>%
      select(Variable, p_value) %>%
      mutate(component = "cond")

    ToRemove.zero <- type3_pvalues_dot.multi.zero %>%
      mutate(Variable = row.names(.)) %>%
      filter(Variable %in% c("FY23ComplexityLevel", "ICULevelScore", "SurgicalComplexityScore",
                             "ResidentSlotsQuintile", "OperatingBedQuintile", "LOSQuintile")) %>%
      filter(p_value == max(p_value)) %>%
      select(Variable, p_value) %>%
      mutate(component = "zero")

    ToRemove <- rbind(ToRemove.cond, ToRemove.zero) %>%
      filter(p_value == min(p_value)) %>%
      select("Variable", "component")

    ToRemove.variable <- ToRemove %>%
      select(Variable) %>%
      pull()

    ToRemove.component <- ToRemove %>%
      select(component) %>%
      pull()

    current.parameters <- update.model.all.atb.mtl$fit$par

    # Set start values
    coef_current.cond <- fixef(update.model.all.atb.mtl)$cond
    coef_current.zi <- fixef(update.model.all.atb.mtl)$zi

    if (ToRemove.component == "cond") {
      coef_current.cond <- coef_current.cond[!grepl(ToRemove.variable, names(coef_current.cond))]
    } else {
      coef_current.zi <- coef_current.zi[!grepl(ToRemove.variable, names(coef_current.zi))]
```

```
    }

    start_vals <- list(beta = coef_current.cond,
                       betazi = coef_current.zi)

    predictor.cond <- rownames(type3_pvalues_dot.multi.cond)
    predictor.zi <- rownames(type3_pvalues_dot.multi.zero)

    predictor.cond <- predictor.cond[sapply(predictor.cond, function(x) x != "(Intercept)")]
    predictor.zi <- predictor.zi[sapply(predictor.zi, function(x) x != "(Intercept)")]

    if (ToRemove.component == "cond") {
      predictor.cond <- predictor.cond[sapply(predictor.cond, function(x) x != ToRemove.variable)]
    } else {
      predictor.zi <- predictor.zi[sapply(predictor.zi, function(x) x != ToRemove.variable)]
    }

    formula.count <- as.formula(paste("NHSN_ALL_ATB ~ ",
                                      paste(predictor.cond, collapse = " + "),
                                      " + offset(Log_DaysPresent)",
                                      " + (1 | HospitalID)"))
    formula.zi <- as.formula(paste("~ ",
                                   paste(predictor.zi, collapse = " + "),
                                   " + (1 | HospitalID)"))

    update.model.all.atb.mtl <- glmmTMB(data = Inpatient_2021_2022_Sample,
                                        formula.count,
                                        ziformula = formula.zi,
                                        start = start_vals,
                                        control = ctrl)

    Backward.Summary <- Backward.Summary %>%
      add_row(Sample = j,
              Iteration = i,
              Variable.Removed = ToRemove.variable,
              Variable.Component = ToRemove.component,
              AIC = AIC(update.model.all.atb.mtl))

  }
  print(paste("Sample #", j, " completed at ", Sys.time()))
}

MinimumAICStep <- Backward.Summary %>%
  filter(!is.na(Variable.Removed)) %>%
  group_by(Sample) %>%
  filter(AIC == min(AIC, na.rm = TRUE)) %>%
  ungroup() %>%
  select(Sample, Iteration) %>%
  rename(MinimumIteration = Iteration)

SelectionFrequency <- Backward.Summary %>%
  filter(!is.na(Variable.Removed)) %>%
  left_join(MinimumAICStep, by = "Sample") %>%
  filter(Iteration >= MinimumIteration) %>%
  select(Variable.Removed, Variable.Component) %>%
  count(Variable.Removed,Variable.Component)

# No facility-/unit-level predictors was selected in all 10 subsets
```

5.6 Model Fit Using 2021-2022 Data

© 2025 Goto M et al. JAMA Network Open.

```r
# Loading libraries
library(dplyr)
library(glmmTMB)
library(parallel)
library(car)

# Define the formulas for the single-level and multi-level models
count.formula.single <- as.formula(paste(outcome_variable,
                                         "~ ",
                                         paste(predictors.count, collapse = " + "),
                                         " + offset(Log_DaysPresent)"))

zero.formula.single <- as.formula(paste("~ ",
                                        paste(predictors.zero, collapse = " + ")))

count.formula.mtl <- as.formula(paste(outcome_variable,
                                      "~ ",
                                      paste(predictors.count, collapse = " + "),
                                      " + offset(Log_DaysPresent)",
                                      "+ ", random.intercepts))

zero.formula.mtl <- as.formula(paste("~ ",
                                     paste(predictors.zero, collapse = " + "),
                                     "+ ", random.intercepts))

# Detect the number of cores in the system and reserve two for other processes
num_cores <- detectCores()-2

# Set up the control parameters for the glmmTMB optimizer (adjust as needed)
ctrl <- glmmTMBControl(
  optimizer = optim,
  optArgs = list(method = "L-BFGS-B", maxfun = 100000),
  eigval_check = FALSE,
  parallel = num_cores
)

# Fit the single-level model using the glmmTMB function
model.all.atb.single <- glmmTMB(count.formula.single,
                                ziformula = zero.formula.single,
                                family = nbinom2(link = "log"), # Negative binomial distribution
                                data = Inpatient_2021_2022, # Dataset
                                control = ctrl) # Control parameters

# Get the coefficients from the simpler model to use as starting values for the multi-level model
current.parameters <- model.all.atb.single$fit$par

# Set start values
coef_current.cond <- fixef(model.all.atb.single)$cond
coef_current.zi <- fixef(model.all.atb.single)$zi
start_vals <- list(beta = coef_current.cond,
                   betazi = coef_current.zi)

# Fit a multi-level model using the glmmTMB function
model.all.atb.mtl.final <- glmmTMB(count.formula.mtl,
                             ziformula = zero.formula.mtl,
                             family = nbinom2, # Negative binomial distribution
                             data = Inpatient_2021_2022, # Dataset
                             start = start_vals,
                             control = ctrl) # Control parameters

# Print the summary of the model
```

© 2025 Goto M et al. JAMA Network Open.

```
summary(model.all.atb.mtl.final)
Anova(model.all.atb.mtl.final, type = "III", component = "cond")
Anova(model.all.atb.mtl.final, type = "III", component = "zi")
```

5.7 Applying Model to 2023 Data and Calculation of P/E Ratio for Each Hospital

In this step, we apply fixed effect coefficients estimated by 2021-2022 data to the dataset from 2023, then fit random-effect model to estimate only hospital-specific random intercepts. We create a covariate matrix and multiply it by vectors of fixed-effect coefficient for count and zero-inflation components separately. Output vectors will be included to the model as offset variables (i.e., fixed coefficients of 1).

```
# Loading libraries
library(dplyr)
library(glmmTMB)
library(parallel)
library(ggplot2)

# Manually code dummy variables for 2023 data
Inpatient_2023_dummy <- Inpatient_2023 %>%
  mutate(Month2 = ifelse(Month == '2',1,0)) %>%
  mutate(Month3 = ifelse(Month == '3',1,0)) %>%
  mutate(Month4 = ifelse(Month == '4',1,0)) %>%
  mutate(Month5 = ifelse(Month == '5',1,0)) %>%
  mutate(Month6 = ifelse(Month == '6',1,0)) %>%
  mutate(Month7 = ifelse(Month == '7',1,0)) %>%
  mutate(Month8 = ifelse(Month == '8',1,0)) %>%
  mutate(Month9 = ifelse(Month == '9',1,0)) %>%
  mutate(Month10 = ifelse(Month == '10',1,0)) %>%
  mutate(Month11 = ifelse(Month == '11',1,0)) %>%
  mutate(Month12 = ifelse(Month == '12',1,0)) %>%
  select(-Month) %>%
  mutate(AGE_45_54 = ifelse(AGE_CAT == 'AGE_45_54',1,0))%>%
  mutate(AGE_55_64 = ifelse(AGE_CAT == 'AGE_55_64',1,0))%>%
  mutate(AGE_65_74 = ifelse(AGE_CAT == 'AGE_65_74',1,0))%>%
  mutate(AGE_75_84 = ifelse(AGE_CAT == 'AGE_75_84',1,0))%>%
  mutate(AGE_GE85 = ifelse(AGE_CAT == 'AGE_GE85',1,0))%>%
  select(-AGE_CAT) %>%
  mutate(GenderM = ifelse(Gender == 'M',1,0))%>%
  select(-Gender) %>%
  rename_with(~ paste0(., "TRUE"), starts_with("CCS")) %>%
  rename_with(~ paste0(., "TRUE"), starts_with("HCC")) %>%
  rename_with(~ paste0("AGE_CAT", .), starts_with("AGE"))

# Convert all logical variables to integer
Inpatient_2023_dummy <- Inpatient_2023_dummy %>%
  mutate_if(is.logical, as.integer)

fixed_effects_count <- fixef(model.all.atb.mtl.final)$cond
varnames_count <- names(fixed_effects_count)[2:length(fixed_effects_count)]
fixed_effects_zero <- fixef(model.all.atb.mtl.final)$zi
varnames_zero <- names(fixed_effects_zero)[2:length(fixed_effects_zero)]

# Create matrices for count and zi components
Intercept <- rep(1, nrow(Inpatient_2023_dummy))
Log_DaysPresent <- Inpatient_2023_dummy$Log_DaysPresent

count_matrix <- Inpatient_2023_dummy %>%
  select(all_of(varnames_count))
count_matrix <- cbind(Intercept, count_matrix)
count_matrix <- as.matrix(count_matrix)
offset_count <- count_matrix %*% fixed_effects_count
```

© 2025 Goto M et al. JAMA Network Open.

```
offset_count <- offset_count + Log_DaysPresent

zero_matrix <- Inpatient_2023_dummy %>%
  select(all_of(varnames_zero))
zero_matrix <- cbind(Intercept, zero_matrix)
zero_matrix <- as.matrix(zero_matrix)
offset_zero <- zero_matrix %*% fixed_effects_zero

# Create a dataset with hospital ID, target variable, and offsets
Inpatient_2023_offset <- Inpatient_2023 %>%
  cbind(offset_count) %>%
  cbind(offset_zero) %>%
  select(c(HospitalID, NHSN_ALL_ATB, offset_count, offset_zero))

# Prediction with fixed effect estimates
mu <- exp(offset_count)
pi <- plogis(offset_zero)
NHSN_ALL_ATB_EXP <- (1 - pi) * mu
# This can be interpreted as "the amount of antibiotics a hypothetical hospital in 2021-2022
# would have used if it saw same patient in 2023."

# Fit random-effect model
# Control parameters for glmmTMB function (adjust as needed)
num_cores <- detectCores()-2
ctrl <- glmmTMBControl(
  optimizer = optim,
  optArgs = list(method = "L-BFGS-B", maxfun = 100000),
  eigval_check = FALSE,
  parallel = num_cores
)

# Fit a model to estimate hospital-specific random intercepts for 2023 data
model.all.atb.pred.2023 <- glmmTMB(NHSN_ALL_ATB ~ offset(offset_count) + (1|HospitalID),
                                   data = Inpatient_2023_offset,
                                   ziformula = ~ offset(offset_zero) + (1|HospitalID),
                                   family = nbinom2,
                                   control = ctrl) # Control parameters

# Prediction from hierarchical model
# Prediction with random intercept
NHSN_ALL_ATB_PRED <- predict(model.all.atb.pred.2023, type = "response")
# This can be interpreted as "assuming included factors have the same impacts, the amount of
# antibiotics each hospital in 2023 used after risk-adjustment."

NHSN_ALL_ATB_2023 <- Inpatient_2023 %>%
  cbind(NHSN_ALL_ATB_PRED, NHSN_ALL_ATB_EXP) %>%
  select(HospitalID, NHSN_ALL_ATB, NHSN_ALL_ATB_PRED, NHSN_ALL_ATB_EXP, Log_DaysPresent) %>%
  mutate(Days_Present = exp(Log_DaysPresent)) %>%
  select(-Log_DaysPresent)

# PE ratio summary
HospitalID_Summary_ALL_ATB <- NHSN_ALL_ATB_2023 %>%
  group_by(HospitalID) %>%
  summarise(NHSN_ALL_ATB = sum(NHSN_ALL_ATB),
            NHSN_ALL_ATB_PRED = sum(NHSN_ALL_ATB_PRED),
            NHSN_ALL_ATB_EXP = sum(NHSN_ALL_ATB_EXP),
            Days_Present = sum(Days_Present)) %>%
  ungroup() %>%
  mutate(PERatio = NHSN_ALL_ATB_PRED/NHSN_ALL_ATB_EXP,
         DOTperDP = NHSN_ALL_ATB/Days_Present) %>%
  mutate(PERank = rank(PERatio),
         CrudeRank = rank(DOTperDP))
```

© 2025 Goto M et al. JAMA Network Open.

```r
# Create plot (Figure 1E for DOT, Figure 1F for DASC)
ggplot(data = HospitalID_Summary_ALL_ATB)+
  geom_point(aes(x = CrudeRank, y = PERatio)) +
  scale_x_continuous(limits = c(1,117),
                     breaks = c(1,15,30,45,60,75,90,105),
                     name = "Hospital Ranking Based on Crude DOT per DP")+
  scale_y_log10(limit = c(0.4, 2.4),
                name = "P/E Ratio for DOT")+
  geom_hline(yintercept = 1, lty = 2)+
  theme_bw()
```

© 2025 Goto M et al. JAMA Network Open.
