# Supplementary material for: Metrics Selection and Risk Adjustment Methods to Benchmark Inpatient Antibiotic Use
Source: JAMA Netw Open. 2025 Jun 11;8(6):e2514989. doi: 10.1001/jamanetworkopen.2025.14989 (PMC12159770; doi:10.1001/jamanetworkopen.2025.14989)
Supplement: Supplement 2. — Data Sharing Statement [file jamanetwopen-e2514989-s002.pdf]

Data Sharing Statement

Goto. Metrics Selection and Risk Adjustment Methods to Benchmark Inpatient Antibiotic Use. *JAMA Netw Open*. Published June 11, 2025. doi:10.1001/jamanetworkopen.2025.14989

Data
Data available: No
